# The long noncoding RNA *VIM-AS1* and nucleoporin Nup358/RanBP2 regulate SMAD nuclear accumulation during TGF-β signaling

**DOI:** 10.1093/nar/gkaf1526

**Published:** 2026-01-20

**Authors:** Dorival Mendes Rodrigues-Junior, Mohamad Moustafa Ali, Yuka Itoh, Mafalda Sousa Ferreira, Johan Heldin, Hao Fu, André Hoelz, Carl-Henrik Heldin, Aristidis Moustakas

**Affiliations:** Department of Medical Biochemistry and Microbiology, Science for Life Laboratory, Box 582, Biomedical Center, Uppsala University, SE-751 23 Uppsala, Sweden; Department of Medical Biochemistry and Microbiology, Science for Life Laboratory, Box 582, Biomedical Center, Uppsala University, SE-751 23 Uppsala, Sweden; Department of Medical Biochemistry and Microbiology, Science for Life Laboratory, Box 582, Biomedical Center, Uppsala University, SE-751 23 Uppsala, Sweden; Department of Biochemistry, University of Yamanashi, Shimokato 1110, Chuo, Yamanashi 409-3898, Japan; Department of Zoology, Science for Life Laboratory, Stockholm University, Tomtebodavägen 23A, SE-171 65 Stockholm, Sweden; Department of Pharmaceutical Biosciences, Uppsala University, Husargatan 3, SE-75124 Uppsala, Sweden; Department of Biochemistry, University of Yamanashi, Shimokato 1110, Chuo, Yamanashi 409-3898, Japan; Division of Chemistry and Chemical Engineering, California Institute of Technology, Pasadena, CA 91125, United States; Howard Hughes Medical Institute, California Institute of Technology, Pasadena, CA, United States; Department of Medical Biochemistry and Microbiology, Science for Life Laboratory, Box 582, Biomedical Center, Uppsala University, SE-751 23 Uppsala, Sweden; Department of Medical Biochemistry and Microbiology, Science for Life Laboratory, Box 582, Biomedical Center, Uppsala University, SE-751 23 Uppsala, Sweden

## Abstract

The transforming growth factor β (TGF-β) pathway is a developmental signaling network that regulates tissue homeostasis and malfunctions in human diseases, including cancer. TGF-β signals via two receptors, which activate SMAD and alternative signaling pathways. We show that TGF-β induces the expression of the mammalian long noncoding RNA (lncRNA) *VIM-AS1* (*Vimentin antisense RNA1*) variant-2 (v.2) via a transcriptional SMAD-GATA6-SPI1 complex. *VIM-AS1* v.1 and v.2 localize in different cell compartments, including the nuclear border. Unbiased whole transcriptomic analysis and functional gain and loss of function assays establish that *VIM-AS1 v.2* enhances TGF-β signaling. Mechanistically, *VIM-AS1* v.2 interacts with the nucleoporin Nup358/RanBP2, contributing to the binding of Nup358/RanBP2 to SMAD2/3 and enhancing SMAD nuclear accumulation. In the context of cancer biology, *VIM-AS1* did not affect the antiproliferative actions of TGF-β, yet had an impact on the epithelial–mesenchymal transition gene program, and increased the invasion and motility of tumor cells, whereas its silencing sensitized cancer cells to chemotherapeutic agents. The molecular mechanism highlights how a lncRNA can modulate the nuclear pore’s capacity to import SMAD complexes, by facilitating their capture by Nup358/RanBP2 and thereby enhancing nuclear accumulation of SMADs with distinct isoform composition, thus promoting selectively TGF-β signaling responses.

## Graphical Abstract

**Graphical Abstract:**
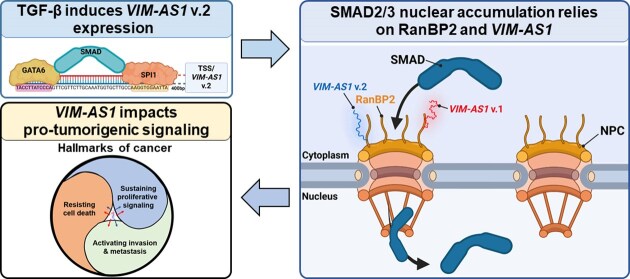

## Introduction

The biology of transforming growth factor β (TGF-β) signaling spans a large spectrum of physiological processes across metazoans and plays important roles in every human tissue [1]. Among the large spectrum of cellular responses to TGF-β signaling are cell cycle arrest and apoptotic induction, phenotypic changes in cell plasticity and differentiation, including immune cell function, fibrogenic remodeling of extracellular matrix, and cell motility [1]. The signaling mechanism proceeds when TGF-β binds to its receptors (type I/TGFβRI and type II/TGFβRII), which are equipped with protein kinase activity. TGFβRI is activated by phosphorylation by TGFβRII and, in turn, phosphorylates two C-terminal serine residues in the receptor-activated SMADs (R-SMADs; SMAD2 and SMAD3), which then form trimeric complexes with SMAD4 [1]. The TGF-β receptor complex additionally recruits adaptor proteins that activate alternative, non-SMAD pathways, including protein kinases, such as MAP kinases, Src and phosphatidylinositol-3´ kinase/Akt [2]. In the absence of ligand-receptor activation, R-SMADs and SMAD4 shuttle in and out of the nucleus. When TGF-β receptor signaling is on, R-SMADs and SMAD4 rapidly accumulate in the nucleus, associate with DNA, transcription factors (TFs), co-activators and co-repressors and modulate gene expression [3]. The highly regulated nucleocytoplasmic shuttling of SMADs is vital for signal transduction and is executed by mechanisms that rely on specific interactions between SMADs, transporter proteins (importins/exportins) and constituent components of the nuclear pore. Accordingly, SMAD2 makes contacts with nucleoporins Nup214/CAN and Nup153 [4]. SMAD3 contacts importin-β1 (KPNB1) via a mechanism facilitated by the Ran GTPase and by the long noncoding RNA (lncRNA) *NORAD* [5, 6]. SMAD4 contacts importin-α (KPNA), importin-7 (IPO7/RANBP7) and importin-8 (IPO8/RANBP8) to translocate into the nucleus [7, 8]. Importins 7 and 8 can additionally bind to multiple SMAD proteins [8]. Export of nuclear SMADs to the cytoplasm is also highly regulated and contributes to the termination of transcriptional signaling in the nucleus, while facilitating multiple cycles of SMAD signaling over time [9]. Export of SMAD3 is mediated by exportin-4 (XPO4) in association with Ran, whereas SMAD4 is exported by exportin-1 (XPO1/CRM1) [10, 11]. Furthermore, RanBP3, an exportin co-factor, participates in SMAD2 and SMAD3 export to the cytoplasm [12]. Thus, SMAD proteins are subject to bidirectional translocation through nuclear pores, while import and export seem to involve a complex, step-wise mechanism of interactions with multiple constituents of the nuclear pore complex (NPC) and importins/exportins [2, 3]. For this reason, understanding the mechanism of SMAD translocation through the NPC [13, 14] requires deeper analysis, as it may involve a sequential ping-pong-like translocation between adjacently ordered components of the NPC, to which both importins and SMADs form transient complexes. An exciting possibility that is supported by recent studies on the variability and plasticity of nuclear pores [15], is that signaling mediators like SMADs do not shuttle randomly via any available NPC, but rather shuttle via selected subsets of NPCs. The elements that define such selective pools of NPCs remain largely unknown.

During their nuclear residence, SMAD complexes engage into the transcriptional induction or repression of protein-coding and nonprotein coding genes, including genes encoding lncRNAs. Transcriptional regulation of lncRNA genes so far is best understood as contributing to the actions of TGF-β in the context of cancer, beyond the multitude of alternative functions of such lncRNAs [16, 17]. Thus, TGF-β-mediated lncRNA induction or repression may regulate cell plasticity, i.e. epithelial–mesenchymal transition (EMT), suppression of immune cell proliferation and differentiation and cancer cell invasion that favors cancer metastasis [16, 17]. To gain more knowledge on functional lncRNAs affecting TGF-β signaling, we previously identified TGF-β-induced lncRNAs in human keratinocytes, such as *CASC2, DSCAM-AS1, KCNIP4-IT1, SOX2-OT*, and *VIM-AS1* [18], but their possible role in TGF-β signaling remains unexplored. We hereby investigated the regulation of expression and the functional relevance of the vimentin antisense 1 (*VIM-AS1*) lncRNA, which is transcribed in an antisense orientation to the *Vimentin* (*VIM*) protein-coding gene. *VIM-AS1* has been described to positively regulate *VIM* expression via an RNA:DNA hybrid R-loop mechanism, facilitating the accessibility of the *VIM* promoter to TFs [19]. *VIM-AS1* function has also been linked to EMT induction in some cancer models by acting as competing endogenous (ce) RNA [20–22]. Here, we elucidated mechanistically how TGF-β signaling engages R-SMAD complexes associated with GATA6 and SPI1 TFs to induce the expression of *VIM-AS1* variant 2. We report the formation of multimeric ribonucleoprotein complexes between the nucleoporin Nup358/RanBP2 and *VIM-AS1*, impacting on the interaction between R-SMADs and Nup358/RanBP2. The latter affects R-SMAD protein accumulation in the nucleus, and thus, our work places *VIM-AS1* as a facilitator of TGF-β signaling.

## Materials and methods

### Cells, reagents, and treatments

Cell lines of human basal triple negative breast cancer (TNBC; MDA-MB-231; HTB-26), luminal breast cancer (ZR-75-1; CRL-1500); lung adenocarcinoma (LUAD; A549; CCL-185); nonsmall cell lung carcinoma (NSCLC; H1299; CRL-5803); human embryo kidney (HEK293T; CRL-3216); cutaneous squamous cell carcinoma (cSCC; A431; CRL-1555) were obtained from the American type culture collection (https://www.atcc.org/). The patient-derived cSCC UT-SCC-12A (primary cutaneous SCC) and UT-SCC-7 (metastatic cSCC) were a generous gift from Andor Pivarcsi, Uppsala University, Sweden. Immortalized human lung epithelial cells, HPL1 were previously described [23]. Primary cultures of human breast fibroblasts (BNF2, BNF3) were a generous gift from Dimitris Kletsas, National Centre for Scientific Research “Demokritos”, Athens, Greece. Human immortalized keratinocytes (HaCaT), human hepatocellular carcinoma (HCC; HLF), and human prostate adenocarcinoma (PRAD; PC3U) cells were previously described [23–25]. These cells were cultured in Dulbecco’s modified Eagle’s medium (DMEM; Sigma–Aldrich AB, Stockholm, Sweden) or Roswell Park Memorial Institute‐1640 (Thermo Fisher Scientific, Stockholm, Sweden), and supplemented with 10% fetal bovine serum (FBS; Biowest, Almeco A/S, Esbjerg, Denmark), and 100 U/ml penicillin and 100 μg/ml streptomycin (Merck/Millipore, Stockholm, Sweden). The patient-derived human glioblastoma (GBM) cells U3017MG (classical subtype) and U3031MG (mesenchymal subtype) were a generous gift from the human glioblastoma cell culture (HGCC; https://www.hgcc.se/), and were cultured as previously described [26]. The nontumorigenic breast epithelial MCF10A cells were cultured in DMEM/F12 (Thermo Fisher Scientific), supplemented with 5% horse serum (Biowest), 20 ng/ml epidermal growth factor (PeproTech EC Ltd/Thermo Fisher Scientific), 100 ng/ml cholera toxin (Sigma–Aldrich AB), 0.5 µg/ml hydrocortisone (Sigma–Aldrich AB), 10 µg/ml insulin (Sigma–Aldrich AB). All cells were kept in a humidified incubator at 37°C and 5% CO_2_. The cell lines were checked for mycoplasma presence and authenticated through STR profiling (Eurofins, Uppsala, Sweden). The cells were starved overnight in serum-free media prior to treatment for different time periods with 5 ng/ml recombinant human TGF-β1 (referred to as TGF-β in the main text; PreproTech EC Ltd/Thermo Fisher Scientific) or 5 µM LY2157299 (TβRi; #15409, Cayman Chemical Co, Ann Arbor, MI, USA). Cycloheximide (CHX; Merck/Millipore), an inhibitor of protein synthesis, was used at 40 μg/ml.

### RNA extraction and reverse transcription-quantitative polymerase chain reaction (RT-qPCR)

RNA extraction and real-time RT-qPCR were performed as previously described [25]. Relative gene expression levels were normalized to *GAPDH* levels. Primer sequences are described in Supplementary Table S1.

### Transcript reference annotation and coding potential

NR_108061.1 *Homo sapiens VIM antisense RNA 1* v.1 (*VIM-AS1* v.1), lncRNA of 1883 nt, and NR_108060.1 *Homo sapiens VIM antisense RNA 1 v.2* (*VIM-AS1 v.2*), lncRNA of 1359 nt were the annotated RNA sequences with validated status in the NCBI database. The Coding-Potential Assessment Tool (CPAT) algorithm was used to predict the coding probability based on the sequence of the transcripts of *TGFB1, VIM*, and *VIM-AS1* v.1 and v.2.

### Correlation analysis of gene expression

RNA-seq data of cancer patients from TCGA were retrieved from the GEPIA2 portal [27] to perform correlations of gene expression in tumor versus normal samples and co-expression of *TGFB1* messenger RNA (mRNA) with lncRNAs (*VIM-AS1* v.2 - ENST00000437232.5, *VIM-AS1* v.1 - ENST00000605833.1, *CASC2, SOX2-OT*, and *DSCAM-AS1*) in LUAD, lung squamous cell carcinoma (LUSC), skin cutaneous melanoma (SKCM), pancreatic adenocarcinoma (PAAD), GBM, leukemia, acute myeloid leukemia (LAML), PRAD or thymoma (THYM). Analyses were based on the Pearson correlation statistic (*P*-value <0.05).

### RNA secondary and tertiary structure prediction

Predictions of lncRNA secondary structure were generated using the Vienna RNA/RNAfold WebServer package [28]. The optimal secondary structure with a minimum free energy (ΔG; kcal/mol) was calculated based on the lncRNA primary sequence. RNA tertiary structure prediction was performed with RNAComposer [29].

### Phylogenetic conservation

To understand the functional conservation of *VIM-AS1*, we used the Genome Browser (access 18 March 2024) to analyze publicly available alignments of 100 vertebrates to the human genome (hg38) and respective conservation scores (PhyloP and PhastCons). The original methods used to generate the alignments, PhyloP and PhastCons scores and phylogenetic trees are provided in detail in the Genome Browser website (https://genome.ucsc.edu/cgi-bin/hgTrackUi?hgsid=3320998303_2ZIWh9f6UquftXufAg5ijUXssejW&db=hg38&c=chr20&g=cons100way). Briefly, genomes of 100 vertebrates were aligned using Multiz [30] and lastz [31]. Conservation scores were derived with PhyloP [32] and PhastCons [33]. This analysis generated positive PhyloP scores that represent sites predicted to be conserved in the alignment, and negative scores, which represent sites under fast evolution (scores are represented as −log *P*-values under a null hypothesis of neutral evolution). PhastCons scores have been calculated and represent the probability of a site being under negative selection and ranging between 0 (less conserved) and 1 (more conserved). Both PhyloP and PhastCons relied on a phylogenetic tree relating the species in the alignment, and in this case, the phylogenetic tree was generated based on ref. [34]. Divergence times for nodes of interest were overlayed in this tree from TimeTree (access 18 March 2024).

### Immunoblotting and immunoprecipitation

Immunoblotting was performed as previously described [26]. The lysis buffer contained complete protease inhibitors (#11697498001, Roche Diagnostics Scandinavia AB, Bromma, Sweden), phosphatase inhibitors (PhosSTOP; #4906837001, Sigma–Aldrich AB), and protein content was determined using the Bradford Reagent (#5000006, Bio-Rad Laboratories Inc., Sundbyberg, Sweden). The proteins were subjected to sodium dodecyl sulphate–polyacrylamide gel electrophoresis (SDS–PAGE), before or after immunoprecipitation with 2.0 µg of the respective primary antibody, captured with Dynabeads™ M-280 sheep antimouse immunoglobulin (IgG) or Dynabeads™ Protein-A (#11202D or #10002D, Thermo Fisher Scientific), followed by immunoblotting. The antibodies used in this study are described in Supplementary Table S2. ImageJ bundled with Java 1.8.0_172 (National Institutes of Health, Bethesda, MD, USA) was used to normalize intensity levels according to the expression of loading control proteins GAPDH, β-Actin, or β-Tubulin.

### Nuclear-cytoplasmic fractionation

Nuclear-cytoplasmic fractionation of RNA samples was performed using the PARIS kit (Thermo Fisher Scientific) based on the manufacturer’s instructions. Briefly, the cells were resuspended in a Cell Fractionation Buffer and kept on ice for 10 min. Lysates containing the nuclear pellet were separated from the supernatant, which has the cytoplasmic fraction and lysed with a Cell Disruption Buffer. The nuclear and cytoplasmic RNAs were isolated with the Lysis/Binding solution, followed by washes with 100% ethanol. The RNA was captured by a filter cartridge and eluted in a pre-heated Elution solution. For the fractionation of nuclear-cytoplasmic proteins, the NXTRACT kit was used, following the manufacturer’s guidelines (#078K4112; Sigma–Aldrich).

### Combined RNA fluorescence *in situ* hybridization (RNA-FISH) and IF assays

In situ hybridization of *VIM-AS1* RNA was performed according to the ACD RNAscope assay-User manual for RNA-FISH protocol for adherent cells using the RNAscope Multiplex Fluorescent Detection kit (#323110, Advanced Cell Diagnostics Bio-Techne, Abingdon, UK). Briefly, the cells were fixed in 4% formaldehyde, permeabilized in 70% (v/v) ethanol and the hybridization steps were performed, using hybridization buffer, containing RNA probes specific for *VIM-AS1* (#1167271), negative control (#310043; *Escherichia coli dapB* mRNA) and positive control (#313901; human *PPIB* mRNA), followed by Opal 690 reagent. A 20zz probe base-pairing with nucleotides 274-1714 of the NR_108061.1 (v.1) RNA sequence was designed and this probe optimally detected both NR_108061.1 (v.1) and NR_108060.1 (v.2). The cells were incubated for each hybridization step at 40°C and then stained with 4’,6-diamidino-2-phenylindole (DAPI; 1:1000; Sigma–Aldrich AB), mounted in ProLong Gold (Thermo Fisher Scientific). The RNAscope Multiplex Immunofluorescence (IF) integrated co-detection reagents were used as stated in the manufacturer’s instructions (Advanced Cell Diagnostics Bio-Techne). Briefly, the cells were incubated overnight at 4°C with rabbit anti-RanBP2 (#ab64276; Abcam, Cambridge, UK), followed by RNA hybridization, signal amplification and incubation with the respective secondary antibody conjugated with Alexa Fluor 488. Nuclei were counterstained with DAPI (1:1000). The images were acquired on a Leica Stellaris 5 confocal microscope with 63×/1.4 oil objective lens (Leica Microsystems).

### Transfections

Once the cells reached 70% of confluence, transfections were performed once or twice with siLentFect (#170-3360, Bio-Rad Laboratories Inc.) for the respective short interfering RNA (siRNA) or antisense oligonucleotides (ASOs) at 20 nM, according to the manufacturer, while Lipofectamine-3000 (#L3000015, Thermo Fisher Scientific) was used for single transfections with 1 µg of the respective plasmid. After 48 h, the cells were collected to validate the transfection and seeded for further experiments. The human‐specific siRNA, ASOs, and plasmids used in this study are described in Supplementary Table S3.

### Genome data analysis

High-throughput chromosome conformation capture (Hi-C) data were processed using HiCExplorer (version 3.7.2) as previously described [35]. Paired-end Hi-C reads (GSE92819, CRA001325; [36]) were first aligned to the human reference genome (GRCh38/hg38) using Bowtie2. BAM files were used to generate binned genome-wide Hi-C contact matrices at different resolutions, with all invalid read pairs (dumped pairs, dangling-end reads, single-end reads, and self-circle pairs) removed. Finally, the matrices were normalized with hicNormalize using the *smallest* method and corrected with the hicCorrectMatrix function using the ICE algorithm. Topologically associating domains (TADs) were identified by hicFindTads with default parameters set at 3 kbp resolution. Chromatin immunoprecipitation sequencing (ChIP-seq) reads (GSE92782, GSE51510, GSE226487, CRA001325) [36] were aligned to the human reference genome (GRCh38/hg38) using Bowtie2, and peaks were called with MACS2 under default parameters. The contact maps and ChIP-seq profiles were visualized using pyGenomeTracks (version 3.9).

### Chromatin immunoprecipitation

MDA-MB-231 cells were stimulated or not with TGF-β1 for 1 h, then fixed with 1% w/v formaldehyde for 10 min at 25°C and quenched with 0.125 M glycine for 5 min with gentle shaking. Chromatin immunoprecipitation (ChIP) experiments were performed as described [37], with 5 μg anti-SMAD2/3 antibody (BD Biosciences, Europe), normal mouse IgG (Merck/Millipore) and primers for qPCR of precipitated *VIM-AS1* and *SERPINE1* DNAs (Supplementary Table S1).

### Motif enrichment analysis

The motif enrichment analysis was performed using Hypergeometric Optimization of Motif EnRichment (HOMER; http://homer.ucsd.edu/homer/motif/) suite v4.11. The *de novo* motif search was performed in a genomic region spanning ± 1 kbp from the *VIM-AS1* v.2 transcription start site (TSS) and the enriched motifs were sorted based on the false discovery rate.

### Plasmids

Human *VIM-AS1* RNAs [NR_108060.1 (v.2) and NR_108061.1 (.v1)] and a mutant of v.2 (v.2Δex2) were reverse transcribed to cDNA and amplified by PCR using cDNA from MDA-MB-231 cells. Other deletion mutants of *VIM-AS1* (v.1Δex1 and v.2Δex4) were generated from the cloned *VIM-AS1* v.1 or v.2. Oligonucleotides for PCR were synthesized with flanking *KpnI* or *XhoI* site and the sequences are shown in Supplementary Table S4. TWIN-Flag(F)-SMAD3 plasmid was constructed by inserting oligonucleotides encoding TWIN-Flag tags (sequence: WSHPQFEKGGGSGGGSGGSAWSHPQFEKDIDYKDDDDKG) into a SMAD3 expression vector using *Bam*H I and *Eco*R I sites. All the constructs have the pcDNA3 backbone and the inserted sequences were confirmed by Sanger sequencing. The constructions of the 3 × HA-RanBP2 plasmids were previously described [38].

### Luciferase assays

Luciferase assays in cells transiently transfected with the CAGA_12_-luciferase promoter-reporter and treated with TGF-β1 for 24 h were performed using the firefly and renilla dual-luciferase assay kit (Biotium, Fremont, CA, USA), as described [25]. Relative normalized luciferase activity is expressed as averages from triplicate determinations with standard error of the mean (SEM). Each experiment was repeated at least twice in triplicate.

### Immuno- and direct fluorescence microscopy

The cells were seeded in a 12-well plate, followed by treatments with TGF-β1 for the indicated time periods. Fixed cells were incubated with primary antibodies in 1% bovine serum albumin/phosphate buffered saline (PBS) overnight at 4°C, followed by incubation with Alexa-Fluor-488 or Alexa-Fluor-546 secondary antibodies (Supplementary Table S2) at a dilution of 1:500 in PBS for 1 h at 25°C. Cell nuclei were stained with DAPI (Sigma–Aldrich AB) at a dilution of 1:1000 in PBS for 10 min at 25°C (Ex/Em 360/460 nm). For F-actin staining, the cells were incubated with phalloidin labeled with FITC (#P5282; Merck/Millipore) at a dilution of 1:250 in PBS for 30 min at 25°C. The cells were examined by an inverted microscope (Nikon-Eclipse Ti-U, Nikon Europe B.V., Amstelveen, The Netherlands), equipped with a CCD camera (Andor multi pixel sCMOS camera, Oxford Instruments, Abingdon, UK) or on a Leica Stellaris 5 confocal microscope (Leica Microsystems). Ten to 15 random pictures were taken with 10× or 20× objectives at the same exposure time. When appropriate, the scores were given in a blind manner.

### Proximity ligation assay

The cells were seeded in an eight-well chamber slide and stimulated with 5 ng/ml TGF-β1 for 0, 15, or 30 min, 1 or 6 h. Cells were then fixed and permeabilized, as described under the IF microscopy section. Proximity ligation assay (PLA) was then performed according to the manufacturer’s instructions (NaveniFlex MR, Navinci Diagnostics, Uppsala, Sweden), a more efficient version of *in situ* PLA [39]. Primary antibodies used are described in Supplementary Table S2. Deconvoluted pictures were taken at least in triplicates per experimental condition, using a Zeiss imager M2 microscope and controlled with Zen 2 (blue edition) software (Zeiss, Jena, Germany). A 63×/1.4 oil objective was used, and pictures were acquired with a Hamamatsu C11440 camera. Excitation of samples was done with an HXP 120 V light source (90% light intensity) and imaged using filter cube sets 49 and 31 from Zeiss, which are suitable for the fluorescence wavelengths of Hoechst (Ex/Em 350–361/454–497 nm) and TexasRed (Ex/Em 590/610 nm). PLA product signal strength shown in the figures has been enhanced for visualization purposes; however, image analysis and quantification have been performed on original images using the CellProfiler software version 3.1.9 [40].

### RNA-seq and gene set enrichment analyses

RNA extraction from A549 biological triplicate samples transfected with siCtrl or si*VIM-AS1* stimulated or not with TGF-β1 for 24 h, was performed using ReliaPrep™ RNA Cell Miniprep System (Promega, Madison, WI, USA). Upon RNA quality assessment, 500 ng of total RNA were subjected to library preparation using the TruSeq stranded total RNA Gold library preparation kit with RiboZero Gold treatment and unique dual indexes, following the manufacturer’s instructions (#1000000040499, Illumina Inc., San Diego, CA, USA). Paired-end sequencing was carried out with v1.5 sequencing chemistry on a NovaSeq-6000 platform using S4 flow cells (Illumina Inc.). Differential gene expression analysis was performed utilizing the DESeq2 Bioconductor package in R, considering any transcript with log_2_ fold-change ±1 and false discovery rate (FDR) < 0.05. Data visualization was done in RStudio v1.4.1717 with R v4.0.5. Gene set enrichment analysis (GSEA) was carried out with the GSEA tool and the molecular signature database MSigDBv6 [41]. Processed RNA-sequencing data and database analysis files are presented in Supplementary Table S5.

### DNA affinity precipitation

The cells were lysed in 0.5% NP-40, 100 mM ethylenediaminetetraacetic acid (EDTA), and 100 mM Tris–HCl, pH 8.0. Lysates were precleared with streptavidin beads for 30 min, incubated for 90 min with biotin-labeled double-stranded oligonucleotides, and for 30 min with streptavidin beads, followed by three washes with lysis buffer; bound proteins were recovered by incubation with 1% SDS, 1 mM DL-dithiothreitol, and subjected to SDS–PAGE, followed by immunoblotting. The DNA sequences represented in the biotinylated oligonucleotides were obtained from the *VIM-AS1*-binding motif indicated by the HOMER Motif analysis. The oligonucleotide sequences were as follows: hs*VIM-AS1*, Forward, Biotin-GTTTACCTTATCCCAGTTCGTTCTTGCAAATGGTGCTTGCCAAGGTGGAATTACAGG; Reverse, -CCTGTAATTCCACCTTGGCAAGCACCATTTGCAAGAACGAACTGGGATAAGGTAAAC-3.

### Chromatin oligo-affinity precipitation

The chromatin oligo-affinity precipitation (ChOP) assay was performed as described before [42] with some modifications. For identification of *VIM-AS1* interacting proteins, A549 cells (20 × 10^6^) were washed with PBS twice, trypsinized, collected by centrifugation and crosslinked for 10 min using 10 ml of 1% formaldehyde in PBS with gentle rotation at 25°C. Samples were briefly sonicated using a Bioruptor sonicator for 20 cycles (30 s on, 30 s off at High Pulse). Following the sonication, the insoluble fragments were removed by centrifugation at a maximum speed for 15 min at 4°C. The cleared solutions were transferred to fresh tubes, and 10% of the lysate was kept to serve as an input. Five different biotinylated oligonucleotides complementary to *VIM-AS1* were pooled with a final concentration of 10 µM and then used for the RNA pull-down. As a control, a pool of three biotinylated oligonucleotides complementing LacZ was used. The oligonucleotides were added to the chromatin solution along with yeast transfer RNA (tRNA; 100 µg/ml), salmon sperm DNA (100 µg/ml); samples were incubated overnight at 4°C with gentle rotation, and then with 35 µl of streptavidin magnetic beads for 3 h at 4°C with gentle rotation, followed by two washes of each low-salt buffer [20 mM Tris–HCl, pH 7.9, 150 mM NaCl, 2 mM EDTA, 0.1% sodium dodecyl sulphate (SDS), 1% Triton X-100, 0.5 mM phenylmethylsulfonyl fluoride, protease inhibitor cocktail and 50 units/ml RNase inhibitor], and high-salt buffer (low-salt buffer, except that NaCl was 500 mM) for 5 min at 4°C with gentle rotation, followed by three washes with PBS at 25°C. The protein complexes were eluted from the beads by incubation with PBS, 0.1% SDS with intermittent mixing at 80°C for 10 min. Triplicate samples, either *VIM-AS1* or LacZ, were subjected to mass spectrometry (MS) analysis. All biotinylated oligonucleotides used in the ChOP pull-down are listed in Supplementary Table S6.

### Proteomic sample preparation

Protein concentrations were determined using a Pierce BCA Protein Assay Kit (Thermo Fisher Scientific) on a Benchmark Plus microplate reader (Bio-Rad Laboratories Inc.). Twenty-five microgram protein amount of each sample was reduced in 100 mM DL-dithiothreitol at 60°C for 30 min. After reduction, the samples were processed using a modified filter-aided sample preparation (FASP)1 method. The reduced samples were transferred onto Microcon-30kDa centrifugal filter units (Merck/Millipore), washed with 8 M urea and then with 0.5% sodium deoxycholate, 50 mM triethylammonium bicarbonate. Free cysteine residues were alkylated using 10 mM methyl methanethiosulfonate for 30 min at 25°C. Proteins were digested with trypsin (Pierce MS grade Trypsin, Thermo Fisher Scientific) in two steps with a final enzyme to protein ratio of 1:50. After centrifugation, peptides were labeled using TMT10plex isobaric label reagents (Thermo Fisher Scientific) according to the manufacturer´s instructions. The labeled samples were pooled into one TMT-set, which was purified using HiPPR detergent removal kit (Thermo Fisher Scientific) according to the manufacturer´s instructions. Remaining sodium deoxycholate was precipitated by acidifying with 10% trifluoroacetic acid. The supernatant was desalted using Pierce Peptide Desalting Spin Columns (Thermo Fisher Scientific). The eluate was evaporated to dryness and dissolved in 3% acetonitrile, 0.1% formic acid for liquid chromatography-mass spectrometry analysis.

### Liquid chromatography-mass spectrometry analysis

The sample was analyzed on an Orbitrap Fusion Lumos Tribrid mass spectrometer interfaced with an Easy-nLC1200 liquid chromatography system (both Thermo Fisher Scientific). Peptides were trapped on an Acclaim Pepmap 100 C18 trap column (100 μm × 2 cm, particle size 5 μm; Thermo Fisher Scientific) and separated on an in-house packed analytical column (35 cm × 75 μm, particle size 3 μm, Reprosil-Pur C18) using a stepped acetonitrile gradient (4%–27%) in 0.2% formic acid over 105 min at a flow of 300 nl/min. The precursor ion mass spectra were acquired at a resolution of 120 000 and an m/z range of 375–1375. Using a cycle time of 3 s, the most abundant precursors with charges 2–7 were isolated with an m/z window of 0.7 and fragmented by collision induced dissociation at 35%. Fragment spectra were recorded in the ion trap at Turbo scan rate. The 10 most abundant MS2 fragment ions were isolated using multinotch isolation for further MS3 fractionation, performed using higher-energy collision dissociation at 65%; the MS3 spectra were recorded in the Orbitrap at 50 000 resolution and an m/z range of 100–500.

### Proteomic data analysis

Data analysis was performed using Proteome Discoverer (Version 2.4, Thermo Fisher Scientific) and Mascot (Version 2.5.1, Matrix Science) as a search engine. The data were matched against the reviewed database of *Homo sapiens* (July 2020, 20 369 entries) allowing one missed cleavage. Precursor mass tolerance was set to 5 ppm and fragment mass tolerance was set to 0.6 Da. Cysteine methylthiolation and TMT6plex were set as fixed modifications, methionine oxidation was set as a variable modification. Percolator was used for PSM validation at a strict FDR of 1%. Only unique peptides were used for protein quantification. The abundance values were normalized to the total peptide amount. The obtained peptide spectrum match scores were used to assess the differential enrichment of significantly abundant proteins in *VIM-AS1* samples versus LacZ samples (Supplementary Table S7). We used DEqMS v1.22.0 [43] and DEP v1.26.0 [44] tools in R to conduct the differential enrichment analysis. The input was formatted as a matrix where rows represent identified proteins and columns represent intensity within the corresponding sample, while a metafile with sample labels was also prepared. Duplicates were removed and unique IDs were assigned to each protein. The data were normalized and analyzed according to the default settings provided by each tool. We applied FDR adjustment to extract the significantly enriched proteins obtained from each tool, and then we compared the results of each tool and proceeded with the commonly identified proteins.

### RNA-binding protein immunoprecipitation

RNA-binding protein immunoprecipitation (RIP) was performed according to the Magna-RIP^TM^ RNA binding protein immunoprecipitation kit (Merck/Millipore) [25]. Beads loaded with 5 μg of anti-RanBP2 antibody (Supplementary Table S2), Pierce™ anti-HA magnetic beads (#88836; Thermo Fisher Scientific) or normal mouse IgG (Merck/Millipore) and primers (Supplementary Table S2) were used. Graphs show average values of relative normalized levels (% input) or enrichment relative to IgG control with standard deviations of at least three biological experiments.

### Docking predictions

HDOCK Server [45] was applied to predict the molecular docking among protein–lncRNA and protein–protein interactions. Representations with the lowest docking score and highest confidence score were shown.

### Pull-down assay

The cells were lysed in 1% NP-40, 150 mM NaCl, 5 mM EDTA, 20 mM Tris–HCl, pH 7.5, containing complete protease and phosphatase inhibitors. The proteins were subjected to SDS–PAGE, before or after pull-down with 10 µl of Strep-Tactin^®^ Sepharose^®^ resin (IBA Lifesciences GmbH, Germany), followed by immunoblotting. The antibodies used in this study are described in Supplementary Table S2.

### Cell culture wound-healing

To evaluate migration, cells pre-treated with TGF-β1 for 48 h were seeded (3 × 10^4^ cells/well) in complete medium into culture-Insert 2 well 24 (#80242; Ibidi GmBh, Gräfelfing, Germany), as previously described [25]. The silicone insert was removed once the cells reached confluence and detached cells were removed by washing twice with PBS, followed by addition of fresh culture media. Wound closure was observed at different time points using a Zeiss Axioplan microscope (objective 10×) with MRC digital camera (Zeiss). Wound surface area was quantified by ImageJ as the percentage of open wound area per condition.

### Matrigel invasion

Transwell inserts (#351152) for 24‐well plates (6.5 mm diameter, 8 μm pore) were coated with 300 μg/ml Matrigel matrix (#734-0269; both from Corning, New York City, NY, USA) diluted in coating buffer (10 mM Tris–HCl, pH 8.0, 0.12 M NaCl) and incubated at 37°C for 1 h. Cells (5 × 10^4^) were seeded in serum‐free DMEM in the upper chamber, and DMEM/10% FBS was placed in the lower chamber and incubated at 37°C to allow the invasion through the Matrigel barrier for 18 h. After incubation, the inserts were fixed in methanol and stained with DAPI (1:1000; Sigma–Aldrich AB). Nuclei that had passed the inserts were counted in 10 pictures per insert, taken with the 20 × objective, using ImageJ. Data were expressed as the percentage of invasion based on the ratio of the mean number of cells invading through Matrigel matrix per mean number of cells in the uncoated support.

### Zebrafish extravasation

Staging and embryo production of Tg(Fli1:EGFP) zebrafish (*Danio rerio*), whose vasculature is marked in green, were performed according to routine procedures in the facility. Empirical determination of sample size that provided power for discrimination between conditions, was used. For this reason, 200 embryos were injected per condition in order to reach a final number of viable embryos of more than 100. No randomization method was applied and the microinjector was blinded to the groups of injected cancer cells. A549 cells treated or not with TGF-β1 were stained with 4 ng/μl CM-Dil Dye (Thermo Fisher Scientific) for 30 min at 37°C. At 48 h post-fertilization, approximately 400 CM-Dil Dye-labeled cells in the presence or absence of doxorubicin (Dox; 5 µM) were micro-injected. Based on pre-established criteria, only microscopy-verified, correctly injected and viable zebrafish were retained at 34°C and imaged automatically (ImageXpress Nano, Molecular Devices, San Jose, CA, USA) at 24 and 48 h post-implantation; cells extravasated from the circulation at the posterior part of 150 zebrafish per experimental condition were counted.

### Cell viability

Cells were treated with Dox (#D1515, Sigma–Aldrich AB) or Taxol (#T7402, Sigma–Aldrich AB). Drug toxicity was assessed after 48 h treatment by PrestoBlue HS Cell Viability Reagent (#P50200, Thermo Fisher Scientific). The fluorescence units from treated cells were normalized relative to those of vehicle-treated (0.1% dimethyl sulfoxide; DMSO) control cells. The 50% inhibitory concentration (IC_50_) for the drugs was estimated based on dose-response curves. Experiments were performed in triplicates and data are expressed as mean ± SEM.

### Mitochondrial transmembrane potential

Mitochondrial membrane potential was analyzed fluorimetrically with MitoTracker™ Red CMXRos (#M7512; Thermo Fisher Scientific). Briefly, after 48 h treatment with the compounds in the absence or presence of Dox or Taxol, the cells were incubated with 500 nM final probe concentration in Hank’s balanced salt solution without phenol red for 30 min at 37°C, detached with trypsin, washed with PBS, and resuspended in the same media, transferred in duplicate into a 96-well plate, and recorded the fluorescence (Ex/Em 579/599 nm). Data are expressed as a percentage relative to the control after correction for protein content.

### Statistical analyses

All data (except when otherwise indicated) are presented as the mean ± SEM. Comparisons were performed using one-way or two-way analysis of variance (ANOVA), followed by multiple paired comparisons conducted by means of the Tukey’s or Bonferroni’s post-test method when applicable. The Mann–Whitney test was used to evaluate associations between gene expression and chemotherapy treatment response. Additional statistical methods are explained in individual figure legends. The data were analyzed with GraphPad Prism 10.1 (GraphPad Software, San Diego, CA, USA). A *P*-value <0.05 was considered statistically significant.

## Ethics approval

The experiments do not involve human subjects. The zebrafish experiments were performed at the core facility at Karolinska Institutet, Stockholm, Sweden, which holds ethical permits from the Stockholm Prefecture Ethics Committee.

## Results

### TGF-β induces *VIM-AS1* v.2 expression

**Figure 1.**
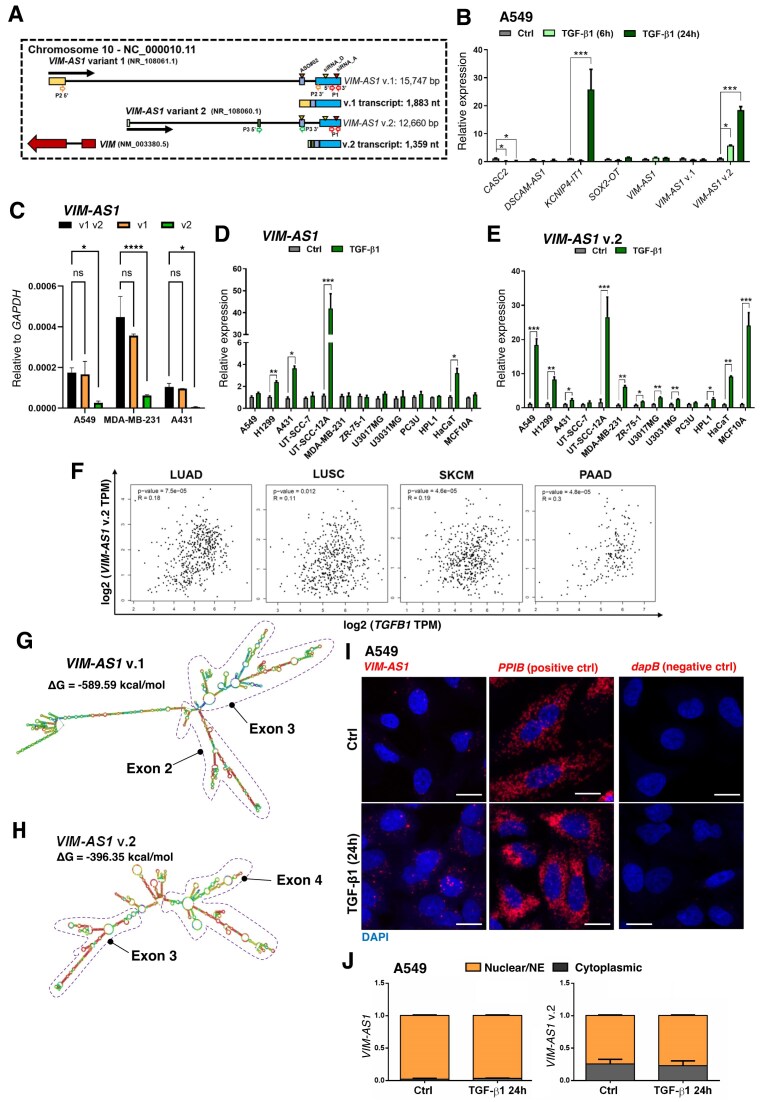
TGF-β induces expression of *VIM-AS1* v.2. (**A**) Schematic representation of the organization of the *VIM-AS1* gene. Exons are shown as boxes and introns as lines. Black arrows indicate the direction of the antisense transcription and the red arrow corresponds to the *VIM* mRNA transcript. The *VIM-AS1* transcriptional variants (v.1 and v.2) on the hg38 genome sequence (chromosome 10) are shown along with nucleotide (nt) numbering, the pair of primers (P1, v.1 and v2; P2, v.1; and P3, v.2), siRNAs and ASO used in the study. (**B**) RT-qPCR analysis of the indicated lncRNA levels in A549 cells upon stimulation with 5 ng/ml TGF-β1 for 6 and 24 h. Values represent fold-change of RNA expression normalized to *GAPDH* and expressed relative to the unstimulated control level (Ctrl). (**C**) RT-qPCR analysis of the *VIM-AS1* using v.1 and v.2 (black), v.1 (yellow) and v.2 (green) levels in the indicated cell models. Values represent fold-change of lncRNA expression normalized to *GAPDH*. (**D, E**) RT-qPCR analysis of the *VIM-AS1* levels (D: v.1 and v.2; E: v.2) in the indicated cell models stimulated or not with 5 ng/ml TGF-β1 for 24 h. Values represent fold-change of RNA expression normalized to *GAPDH* and expressed relative to the unstimulated control level (Ctrl). (**F**) Pearson correlation analysis of *VIM-AS1* v.2 expression in LUAD, LUSC, SKCM, and PAAD relative to *TGFB1* expression, measured as transcripts per million transformed by log_2_; data were obtained from TCGA. *P*- and R-values are indicated. (**G, H**) Predicted secondary structures with the lowest required ΔG of *VIM-AS1* v.1 (G) and v.2 (H) generated by RNAfold. (**I**) RNAscope for *VIM-AS1, PPIB* (positive control) and *dapB* (negative control) in A549 cells stimulated or not with 5 ng/ml TGF-β1 for 24 h. Images were acquired with a Leica Stellaris 5 confocal microscope. Scale bar 25 µm. (**J**) Expression levels of *VIM-AS1* lncRNA, in nuclear/nuclear envelope (NE - orange) and cytoplasmic (gray) fractions of A549 lysates upon stimulation or not with 5 ng/ml TGF-β1 for 24 h. Gene expression is normalized relative to the housekeeping gene *GAPDH* from the input. Data in panels (B), (C), (D), (E), and (J) are presented as mean values of three biological replicates ± SEM, in technical triplicates and *P*-values are shown based on two-way ANOVA, followed by multiple paired comparisons conducted using Bonferroni’s post-test method: *P ≤.05; **P ≤.01; ***P ≤.001; ****P ≤.0001.

An earlier screen in nontumorigenic, human keratinocytes, identified the lncRNAs *CASC2, DSCAM-AS1, KCNIP4-IT1, SOX2-OT*, and *VIM-AS1*, as TGF-β-responsive transcripts [18]. Notably, these lncRNAs harbor multiple variants (v.) generated by alternative transcription initiation or splicing. For instance, *CASC2* and *SOX2-OT* each include six deposited variants, *DSCAM-AS1* four variants, *VIM-AS1* two variants, while only one variant of *KCNIP4-IT1* have been deposited in Gencode (as of March 2025). We sought to validate the impact of TGF-β stimulation on the expression of these lncRNAs in three different tumor cell lines, using variant-specific primer-pairs for RT-qPCR. We thus analyzed *CASC2* v.1 and v.3, *DSCAM-AS1* v.1-4, *SOX2-OT* v.1 and v.5, and *VIM-AS1*, the latter based on one primer-pair common for the last exon of both variants (P1), one primer-pair specific for exons-1 and -3 of v.1 (P1) and another primer-pair specific for exons-2 and -3 of v.2 (P3) (Fig. 1A). *VIM-AS1* v.2 expression was induced by TGF-β in LUAD (A549), cSCC (A431), and TNBC (MDA-MB-231) cells with cell type-specific kinetics (Fig. 1B, and Supplementary Fig. S1A–C). Notwithstanding, *VIM-AS1* v.1 was induced strongly in A431 cells (Supplementary Fig. S1A), whereas *KCNIP4-IT1* was upregulated exclusively in A549, upon 24 h of TGF-β stimulation (Fig. 1B). In contrast, *CASC2* v.1 and v.3 were downregulated by TGF-β in A549 and MDA-MB-231 cells (Fig. 1B and Supplementary Fig. S1B), while the expression of *DSCAM-AS1* and *SOX2-OT* was not affected significantly by TGF-β activation in these cancer cells.

Using the variant-specific primer-pairs (P2) for *VIM-AS1* v.1 and (P3) for v.2, we observed more abundant expression of v.1 compared to v.2 in A549, MDA-MB-231, and A431 cells (Fig. 1C). We then expanded the panel of human tumor cell lines, i.e. H1299 (NSCLC); UT-SCC-7 and UT-SCC-12A (patient-derived cSCC); ZR-75-1 (luminal breast cancer); U3017MG and U3031MG (patient-derived GBM); PC3U (PRAD); and HLF (HCC), as well as nontumorigenic human cells, i.e. HaCaT (keratinocytes); MCF10A (breast epithelial); BNF2 and BNF3 (breast fibroblasts); and HPL1 (lung epithelial) cells, to characterize *VIM-AS1* expression. In accordance with Fig. 1C, all these cells expressed detectable and variable levels of *VIM-AS1* v.1/v.2 (exon-3/-4), which were overall about 10- to 30-fold higher than v.2 (exon2–3) expression alone (Supplementary Fig. S1D and E). We also examined the effect of TGF-β stimulation and found that v.1 was induced by TGF-β in H1299, UT-SCC-12A, A431 and HaCaT cells (Fig. 1D). In contrast, TGF-β induced *VIM-AS1* v.2 expression in all 13 cell models, except for UT-SCC-7 and PC3U cells (Fig. 1E), suggesting preferential upregulation of v.2.

In line with TGF-β inducing *VIM-AS1* v.2 expression in several cancer cells, we found a positive and significant correlation between *TGFB1* mRNA and *VIM-AS1* v.2 expression in different tumor types across TCGA dataset (Fig. 1F and Supplementary Fig. S1F). Likewise, the mRNA expression of the protein coding gene *Vimentin* (*VIM*), whose transcription is induced by TGF-β [46] was significantly correlated with *TGFB1* levels in LUAD, LUSC, SKCM, and PAAD patients (Supplementary Fig. S1G). Correlations between *TGFB1* mRNA and *VIM-AS1* v.1 expression also revealed positive and significant associations, but not for *CASC2, SOX2-OT* or *DSCAM-AS1* in LUAD, LUSC, SKCM and PAAD patients (Supplementary Fig. S1H–K). Among all lncRNA variants analyzed, and in the absence of *KCNIP4-IT1* data in TCGA, we conclude that *VIM-AS1* expression can be distinguished by its correlation with *TGFB1* expression, in addition to being upregulated in response to TGF-β in various cancer cell lines.

### *VIM-AS1* is a mammalian lncRNA localized proximally to the nuclear envelope and extending towards the cytoplasm

*VIM-AS1* v.1 spans three exons and two introns (NR_108061.1, 1883 nt long spliced transcript) on chromosome 10 (NC_000010.11), while v.2 encompasses four exons and three introns (NR_108060.1, 1359 nt long spliced transcript) (Fig. 1A). Even though *VIM-AS1* v.1 and v.2 share the last two exons, these RNAs have distinct predicted secondary structures with a lower ΔG required for v.1 in comparison to v.2, which can be relevant for their corresponding functions (Fig. 1G, H). Moreover, the sequence of human genome (hg38) chromosome 10 encoding *VIM, VIM-AS1* v.1 and v.2, aligned well with the syntenic region in 100 vertebrates. This phylogenetic analysis revealed relatively high conservation scores (PhyloP and PhastCons) for most exons of the protein-coding *VIM* across fish, bird, reptile and mammalian species, while the exons of the two *VIM-AS1* variants showed high conservation only among mammals (Supplementary Fig. S2; note the high conservation of the 30 kbp locus, including introns among primates). Furthermore, *VIM-AS1* v.1 and v.2 lack protein-coding capacity when compared to *TGFB1* and *VIM* mRNA sequences as measured by CPAT (Supplementary Fig. S3A).

RNA *in situ* hybridization (RNAscope assay) revealed the presence of *VIM-AS1* in speckles adjacent to the nuclear envelope of A549 and MDA-MB-231 cells, which were more prominent after TGF-β stimulation, with individual speckles also decorating nucleoplasmic regions, while some extended towards the cytoplasm (Fig. 1I and Supplementary Fig. S3B). We confirmed the subcellular localization of *VIM-AS1* using a fractionation technique that preserves nuclear architecture, including the nuclear envelope and its cytoplasmic constituents. Thus, the reported nuclear fractions contain traces of cytoplasmic constituents attached to the nuclear envelope border. Both variants were enriched in the nuclear/nuclear envelope (NE) fraction of all five cell types tested (Fig. 1J and Supplementary Fig. S3C–G), thus agreeing with previous data in colon adenocarcinoma cells [19], and resembling the strictly nuclear lncRNA *MALAT1* [47], serving as a positive control (Supplementary Fig. S3D–G). We conclude that *VIM-AS1* v.1 and v.2 are synthesized in the nucleus, partition primarily in nuclear/nuclear envelope fractions and can be imaged to localize at the cytoplasmic face of the nuclear envelope and also extending to some extent in the cytoplasm.

### TGF-β engages three transcriptional inputs, SMAD, GATA6, and SPI1, to induce *VIM-AS1* v.2 expression

**Figure 2.**
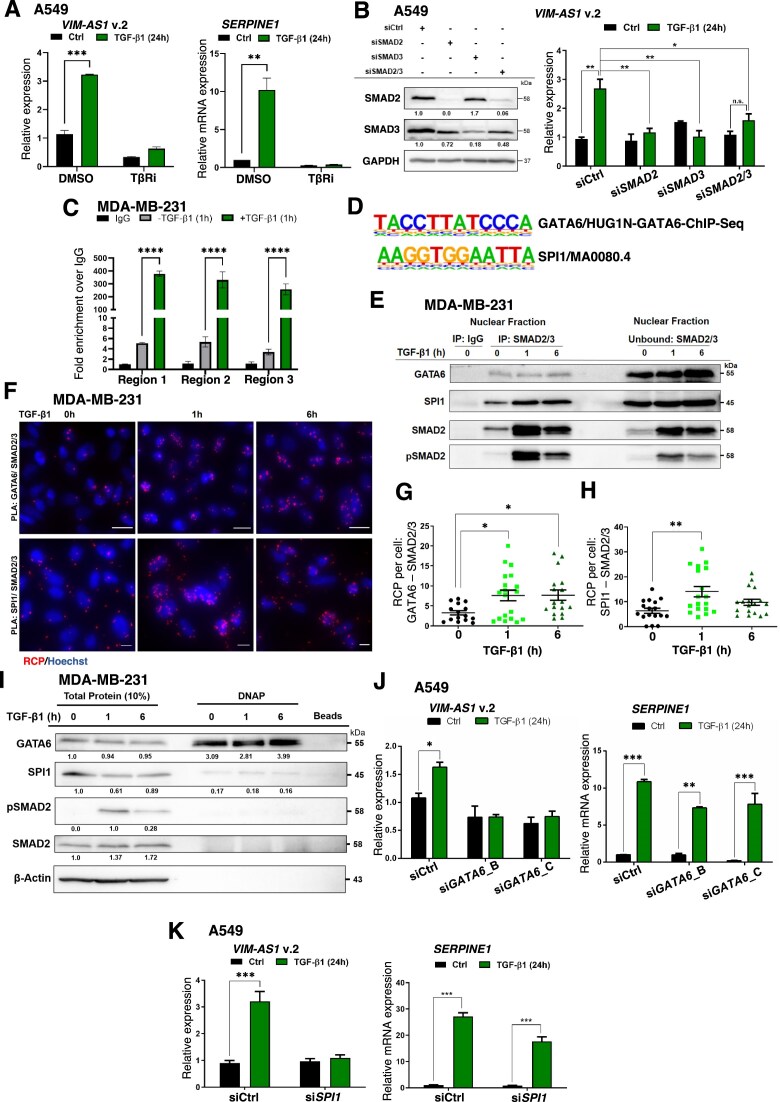
TGF-β induces *VIM-AS1* v.2 expression via a SMAD-GATA6-SPI1 complex. (**A**) RT-qPCR of *VIM-AS1* v.2 and *SERPINE1* in A549 cells stimulated with vehicle (Ctrl), 5 ng/ml TGF-β1, 5 µM LY2157299 TGFβRI inhibitor (TβRi) or combination of TGF-β1 with TβRi for 24 h. (**B**) (Left) Protein expression levels of SMAD2, SMAD3 and GAPDH (as loading control) in A549 protein extracts transiently transfected with the indicated siRNAs, and densitometric values normalized to the control siRNA (siCtrl). (Right) RT-qPCR analysis of *VIM-AS1* v.2 levels in A549 cells transiently transfected with the indicated siRNAs and stimulated with 5 ng/ml TGF-β1 for 24 h or not (Ctrl). (**C**) ChIP-qPCR analysis for SMAD2/3 occupancy to the *VIM-AS1* promoter in MDA-MB-231 cells stimulated or not with TGF-β1 for 1 h. Control IgG immunoprecipitation data are also shown. Error bars represent ± SEM for the fold-enrichment relative to the IgG control from three different experiments. (**D**) Homer analysis of significant DNA binding motifs on the *VIM-AS1* v.2 promoter sequence for GATA6 and SPI1. (**E**) Protein complex formation between SMAD2/3 with GATA6 or SPI1. MDA-MB-231 cells were incubated with vehicle (−) or 5 ng/ml TGF-β1 for 1 and 6 h and the nuclear protein fraction lysates were immunoprecipitated (IP) with SMAD2/3-specific antibody or nonspecific IgG, followed by SDS–PAGE and immunoblotting with GATA6, SPI1, SMAD2, and pSMAD2 antibodies. Nuclear proteins not bound to the SMAD2/3 antibody are shown, for comparison. Representative immunoblots of three independent biological replicates along with molecular mass markers in kDa are shown. (**F**) PLA was used to validate the co-localization of SMAD2/3 with GATA6 or SPI1 in MDA-MB-231 cells incubated with vehicle (0 h) or 5 ng/ml TGF-β1 for 1 and 6 h. Nuclei are shown in blue (Hoechst), and PLA rolling circle amplification product (RCP) in red. (**G, H**) PLA quantification of the co-localization between SMAD2/3 with GATA6 (**G**) or SPI1 (**H**) in MDA-MB-231 cells is shown. (**I**) DNA affinity precipitation (DNAP) of endogenous GATA6, SPI1 and (p)SMAD2 in total protein extract from MDA-MB-231 cells incubated with vehicle (−) or 5 ng/ml TGF-β1 for 1 and 6 h using the specific DNA oligonucleotides of the *VIM-AS1* v.2 promoter. Input shows total cell lysate from the same cells prior to application to the biotinylated oligonucleotides, and beads show negative control of streptavidin beads with the cell lysate in the absence of *VIM-AS1* v.2 DNA. (**J, K**) RT-qPCR analysis of *VIM-AS1* v.2 and *SERPINE1* levels in A549 cells transiently transfected with siCtrl or the indicated siRNAs against *GATA6* (**J**) or *SPI1* (**K**) and stimulated with 5 ng/ml TGF-β1 for 24 h or not (Ctrl). The RT-qPCR values in panels (A), (B), (J), and (K) represent fold-change of RNA expression normalized to *GAPDH*, expressed relative to the unstimulated control levels (siCtrl and Ctrl, respectively), and presented as mean values of at least two biological replicates ± SEM, in technical triplicates. *P*-values shown in panels (A)–(C), (J), and (K) are based on two-way ANOVA, followed by multiple paired comparisons conducted by means of Bonferroni’s post-test method. The PLA data quantification (G, H) are presented as mean values of individual micrographs from at least two biological replicates ± SEM and the *P*-values are shown based on one-way ANOVA, followed by multiple paired comparisons conducted using Bonferroni’s post-test method. *P*-values: *P ≤.05; **P ≤.01; ***P ≤.001; ****P ≤.0001.

Treatment with a TGFβRI kinase inhibitor (TβRi: LY2157299) blocked TGF-β-induced *VIM-AS1* v.2 expression, including induction of the established TGF-β-regulated *SERPINE1* mRNA (Fig. 2A and Supplementary Fig. S4A–F). Silencing SMAD2 and SMAD3, individually or in combination, impaired TGF-β-induced *VIM-AS1* v.2 expression in the same cell types (Fig. 2B and Supplementary Fig. S4G–K). These results demonstrate that TGF-β receptor-SMAD signaling is required to induce *VIM-AS1* v.2 expression in several cancer and nontumorigenic cells. To identify enhancer regions involved in TGF-β–induced *VIM-AS1* v.2 expression, we examined the TAD containing *VIM-AS1* v.1, v.2 and *VIM* in A549 LUAD cells using Hi-C (GSE92819, CRA001325) and CTCF ChIP-seq data (GSE92782) [36, 48]. This TAD spans ∼140 kbp on chromosome 10 and includes a single protein-coding gene, *VIM*, in its entirety, but also the first exon and small part of the first intron of the tRNA aspartic acid methyltransferase 1 (*TRDMT1*) (Supplementary Fig. S5A). Since *VIM-AS1* v.2 induction depends on receptor-SMAD signaling (Fig. 2B), we searched for SMAD3-binding sites within the TAD using SMAD3 ChIP-seq data (GSE51510) after TGF-β stimulation [36]. A relatively broad region exhibiting strong SMAD3 binding was identified upstream of the *VIM-AS1* v.2 TSS (Supplementary Fig. S5A). BRD4 ChIP-seq data (GSE226487) showed enrichment at the same region, suggesting a super-enhancer formation. This region also covered the v.1 TSS and spanned the first two exons and two introns of *VIM*. A second, narrower SMAD3-bound peak was also mapped approximately 30 kbp downstream the 3’ end of v.1/v.2 (Supplementary Fig. S5A). ChIP in MDA-MB-231 cells using three adjacent primer-pairs within the ChIP-seq peak (Supplementary Fig. S5A and B), confirmed the association of SMAD2/3 with the identified enhancer of *VIM-AS1* v.2, where the enrichment for SMAD2/3 upon stimulation with TGF-β for 1 h was stronger than the enrichment of SMAD2/3 to the *SERPINE1* promoter, a hallmark TGF-β-regulated gene (Fig. 2C and Supplementary Fig. S5C).

Since SMAD complexes interact with TFs and chromatin factors to regulate gene expression, we inspected the proximal (±1 kbp) to the v.2 TSS sequence and identified distinct GATA6- and SPI1- but no SMAD-binding motifs in the same region (∼400 bp from the v.2 TSS) (Fig. 2D and Supplementary Fig. S6A). Interestingly, GATA6 and SPI1 are known to cooperate with SMAD2/3 in different biological contexts [49, 50]. We therefore confirmed that GATA6 and SPI1 proteins were expressed in all cell types examined (except PC3U cells that lacked SPI1 expression; Supplementary Fig. S6B). Furthermore, GATA6 was exclusively nuclear and SPI1 showed widespread distribution between the nucleus and cytoplasm (Supplementary Fig. S6C and D). Hence, we used two independent methods to test whether SMAD2/3 could form complexes with GATA6 or SPI1. First, SMAD2/3 immunoprecipitation from MDA-MB-231 nuclear protein fractions confirmed the formation of GATA6-SMAD2/3 and SPI1-SMAD2/3 complexes (Fig. 2E and Supplementary Fig. S6E), while GATA6 did not show any association with SPI1 (Supplementary Fig. S6F; note that SPI1 has an apparent molecular size of 45 kDa and the figure indicates strong IgG detection, labeled with stars). Then, PLA of SMAD2/3 with GATA6 or SPI1 validated the co-immunoprecipitation results, further revealing a significant increase in the amount of GATA6-SMAD2/3 and SPI1-SMAD2/3 complexes in the nucleus of MDA-MB-231 cells after TGF-β stimulation (Fig. 2F–H and Supplementary Fig. S6G).

We then investigated formation of higher order protein complexes between endogenous GATA6, SPI1, SMAD2, and synthetic biotinylated double-stranded oligonucleotides spanning the GATA6 and SPI1 motifs of the *VIM-AS1* v.2 promoter, using DNAP (Fig. 2I). GATA6, and to a lower degree SPI1 bound directly to the *VIM-AS1* v.2 promoter DNA (Fig. 2I). SMAD2 failed to bind in agreement with the lack of SMAD-binding motifs in the selected DNA sequences (Fig. 2I). Furthermore, upon GATA6 knockdown using two independent siRNAs, 24 h TGF-β stimulation failed to enhance significantly *VIM-AS1* v.2 expression in A549 and MDA-MB-231 cells (Fig. 2J and Supplementary Fig. S7A and B). Similarly, SPI1 silencing also abrogated *VIM-AS1* v.2 expression induced by TGF-β (Fig. 2K and Supplementary Fig. S7C–E). Notably, GATA6 or SPI1 silencing did not impair *SERPINE1* or *VIM* mRNA expression induced by TGF-β or the basal levels of *VIM-AS1* (Fig. 2J and K, and Supplementary Fig. S7B and E–G). The absence of SPI1 expression in PC3U cells is consistent with the failure of TGF-β to induce *VIM-AS1* v.2 in these cells (Fig. 1E). Altogether, these data show that TGF-β receptor-SMAD signaling induces *VIM-AS1* v.2 expression in several cancer and nontumorigenic cells through the formation of complexes between receptor-SMADs with GATA6 and SPI1, which bind specific *VIM-AS1* v.2 enhancer-promoter sequences upstream from the v.2 TSS.

### *VIM-AS1* promotes TGF-β signaling and EMT

**Figure 3.**
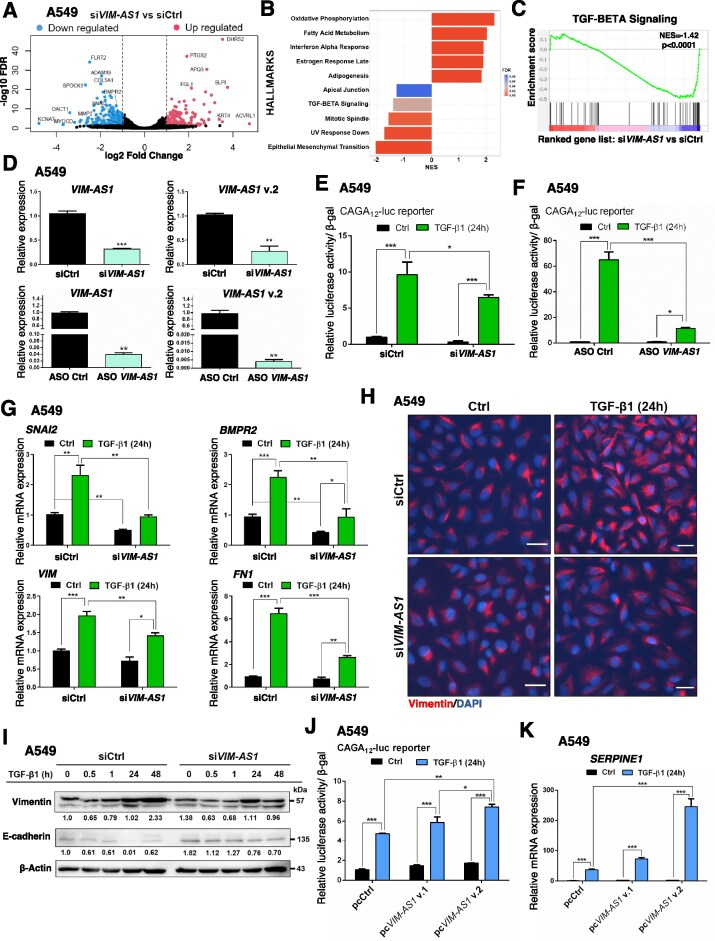
*VIM-AS1* promotes TGF-β signaling and EMT. (**A**) Volcano plot depicting the up- (red) and down- (blue) regulated genes in A549 cells transiently transfected with si*VIM-AS1* in comparison to control siRNA (siCtrl). The vertical lines indicate the expression fold-change threshold (−1 ≤ log_2_ ≥ 1). (**B, C**) The GSEA-Hallmark database indicates a strong downregulation of EMT and TGF-β signaling after *VIM-AS1* silencing (B), with details shown in panel (C). (**D**) RT-qPCR analysis of *VIM-AS1* levels in A549 cells transiently transfected with the indicated control siRNA (siCtrl), si*VIM-AS1*_D, ASO Ctrl or ASO *VIM-AS1* (#02). Values represent fold-change of *VIM-AS1* expression normalized to *GAPDH* and expressed relative to the Ctrl level. (**E, F**) Relative luciferase activity generated in A549 cells transiently transfected with siCtrl or si*VIM-AS1* (E), or ASO Ctrl or ASO *VIM-AS1* (F) by additional transfection of the TGF-β-inducible CAGA_12_-luc reporter, normalized to β-galactosidase activity generated by a co-transfected reporter, after stimulation of the cells with vehicle (Ctrl) or 5 ng/ml TGF-β1 for 24 h. Data are presented as mean values of three biological replicates ± SEM, each in technical duplicates. (**G**) RT-qPCR analysis of the indicated mRNA levels in A549 cells transiently transfected with siCtrl or si*VIM-AS1*. (**H**) Representative IF microscopy pictures of A549 cells transiently transfected with siCtrl or si*VIM-AS1* and stimulated with vehicle (Ctrl) or 5 ng/ml TGF-β1 for 24 h. The Vimentin protein (red) and nuclei (DAPI; blue) are labeled. Scale bars, 50 µm. (**I**) Protein expression levels of the indicated EMT marker proteins in cellular extracts of A549 cells that were transiently transfected with siCtrl or si*VIM-AS1* and stimulated or not with 5 ng/ml TGF-β1 for the indicated periods. Densitometric values are normalized to unstimulated control. Representative immunoblots of three independent biological replicates along with molecular mass markers in kDa are shown. (**J**) Relative CAGA_12_-luciferase activity in A549 cells overexpressing *VIM-AS1* and selected by neomycin, followed by stimulation with 5 ng/ml TGF-β1 for 24 h. (**K**) RT-qPCR analysis of *SERPINE1* mRNA levels in A549 cells overexpressing *VIM-AS1* and selected by neomycin as in panel (I). Values in panels (D), (G), and (K) represent fold-change of RNA expression normalized to *GAPDH* and expressed relative to the respective Ctrl level. The data shown in panel (D)–(G), (J), and (K) are presented as mean values of at least three biological replicates ± SEM, in technical triplicates. *P*-values in panel (D) are shown based on unpaired student’s *t*-test with Welch’s correction. *P*-values in panels (E)–(G), (J), and (K) are shown based on two-way ANOVA, followed by multiple paired comparisons conducted by means of Bonferroni’s post-test method. *P*-values: *P ≤.05; **P ≤.01; ***P ≤.001.

*VIM-AS1* has been proposed to promote EMT in tumor cells [19–22, 51, 52]. However, whether *VIM-AS1* impacts TGF-β signaling, a major inducer of EMT [53], has not been elucidated. Therefore, to identify downstream signaling pathways affected by *VIM-AS1*, we transiently silenced both *VIM-AS1* variants (si*VIM-AS1*) in A549 cells (Supplementary Fig. S8A–C) and compared their transcriptomic profiles with that of control (siCtrl) cells in an unbiased manner. The analysis revealed 351 differentially expressed genes with the TGF-β/SMAD target genes *BMPR2, SNAI2* (*SLUG*), and *MMP2* being remarkably downregulated upon *VIM-AS1* silencing (log_2_ fold-change ≥ ±1 and FDR < 0.05; Fig. 3A and Supplementary Fig S8D). GSEA of cancer hallmarks showed EMT (normalized enrichment score - NES = −2.06), UV response (NES = −1.74), mitotic spindle (NES = −1.59), and TGF-β signaling (NES = −1.42), as the top processes downregulated by the absence of *VIM-AS1*, while oxidative phosphorylation was the most highly upregulated process (NES = 2.30) (Fig. 3B and C, and Supplementary Fig. S8E). Likewise, we performed transcriptomic analysis of A549 cells stimulated with TGF-β for 24 h (Supplementary Figs S8D and S9A). As expected, TGF-β activation affected the expression of 1887 genes, including the TGF-β/SMAD-inducible genes *SERPINE1, MMP2, Fibronectin* (*FN1*), *VIM*, and *VIM-AS1* (Supplementary Fig. S9A). EMT (NES = 2.37) and TGF-β signaling (NES = 1.59) were among the top five cancer hallmarks correlated with the TGF-β-inducible group of genes based on GSEA (Supplementary Fig. S9B and C). On the other hand, TGF-β stimulation failed to induce the expression of similar gene groups in the absence of *VIM-AS1*, leading to the dysregulation of 127 transcripts relative to the 1887 differentially expressed genes under control conditions (Supplementary Fig. S8D). In the absence of *VIM-AS1*, the EMT hallmark was significantly downmodulated (NES = −1.39) (Supplementary Fig. S9E and F), suggesting that *VIM-AS1* silencing has an impact on the expression of genes associated with TGF-β-induced EMT. Additionally, UV response genes were clearly negatively enriched in the absence or presence of TGF-β stimulation, indicating a strong *VIM-AS1*-dependent set of genes, whose regulation is independent from TGF-β signaling (Fig. 3B and, Supplementary Figs S8E and S9F). Conversely, pro-inflammatory response signatures (TNFα/NFκB and Interferon-α) were positively enriched in the absence of *VIM-AS1* (Fig. 3B and, Supplementary Fig. S9E and F). Our cumulative transcriptomic data indicated that *VIM-AS1* affects the expression of several genes, and most profoundly impacts on selective TGF-β responses.

To provide mechanistic validation to the latter set of results, we monitored TGF-β signaling via the CAGA_12_-luciferase reporter that measures SMAD transcriptional activity, after *VIM-AS1* silencing, using either independent siRNA or antisense oligonucleotides (ASOs) complementary to both variants (Figs 1A and 3D, and Supplementary Fig. S10A–E). Analysis of different cell types confirmed the transcriptomic results, since the reporter activation induced by TGF-β was decreased significantly upon *VIM-AS1* depletion in comparison to the respective controls (Fig. 3E and F, and Supplementary Fig. S10F–I). Furthermore, RT-qPCR assays in multiple cell types showed that *VIM-AS1* knockdown did not perturb TGF-β receptor (*TGFBRI* or *TGFBRII*) mRNA expression but reduced significantly the TGF-β-induced expression of endogenous *SNAI2, BMPR2, FN1, SERPINE1, MMP2*, and *MMP10* (Fig. 3G and Supplementary Fig. S11). Moreover, *VIM-AS1* knockdown reduced basal *SNAI2, BMPR2* and *MMP2* mRNA expression in distinct cell models (Fig. 3G and Supplementary Fig. S11), which also agrees with the RNAseq profiles (Fig. 3A). In contrast to previous reports showing that *VIM-AS1* regulates *VIM* expression in colorectal cancer cells [19], the basal level of *VIM* mRNA and protein (measured in the total cell population by immunoblot and in individual cells by IF) in two cancer cell models, was not affected by the reduction of *VIM-AS1*, whereas the inducibility of *VIM* by TGF-β was partially inhibited (Fig. 3G–I, and Supplementary Figs S11B and S12A), pointing again to TGF-β signaling as a key phenotypic target of *VIM-AS1*. It should be noted though, that every individual gene response to TGF-β signaling incorporates unique transcriptional and chromatin complexes beyond the central SMAD signal, which explains why not every gene response is affected to the same degree once *VIM-AS1* is silenced.

To demonstrate the impact of *VIM-AS1* silencing on the EMT gene signature (Fig. 3B and C, and Supplementary Figs S8E and S9F), we measured E-cadherin expression by immunoblotting (Fig. 3I) and Fibronectin by IF (Supplementary Fig. S12B–D). After *VIM-AS1* was silenced, the activation of TGF-β signaling failed to repress E-cadherin expression in comparison to the respective control, and the TGF-β-induced Fibronectin and Vimentin expression was partially suppressed (Fig. 3H and I, and Supplementary Fig. S12). These findings suggest that *VIM-AS1* is implicated in the EMT response to TGF-β.

To address which *VIM-AS1* variant could impact TGF-β signaling, we cloned v.1 and v.2 cDNAs from MDA-MB-231 cells and stably transfected each variant independently (Supplementary Fig. S13A–D). Overexpression of pc*VIM-AS1* v.2 enhanced significantly TGF-β induction of the CAGA_12_-luciferase reporter, in comparison to control (pcCtrl) or to pc*VIM-AS1* v.1 (Fig. 3J and Supplementary Fig. S13E). Similarly, *VIM-AS1* v.2 gain-of-function increased the TGF-β-induced, but not the basal levels, of *SERPINE1* and *FN1* mRNA levels in the same cells, as well as Fibronectin, SERPINE1 (PAI1) and Vimentin protein expression (Fig. 3K and Supplementary Fig. S13F–I). Altogether, the above data suggest that *VIM-AS1* v.2 enhances TGF-β/SMAD signaling in a direct and robust manner.

### *VIM-AS1* v.2 enhances SMAD nuclear accumulation

**Figure 4.**
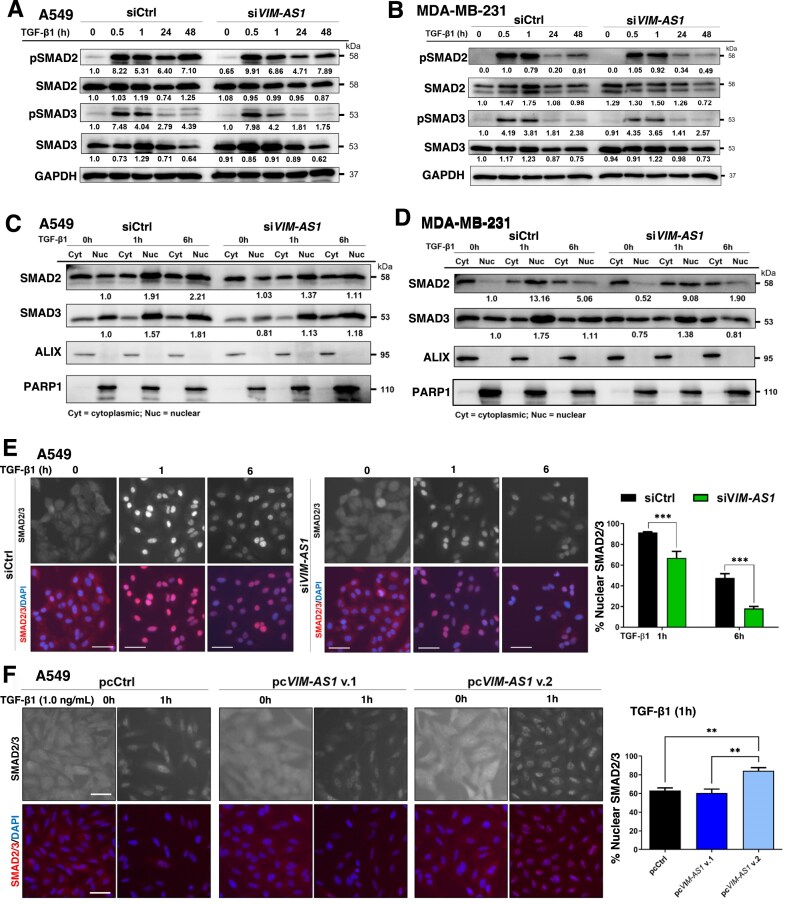
*VIM-AS1* v.2 enhances receptor-SMAD2/3 nuclear accumulation. (**A, B**) Protein expression levels of the indicated signaling proteins and GAPDH (as loading control) in total cellular extracts of A549 (A) and MDA-MB-231 (B) cells transiently transfected with siRNA control (siCtrl) or si*VIM-AS1* and stimulated or not with 5 ng/ml TGF-β1 for the indicated time periods. Densitometric values are normalized to unstimulated control. (**C, D**) Protein expression levels of SMAD2/3 in nuclear and cytoplasmic fractions of A549 (**C**) and MDA-MB-231 (**D**) cells transiently transfected with siRNA control (siCtrl) or si*VIM-AS1* and stimulated or not with 5 ng/ml TGF-β1 for the indicated periods. Corresponding immunoblot controls verify the relative purity of cell fractions based on the cytoplasmic (ALIX) and nuclear (PARP1) protein markers. Densitometric values of SMAD2 and SMAD3 nuclear fractions were normalized to unstimulated control. Representative immunoblots of three independent biological replicates along with molecular mass markers in kDa are shown in panels (A)–(D). (**E**) Representative IF microscopy pictures of A549 cells transiently transfected with siCtrl and si*VIM-AS1* and stimulated with vehicle (0 h) or 5 ng/ml TGF-β1 for 1 or 6 h. (**F**) Representative IF microscopy pictures of A549 cells transfected with empty vector (Ctrl) or pcDNA(pc)-*VIM-AS1* v.1 or pc*VIM-AS1* v.2 upon selection with neomycin and stimulated with vehicle (0 h) or 1 ng/ml TGF-β1 for 1 h. Values in panels (E) and (F) represent the percentage of SMAD2/3-stained nuclear intensity, normalized to the 0 h time point, and the SMAD2/3 proteins (black and white or red) and nuclei (blue; DAPI) are labeled. *P*-values in panel (E) are based on two-way ANOVA or in panel (F) on one-way ANOVA, followed by multiple paired comparisons conducted using Bonferroni’s post-test method. *P*-values: **P ≤.01; ***P ≤.001. Scale bars, 50 µm.

Since *VIM-AS1* silencing reduced TGF-β/SMAD signaling (Fig. 3), we examined its effect on TGFβRI-mediated C-terminal phosphorylation of SMAD2 (Ser465/467) and SMAD3 (Ser423/425). Our data showed no impact of *VIM-AS1* for the early C-terminal phosphorylation of SMAD2 and SMAD3 induced by TGF-β or the total level of these proteins in A549 and MDA-MB-231 cells (Fig. 4A and B), which is in line with the unperturbed expression of *TGFBR1* and *TGFBR2* (Supplementary Fig. S11A and C). To determine whether *VIM-AS1* influences receptor-SMAD protein stability, A549 cells with or without *VIM-AS1* knockdown were treated with CHX to inhibit protein synthesis for up to 8 h. Assessment of SMAD2/3 levels revealed that *VIM-AS1* knockdown did not alter SMAD2/3 stability (Supplementary Fig. S14A). Upon C-terminal phosphorylation, R-SMADs rapidly accumulate in the nucleus, which led us to evaluate whether *VIM-AS1* could affect the SMAD nucleocytoplasmic shuttling. Stimulation with TGF-β for 1 and 6 h, revealed robust accumulation of SMAD2 and SMAD3 in the nuclear fraction of A549 and MDA-MB-231 cells, which was decreased when *VIM-AS1* was silenced (Fig. 4C and D, and Supplementary Fig. S14B). Additionally, IF microscopy of SMAD2/3 in A549 and MDA-MB-231 cells after TGF-β stimulation showed a significant reduction in the percentage of SMAD2/3 localized in nuclei upon *VIM-AS1* knockdown (Fig. 4E and Supplementary Fig. S14C). Conversely, *VIM-AS1* v.2 overexpression led to a significant increase in the amount of nuclear SMAD2/3 compared to control cells or cells overexpressing *VIM-AS1* v.1 (Fig. 4F and Supplementary Fig. S14D). These results indicate that *VIM-AS1* v.2 facilitates nuclear accumulation of SMAD2/3, promoting TGF-β signaling. It is also worth observing that the impact of silencing *VIM-AS1* on gene responses to TGF-β (Fig. 3 and Supplementary Fig. S11) agrees with the impact that such silencing has on SMAD2/3 nuclear accumulation (Fig. 4).

### *VIM-AS1* interacts with the nucleoporin Nup358/RanBP2

**Figure 5.**
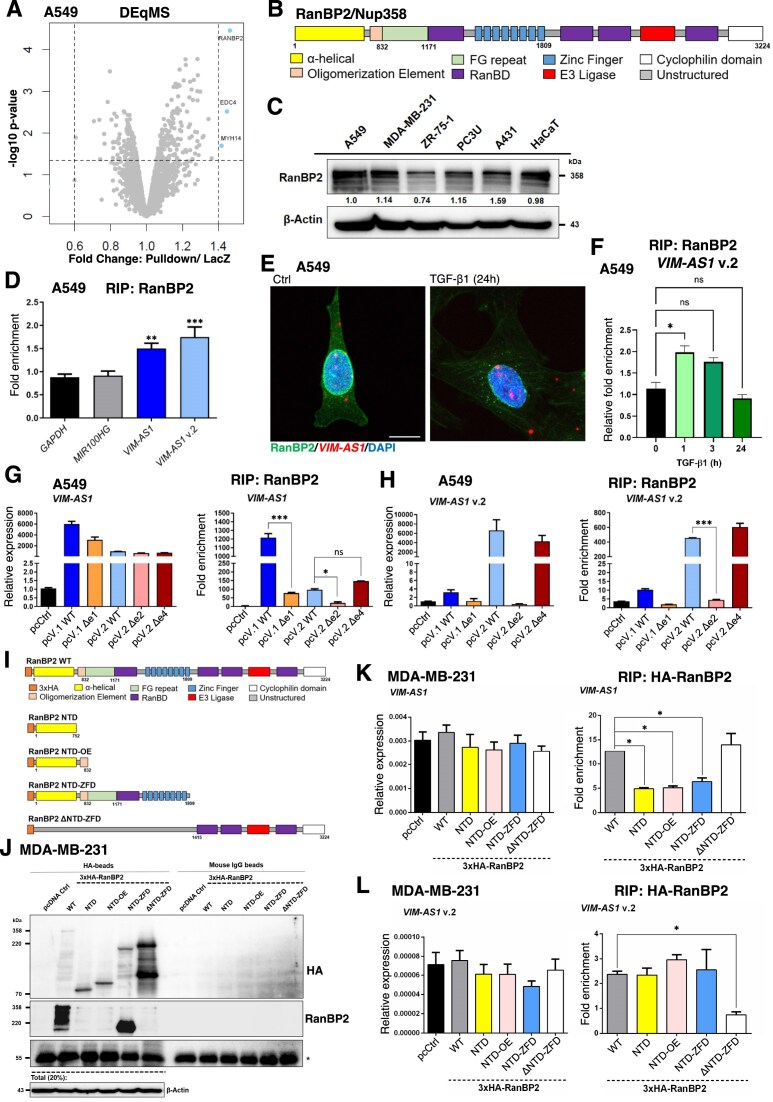
*VIM-AS1* interacts with the nucleoporin Nup358/RanBP2. (**A**) Volcano plot showing *VIM-AS1*-interacting proteins in A549 cells identified using ChOP-MS in two independent biological replicates. DEqMS v1.22.0 was employed to conduct the differential enrichment analysis. (**B**) Structure of RanBP2 with domains drawn as coloured boxes with residue numbers indicated. (**C**) RanBP2 protein expression in cellular extracts of the indicated cell lines. GAPDH was used as a loading control, and molecular mass (kDa) markers are indicated along with densitometric values normalized to A549. (**D**) RIP analysis using an antibody against RanBP2 in A549 cells. Fold-enrichment of the RanBP2-specific RIP relative to the IgG control is reported for *GAPDH, MIR100HG, VIM-AS1*, and *VIM-AS1* v.2. (**E**) RNA scope and IF staining, using fluorescent probes directed at *VIM-AS1* (red) and an anti-RanBP2 (green), with stained nuclei (blue; DAPI) in A549 cells. The images were captured at an original magnification of × 63. Scale bars: 10 μm. (**F**) RIP analysis using an anti-RanBP2 in A549 cells stimulated or not with TGFβ1 for 1, 3, and 24 h. Relative fold-enrichment of the RanBP2-specific RIP to the 0 h time point. (**G, H**) RT-qPCR, followed by RanBP2-specific RIP analysis of the *VIM-AS1* (G: v.1 and v.2; H: v.2) levels in A549 cells transiently transfected with empty vector (pcCtrl) or full-length *VIM-AS1* v.1 or v.2 in addition to the indicated mutated variants. In these experiments, the v.2 Δe2 deletion mutant cannot be detected by the set of primers used. RT-qPCR values represent fold-change of *VIM-AS1* expression normalized to *GAPDH*. RanBP2-specific RIP values relative to the IgG control represent fold-enrichment. (**I**) Schematic illustration of the domain boundaries of the 3 × HA-tagged RanBP2 variants. (**J**) 3 × HA-RanBP2 protein expression in cellular extracts of MDA-MB-231 transfected cells. Anti-HA immunoblot detects all proteins, whereas anti-RanBP2 immunoblot detects WT and NTD-ZFD proteins due to the epitope mapping in ZFD. β-Actin was used as a loading control. Representative immunoblots of two independent biological replicates along with molecular mass (kDa) markers indicated. A star indicates the IgG band recognized by the secondary antibody confirming immunoprecipitation in every sample. (**K, L**) RT-qPCR, followed by HA-specific RIP analysis of the *VIM-AS1* (K: v.1 and v.2; L: v.2) levels in MDA-MB-231 cells transiently transfected with full-length (WT) 3 × HA-RanBP2 in addition to the indicated deletion mutants. RT-qPCR values represent fold-change of *VIM-AS1* expression normalized to *GAPDH*, while HA-specific RanBP2 RIP values relative to the IgG control represent fold-enrichment. The data shown in panels (D), (F), (G), (H), (K), and (L) are presented as mean values of at least two biological replicates ± SEM, in technical triplicates. *P*-values in panel (D) are shown based on unpaired student’s *t*-test with Welch’s correction when comparing each fold-enrichment to the respective IgG control. *P*-values in panels (F)–(H), (K), and (L) are shown based on one-way ANOVA, followed by multiple paired comparisons conducted using Bonferroni’s post-test method. *P*-values: *P ≤.05; **P ≤.01; ***P ≤.001.

To identify RNA-binding proteins that could explain mechanistically how *VIM-AS1* facilitates SMAD2/3 nuclear accumulation, we performed a modified protocol of RNA ChOP, using biotinylated oligonucleotides complementary to *VIM-AS1* or *LacZ* (as a negative control) that were incubated with A549 cell extract, after crosslinking (Supplementary Fig. S15A). Unbiased isobaric labeling-based quantitative MS [54] was performed after the ChOP, showing different protein profiles pulled-down by *VIM-AS1* in comparison to the control *LacZ* (Supplementary Fig. S15B and Supplementary Table S7). Differential enrichment analysis using two distinct tools (i.e. DEqMS [43] and DEPs [44]) revealed four proteins differentially interacting with *VIM-AS1* and ranked with a top-score (RanBP2, EDC4, MYH14, and TAF7). The enhancer of mRNA de-capping protein 4 (EDC4) and the nucleoporin Nup358/RanBP2 were the only common proteins identified by both enrichment tools (Fig. 5A and Supplementary Fig. S15C).

Nup358/RanBP2 is the largest constituent of the cytoplasmic face of the NPC with 3224-residues composed of an N–terminal S–shaped α–helical solenoid, a coiled–coil oligomerization element, phenylalanine-glycine (FG) repeats, four Ran–binding and eight zinc finger domains, an E3 ligase domain, and a C–terminal prolyl–isomerase domain (Fig. 5B) [38]. For brevity, Nup358/RanBP2 will be further referred to as RanBP2. RanBP2 is expressed at roughly comparable levels by the cell types used in this study (Fig. 5C) and its subcellular localization at the perinuclear ring circumscribing the nuclear envelope towards the cytoplasm was confirmed by IF (Supplementary Fig. S15D–F). Furthermore, independent RIP assays followed by RT-qPCR confirmed that endogenous RanBP2 IP from A549 and MDA-MB-231 cells was significantly enriched with both *VIM-AS1* variants in comparison to control IgG, while no significant enrichment was observed for *GAPDH* mRNA or *MIR100HG* lncRNA (Fig. 5D and Supplementary Fig. S15G–J). Additionally, RNA-FISH and IF staining demonstrated the co-localization of *VIM-AS1* and RanBP2 in A549 cells (Fig. 5E). Interestingly, at an early time point (1 h) of TGF-β stimulation, a significant increase in the fold-enrichment of *VIM-AS1* v.2, but not v.1, within RanBP2 immunocomplexes was observed in A549 cells, an effect not observed at later time points (Fig. 5F and Supplementary Fig. S15K–M). Furthermore, the ectopic overexpression of *VIM-AS1* v.1 or v.2 in MDA-MB-231 cells significantly increased their enrichment in RanBP2 immunocomplexes (Supplementary Fig. S16A and B). RNA-FISH showed that ectopic overexpression of *VIM-AS1* v.1 or v.2 led to accumulation of these variants in the nuclear border (Supplementary Fig. S16C and D). Thus, once TGF-β signaling is activated, cells can direct one fraction of *VIM-AS1* v.2 towards RanBP2, which could be a possible mechanism to control SMAD nuclear accumulation, which will later induce *VIM-AS1* v.2 levels.

To better characterize the lncRNA-NPC association, we first mapped the interactions between endogenous RanBP2 with different exons of *VIM-AS1* v.1 and v.2. For this purpose, full-length (WT) *VIM-AS1* v.1 and v.2 or mutated variants (i.e. v.1 Δexon-1, v.2 Δexon-2 and v.2 Δexon-4) (Supplementary Fig. S17A, B) were overexpressed in A549 cells followed by RIP of the endogenous RanBP2. Our results indicate that molecular structures of exon-1 from v.1 and exon-2 from v.2 (Supplementary Fig. S17C, D) were crucial for the enrichment of *VIM-AS1* v.1 or v.2 in RanBP2 complexes (Fig. 5G and H). Additionally, to evaluate the binding capability of *VIM-AS1* to different domains of RanBP2, HA-tagged full-length RanBP2 or RanBP2 fragments, i.e. N terminal domain (NTD); NTD-oligomerization domain (NTD-OE); NTD-zinc finger domain (NTD-ZFD); and deletion of the NTD and ZFDs (ΔNTD-ZFD) (Fig. 5I), were overexpressed in MDA-MB-231 and A549 cells (Fig. 5J, and Supplementary Fig. S18A–C), followed by RIP using an anti-HA antibody. Overexpression of RanBP2 or RanBP2 fragments did not affect *VIM-AS1* expression in these cells. *VIM-AS1* v.1 bound preferentially the RanBP2 C-terminal domain (CTD), encompassing three Ran-binding, an E3 ligase and a cyclophilin domain, since the three N-terminal domain constructs showed reduced interaction; in contrast, *VIM-AS1* v.2 bound preferentially to the NTD region, since the absence of RanBP2 NTD reduced significantly the complex formation with v.2 (Fig. 5K and L, and Supplementary Fig. S18D and E). Furthermore, the interaction mapping of *VIM-AS1* with RanBP2 partially validated independent interaction predictions solely based on their respective primary sequences using a predictive algorithm (catRAPID omics v2.0, Tartaglia lab) [55]. Accordingly, a higher affinity score for *VIM-AS1* v.1 exon-1 binding to RanBP2 CTD was predicted, while the v.2 prediction suggested exons-2 and -3 as interacting with RanBP2 (Supplementary Fig. S18F and G). Beyond these predictions, the interaction of *VIM-AS1* v.2 with RanBP2 NTD in our experimental study is in line with previous observations of RNA-binding activity residing on this domain of RanBP2 [38]. Our data underscore that different exons among the two *VIM-AS1* variants can be the structural determinants that mediate their respective binding to distinct domains of RanBP2. Finally, using HDOCK [45] to generate docking simulations of *VIM-AS1* v.1 exon-1 and v.2 exons 1–3 bound to RanBP2 CTD or NTD, respectively, we found the most likely interaction among RanBP2 with *VIM-AS1* with a negative score of -274.06 for v.1 and RanBP2 CTD and −392.24 for v.2 and RanBP2 NTD, in addition to a confidence score proximal to 1.0 (Supplementary Fig. S18H and I). These simulations, although theoretical, helped to visualize how *VIM-AS1* could potentially bind to RanBP2 in order to promote SMAD nuclear accumulation, as explained below.

### SMAD nuclear accumulation relies on Nup358/RanBP2 and *VIM-AS1*

**Figure 6.**
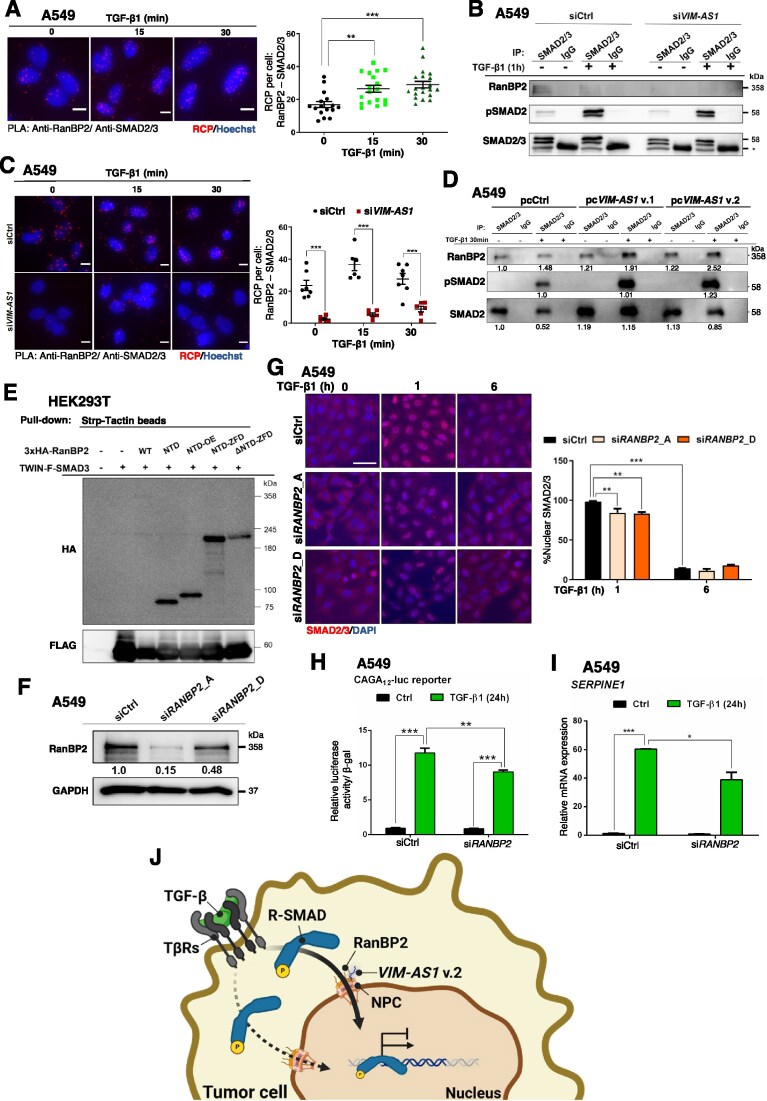
SMAD2/3 nuclear accumulation relies on RanBP2 and *VIM-AS1*. (**A**) PLA was used to show the co-localization of SMAD2/3 with RanBP2 in A549 cells incubated with vehicle (0 h) or 5 ng/ml TGF-β1 for 15 or 30 min. Nuclei are shown in blue (Hoechst), and PLA RCP in red. Quantification for the co-localization between SMAD2/3 with RanBP2 in A549 cells is shown. (**B**) Protein complex formation between endogenous SMAD2/3 with RanBP2. A549 cells transiently transfected with control siRNA (siCtrl) or si*VIM-AS1* and incubated with vehicle (−) or 5 ng/ml TGF-β1 for 1 h, and the total protein lysates were IP with a SMAD2/3 antibody or nonspecific IgG, followed by SDS–PAGE and immunoblotting with RanBP2, pSMAD2 (Ser465/467) and SMAD2/3 antibodies. (**C**) PLA was used to show the co-localization of SMAD2/3 with RanBP2 in A549 cells transiently transfected with siCtrl or si*VIM-AS1* and incubated or not with 5 ng/ml TGF-β1 for 15 or 30 min. Nuclei are shown in blue (Hoechst), and PLA RCP in red. Quantification for the co-localization between SMAD2/3 with RanBP2 in A549 transfected cells is shown. (**D**) Protein complex formation between endogenous SMAD2/3 with RanBP2. A549 cells transfected with empty vector (Ctrl) or pcDNA(pc)-*VIM-AS1* v.1 or pc*VIM-AS1* v.2 upon selection with neomycin, were stimulated or not with 5 ng/ml TGF-β1 for 30 min. The total protein lysates were IP with SMAD2/3-specific antibody or nonspecific IgG, followed by SDS–PAGE and immunoblotting with RanBP2, pSMAD2, and SMAD2 antibodies. (**E**) Protein complex formation between TWIN-FLAG(F)-SMAD3 with 3 × HA-tagged RanBP2 WT or fragments in HEK293T cells. The total protein lysates were pulled-down with Strep-Tactin beads, followed by SDS–PAGE and immunoblotting with HA and FLAG antibodies. (**F**) Protein expression levels of RanBP2 and GAPDH (as loading control) in A549 protein extracts of cells transiently transfected with the indicated siRNAs against *RANBP2*; densitometric values were normalized to the control siRNA (siCtrl). (**G**) Representative IF microscopy pictures of A549 cells transfected with the indicated control siRNAs (siCtrl) or siRNA against *RANBP2* and stimulated or not with 5 ng/ml TGF-β1 for 1 or 6 h. The quantification represents the percentage of SMAD2/3-stained nuclear intensity, normalized to the 0 h time point. Scale bars, 50 µm. (**H**) Relative luciferase activity generated in A549 cells transiently transfected with siCtrl or *RANBP2*_A by additional transfection of the TGF-β-inducible CAGA_12_-luc reporter, normalized to β-galactosidase activity generated by a co-transfected reporter, after stimulation of the cells or not with 5 ng/ml TGF-β1 for 24 h. (**I**) RT-qPCR analysis of *SERPINE1* mRNA levels in A549 cells transiently transfected with siCtrl or si*RANBP2*_A. The PLA data quantification in panels (A) and (C) are presented as mean values of individual micrographs from at least two biological replicates ± SEM and *P*-values are shown based on one-way (A) or two-way (C) ANOVA, followed by multiple paired comparisons conducted using Bonferroni’s post-test method. The data shown in panels (G)–(I) are presented as mean values of three biological replicates ± SEM, in technical triplicates and *P*-values are shown based on two-way ANOVA, followed by multiple paired comparisons conducted by means of Bonferroni’s post-test method. *P*-values: *P ≤.05; **P ≤.01; ***P ≤.001. Representative immunoblots of at least two independent biological replicates along with molecular mass markers in kDa. (**J**) Schematic illustration of the mechanism whereby *VIM-AS1* v.2 forms complexes with RanBP2 at the NPC, thus enhancing (thick arrow) the nuclear accumulation of SMADs that then transcriptionally regulate their target genes. SMAD import (dotted arrow) to the nucleus via alternative NPCs to which *VIM-AS1* is not bound is also illustrated. The schematic was generated using BioRender.com.

To determine whether SMAD2/3 nuclear accumulation rely on RanBP2 and *VIM-AS1*, we measured the formation of RanBP2–SMAD complexes. By immunoprecipitating SMAD2/3 in the absence of crosslinking from A549 cells stimulated or not with TGF-β for 30 min or 6 h, RanBP2 was found to interact with SMAD2/3 (Supplementary Fig. S19A). IF microscopy also indicated co-localization of RanBP2 with a fraction of the nuclear SMAD2/3 (Supplementary Fig. S19B and C). More convincingly, PLA for RanBP2 and SMAD2/3 in A549 and MDA-MB-231 cells detected RanBP2–SMAD2/3 complexes surrounding the nucleus or inside the nucleus of these cells, which were significantly increased upon TGF-β stimulation for 15 and 30 min (Fig. 6A and Supplementary Fig. S19D–G). Furthermore, when *VIM-AS1* was silenced, SMAD2/3 failed to interact with RanBP2, as shown by co-immunoprecipitation (Fig. 6B and Supplementary Fig. S20A–C) and PLA experiments (Fig. 6C and Supplementary Fig. S20D). Conversely, *VIM-AS1* v.2 overexpression in A549 cells enhanced the amount of co-immunoprecipitated complexes of SMAD2/3 with RanBP2 by 1.7-fold in comparison to the control (Fig. 6D and Supplementary Fig. S20E).

We also investigated which RanBP2 domain interacted with SMAD2/3. HA-RanBP2 WT or fragments, as described in Fig. 5I, were overexpressed in HEK293T and MDA-MB-231 cells (Supplementary Fig. S21A–C), followed by noncrosslinked co-immunoprecipitation of SMAD2/3. Even though the very large full-length (358 kDa) HA-RanBP2 WT could not be easily detected compared to the other RanBP2 domains, we observed HA-RanBP2 NTD associating with SMAD2/3 in both HEK293T and MDA-MB-231 cells (Supplementary Fig. S21D and E). Moreover, we prepared the cell extracts from HEK293T cells stimulated with TGF-β for 1 h and overexpressing a full-length TWIN-F-tagged SMAD3 in combination with either HA-RanBP2 WT or fragments (Fig. 5I). After noncrosslinked pull-down assay using Strep-Tactin beads, complexes detected between RanBP2 WT and domains with TWIN-F-SMAD3 supported our findings with endogenous SMAD2/3, indicating stronger co-immunoprecipitation with all fragments containing the NTD and weaker, yet detectable co-immunoprecipitation even with a RanBP2 fragment that lacked the NTD (Fig. 6E and Supplementary Fig. S21F). Furthermore, docking simulations of RanBP2 NTD-ZFD binding with SMAD2 and SMAD3 were generated with HDOCK [45], showing the most likely interaction among these proteins with a negative score of −320.20 for SMAD2 and −323.61 for SMAD3 (Supplementary Fig S21G and H).

We finally sought to validate our biochemical findings on RanBP2 and SMAD complexes functionally by silencing RanBP2 using four independent siRNAs (Fig. 6F and Supplementary Fig. S22A). Two of the most efficient siRNAs significantly impaired the long-term viability of A549 cells (Supplementary Fig. S22B), as expected for an NPC protein. The slightly lower viability of the cells did not prohibit us from performing signaling assays during the first few days after transfections. As predicted from the interaction data, silencing RanBP2 decreased significantly the amount of nuclear SMAD2/3 in A549 and MDA-MB-231 cells after TGF-β stimulation (Fig. 6G, and Supplementary Fig. S22C and D). Additionally, TGF-β-induced CAGA_12_-luciferase reporter activation was significantly, yet not dramatically, reduced upon RanBP2 depletion in comparison to the controls (Fig. 6H). These results were further validated by the finding of a significant reduction of the TGF-β-induced mRNA levels for *SERPINE1, SNAI2*, and *MMP2* in A549 and MDA-MB-231 cells upon RanBP2 silencing (Fig. 6I, and Supplementary Fig. S22E and F). Furthermore, TGF-β stimulation for 1 h or *VIM-AS1* silencing did not affect the nuclear localization of deleted in breast cancer 1 (DBC-1) [56], whose import to the nucleus requires RanBP2, while, as expected, the knockdown of RanBP2 impaired the nuclear accumulation of DBC-1 (Supplementary Fig. S22G and H). Collectively, our results suggest a stepwise mechanism, whereby *VIM-AS1* v.2 facilitates the formation of SMAD2/3-RanBP2 complexes, thus enhancing the nuclear accumulation of SMADs and transcriptional regulation of target genes (Fig. 6J).

### *VIM-AS1* impacts long-term pro-tumorigenic actions of TGF-β signaling

**Figure 7.**
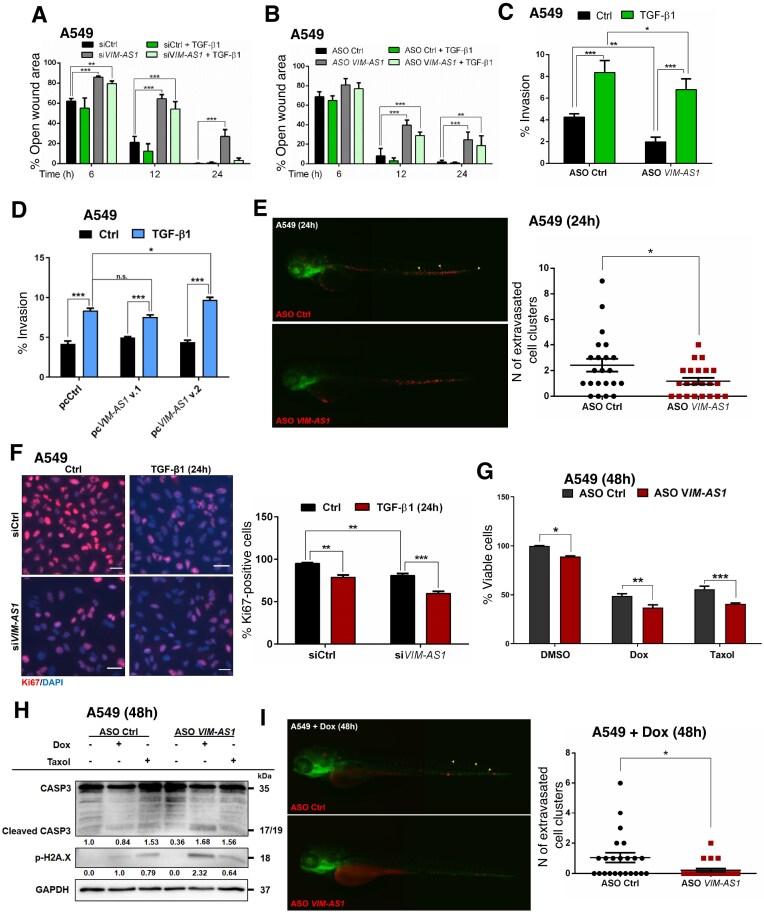
*VIM-AS1* impacts long-term pro-tumorigenic actions of TGF-β signaling. (**A, B**) Cell culture wound healing assay with A549 cells transiently transfected with the indicated siRNA (**A**) or ASO (**B**) against *VIM-AS1* stimulated or not with 5 ng/ml TGF-β1 for the indicated time periods. The data are presented as percent open wound area and plotted as mean values of three biological replicates ± SEM, in technical triplicates and *P*-values are shown based on two-way ANOVA, followed by multiple paired comparisons conducted by means of Bonferroni’s post-test method. (**C, D**) Matrigel invasion assay in trans-wells with A549 cells transiently transfected with control ASO (Ctrl) or ASO against *VIM-AS1* (**C**) or transfected with empty vector (Ctrl) or pcDNA(pc)-*VIM-AS1* v.1 or pc*VIM-AS1* v.2 upon selection with neomycin (**D**), followed by incubation or not with 5 ng/ml TGF-β1 for 18 h. The data represent the invaded cells as a percentage of the total cell number and are plotted as mean values of three biological replicates ± SEM, in technical triplicates. (**E**) *In vivo* extravasation and collagenous tail-fin invasion assay in zebrafish embryos injected with fluorescently labeled (red) A549 cells transiently transfected with ASO Ctrl or ASO *VIM-AS1* for 48 h prior to their microinjection in the duct of Cuvier of transgenic zebrafish with GFP-tagged (green) vasculature. Images were captured 24 h after microinjection. The data represent the extravasated cells that invaded the collagenous tail-fin as numbers of red fluorescent cell clusters and are plotted as mean values of at least 20 biological replicates (individual embryos) ± SEM and *P*-values are shown based on Wilcoxon matched-pairs test. (**F**) Representative IF microscopy pictures of A549 cells transiently transfected with control siRNA (siCtrl) or si*VIM-AS1* and stimulated or not with 5 ng/ml TGF-β1 for 24 h. The proliferation marker Ki67 (red) and nuclei (blue; DAPI) are labeled. Scale bars, 50 µm. Quantification of Ki67-positive cells expressed as percent of positive cells relative to the total number of cells under each condition, and plotted as mean values of three biological replicates ± SEM, each in technical duplicates and *P*-values are shown based on two-way ANOVA, followed by multiple paired comparisons conducted by means of Bonferroni’s post-test method. (**G**) Cell viability assay of A549 cells transiently transfected with ASO Ctrl or ASO *VIM-AS1*, followed by the presence or absence of DMSO (Ctrl), 0.5 µM Dox or 0.5 µM Taxol for 48 h. The data are normalized against the vehicle-treated Ctrl cells and presented as mean values of three biological replicates ± SEM, in technical triplicates, while *P*-values are shown based on two-way ANOVA, followed by multiple paired comparisons conducted by means of Bonferroni’s post-test method. (**H**) Representative immunoblot of cleaved Caspase-3 (CASP3) and p-H2A.X in A549 cells transiently transfected with ASO Ctrl or ASO *VIM-AS1* in the presence of DMSO or 0.5 µM Dox or Taxol for 48 h. GAPDH was used as a loading control, and molecular mass (kDa) markers are indicated along with densitometric values of normalized band intensity. (**I**) *In vivo* survival, extravasation and collagenous tail-fin invasion assay in zebrafish embryos injected with fluorescently labeled (red) A549 cells transiently transfected with ASO Ctrl or ASO *VIM-AS1* for 48 h prior to their microinjection in the duct of Cuvier of transgenic zebrafish with GFP-tagged (green) vasculature. Microinjected embryos were then incubated in water containing 5 µM Dox. Images were captured 48 h after microinjection. The data represent the extravasated cells that invaded the collagenous tail-fin as numbers of red fluorescent cell clusters and are plotted as mean values of at least 20 biological replicates (individual embryos) ± SEM and *P*-values are shown based on Wilcoxon matched-pairs test. *P*-values: *P ≤.05; **P ≤.01; ***P ≤.001.

We analyzed further the effects of *VIM-AS1* silencing on TGF-β-induced migration and invasion. Based on wound-healing assays, A549, H1299, and UT-SCC-12A cells transiently transfected with siRNA or ASO against *VIM-AS1*, stimulated or not with TGF-β, demonstrated significantly lower motility compared to their respective controls, a phenotype that was time-dependent and in agreement with the late onset of the pro-migratory response to TGF-β (Fig. 7A and B, and Supplementary Fig. S23A and B). Moreover, reducing *VIM-AS1* impaired significantly the TGF-β-induced invasion of multiple tumor cell types through Matrigel, in comparison to their respective controls (Fig. 7C, and Supplementary Fig. S23C and D). In line with our findings, *VIM-AS1* v.2 overexpression significantly increased the TGF-β-induced invasion of A549 and MDA-MB-231 cells, compared to pcCtrl (Fig. 7D and Supplementary Fig. S23E). Additionally, A549 cells that were transiently transfected with ASOs (control or *VIM-AS1*) and labeled with CM-Dil Dye (red) were assessed for *in vivo* invasiveness by injecting the cells in the duct of Cuvier of transgenic Tg(Fli1:EGFP) zebrafish embryos. The reduction in *VIM-AS1* significantly decreased the number of A549 cells that extravasated from the blood vessels and invaded the collagenous matrix of the tail (Fig. 7E).

Furthermore, as shown by the percentage of Ki67-positive cells, *VIM-AS1* silencing abrogated the proliferation of A549 and MDA-MB-231 cells, while the overexpression of either *VIM-AS1* v.1 or v.2 enhanced the proliferative potential of these cells (Fig. 7F and Supplementary Fig. S23F–I). Notably, *VIM-AS1* absence does not seem to affect TGF-β-suppressed proliferation. Similar results were obtained when cell viability was determined (Fig. 7G and Supplementary Fig. S23G–I). Thus, although the well-established antiproliferative action of TGF-β could be monitored in the examined cancer cell models, *VIM-AS1* appears to act more selectively and impair primarily pro-tumorigenic responses to TGF-β, such as EMT and cancer cell invasiveness.

Another cancer-related phenotype that is actually the opposite of the antiproliferative and tumor suppressing action of TGF-β, is resistance of cancer cells to chemotherapy, which is enhanced by TGF-β stimulation but also involves independent signaling mechanisms [1]. Thus, we investigated the effect of *VIM-AS1* on drug resistance in tumor cells treated with different concentrations of Dox or Taxol (Supplementary Fig. S24A and B). Silencing *VIM-AS1* led to a more potent response for Dox or Taxol in multiple cancer cells (Fig. 7G, and Supplementary Fig. S24C and D). On the other hand, overexpression of both *VIM-AS1* variants increased A549 and MDA-MB-231 resistance to Dox and Taxol (Supplementary Fig. S24E and F). In accordance with our cytotoxicity data, the levels of cleaved caspase-3, cleaved PARP1 (cell death markers) or phosphorylated pH2A.X (DNA damage marker) were enhanced in A549, MDA-MB-231 and H1299 cells after *VIM-AS1* was silenced (Fig. 7H, and Supplementary Fig. S24G and H). We also measured the mitochondrial potential, as a relevant event in the apoptotic pathway, in A549 and MDA-MB-231 cells. In line with the viability data, *VIM-AS1* knockdown alone significantly decreased the mitochondrial potential of tumor cells, which was further enhanced when combined with treatment with Dox or Taxol, suggesting a more potent induction of cell death when *VIM-AS1* was silenced (Supplementary Fig. S24I and J). Finally, A549 cells transiently transfected with ASOs (Ctrl or *VIM-AS1*) were injected in zebrafish embryos in the presence of 5 µM Dox for 48 h. A significant decrease in the number of extravasated A549 cell clusters after silencing of *VIM-AS1* was noted, suggesting that this lncRNA increased the proportion of drug-resistant cells circulating in the vasculature of the fish (Fig. 7I). Hence, induction of *VIM-AS1* expression enhances invasion, viability, and chemoresistance of cancer cells. However, these data, despite agreeing with previous studies on the role of *VIM-AS1* in EMT and invasiveness, should be considered as providing strong correlative suggestions and the absolute cause and effect relationships between *VIM-AS1* and the cancer phenotypes awaits deeper analysis based on knockout of *VIM-AS1* and rescue of the gene by specific variant transcripts.

## Discussion

We show that TGF-β induces *VIM-AS1* in a SMAD-dependent manner via cooperation with two additional TFs. Independent from its regulation by TGF-β signaling, *VIM-AS1* enhances the interaction of SMAD2/3 with Nup358/RanBP2 in the cytoplasmic face of the NPC, and also promotes SMAD nuclear accumulation, positively contributing to TGF-β signaling.

By analyzing the two major transcripts generated by the *VIM-AS1* gene, we found that TGF-β induced *VIM-AS1* v.2, and to a lesser extent v.1, in a large panel of normal, immortalized and cancer cell lines of diverse tissue types. Inspection of the *VIM-AS1* gene organization indicated that sequences inside the first intron of v.1 and 400 bp upstream from the TSS of v.2 are important, while ChIP-seq [36] and ChIP-PCR data indicate a rather broad binding area for SMAD3 that spans about 1000 bp upstream from the TSS. Analysis of the associated TFs revealed a new transcriptional regulatory mechanism that involves TGFβRI-mediated SMAD2/3 signaling and interaction of the phosphorylated SMADs with GATA6 and SPI1 in distinct complexes. The latter two TFs bind directly to the −400 bp TSS region, whereas the SMAD complex may bind directly or via the platform of associated TFs. This genomic module is conserved phylogenetically across mammals and possibly even in some birds and reptiles, all vertebrate classes that have active TGF-β/SMAD signaling [57]. It is worth noting that the v.1 TSS lies several kbp upstream from the GATA6-SPI1 binding sites, suggesting the possibility that additional TFs engage in the regulation of v.1. More proximal to the GATA6-SPI1 binding site and within the broad SMAD-binding area, lies the *VIM* TSS in the antisense orientation. Interestingly, neither GATA6 nor SPI1 silencing showed an effect on *VIM* expression, suggesting the possibility that each transcriptional unit in the *VIM* locus may utilize distinct factors for its expression. Relevant to this is the previously reported mechanism of regulation of *VIM* expression via a *VIM-AS1* v.1 RNA:*VIM* promoter DNA R loop that de-condenses locally nucleosomes and promotes binding of NF-κB that drives *VIM* expression in a colorectal cancer cell line [19]. Both the unique v.1 exon-1, that forms the R loop, and the binding sites for NF-κB, AP-1, and Sp1 (two more positive regulators of *VIM* expression) reside a few kbp upstream from the v.2 TSS. Our evidence suggests that the yet uncharacterized transcriptional mechanism that maintains expression of v.1 appears to lead to a relatively stronger (roughly 8- to 10-fold) constitutive expression of v.1 in the cell models tested here, relative to v.2. Yet the GATA6-SPI1 module under the instruction by SMADs, empowers v.2 with inducibility in response to TGF-β signals that raises the v.2 levels to almost half or equal to those of v.1, depending on the cell type. It will be interesting to investigate whether the GATA6-SPI1 module responds to additional SMAD family signals within the extended TGF-β family.

This study adds to the growing group of lncRNAs that regulate TGF-β signaling via cytoplasmic or nuclear mechanisms [17]. We now show that functional interference with *VIM-AS1* reduced the RanBP2–SMAD2/3 complexes and depleted significantly the nuclear pool of accumulated SMAD2/3. The Nup358/RanBP2 interaction with *VIM-AS1* is compatible with the previously reported ability of Nup358/RanBP2 to recognize RNA [38]. Nup358/RanBP2 carries several important functions ranging from the regulation of protein import to the nucleus, resolving protein complexes after their export to the cytoplasm, SUMOylating protein substrates based on its E3 SUMO ligase activity and even regulating kinetochore segregation during mitotic division [38, 58–60]. Nup358/RanBP2 extends from the cytoplasmic phase of the NPC, and it is interesting to hypothesize that the association of *VIM-AS1* with Nup358/RanBP2 may generate a more extended molecular surface that can link cytoplasmic components to the NPC. An important aspect to consider is molecular stoichiometry. It is highly unlikely that the mechanism by which *VIM-AS1* operates to facilitate SMAD shuttling relies on stoichiometric complexes of every RanBP2 protein paired with a corresponding *VIM-AS1* molecule. This is not supported by the RNAscope/IF analysis. Instead, we envision a case where some of the nuclear pores decorate their RanBP2 with *VIM-AS1* and these pores preferentially facilitate SMAD import. It will be most exciting to develop technology that can visualize our hypothetical model. This is important since the stoichiometric ratio of imported SMADs to the number of nuclear pores per cell remains also unknown. Furthermore, RanBP2 forms extended oligomers [38] and RNAs occupy much larger area or volume, relative to a large protein, suggesting that future structural approaches in resolving nucleo-protein complexes at the NPC will be most valuable.

Our evidence implicates both *VIM-AS1* v.1 and v.2 as Nup358/RanBP2 partners. However, the domain mapping analysis that coupled v.2 to the Nup358/RanBP2 NTD may appear more favorable since the RNA-binding domain of Nup358/RanBP2 was earlier mapped to the NTD [38]. A potential mechanism that may explain specificity of Nup358/RanBP2 interaction with *VIM-AS1*, can be the presence of RNA modifications such as N6-methyladenosine (m^6^A) or other modifications, an area that deserves future attention. Nup358/RanBP2 is a docking station for importin-β1 that depends on Ran activity [58]. Thus, SMAD2/3 interaction with the Nup358/RanBP2 could be linked to the SMAD-importin association and the Ran-mediated mechanism of SMAD protein translocation through the nuclear pore. Notwithstanding, our findings demand deeper structural and kinetic analysis in order to understand the more general contribution of *VIM-AS1* to the mechanism of SMAD2/3 nuclear accumulation. Furthermore, it is possible that *VIM-AS1* is implicated in the accumulation of other proteins into the nucleus, independent from TGF-β signaling [4, 6, 7], although we provide evidence for a single nuclear protein (DBC-1), whose residence in the nucleus is not perturbed by *VIM-AS1* silencing. Additionally, the cytoplasmic fraction of *VIM-AS1* may associate with EDC4, a key component of mRNA processing that facilitates decapping and recruits decay factors [61], thereby influencing the stability of TGF-β-induced transcripts.

Hence, *VIM-AS1* appears to join the function of the lncRNA *NORAD* in the above mechanism since *NORAD* contributes to the importin-β1 interaction with SMAD3 [5]. Finally, Nup358/RanBP2 has established functions as an end step in the cytoplasmic export of nuclear proteins by catalyzing the unloading of nuclear cargo from exportin-1/CRM1 and the “recycling” of Ran GTPase towards new cycles of import to the nucleus [59]. Since SMADs are exported to the cytoplasm [9–12], examining the role of Nup358/RanBP2 in the context of SMAD protein export to the cytoplasm and even on the documented modification of SMADs by SUMOylation [62], another functional feature of Nup358/RanBP2, is also warranted. The work on lncRNAs that associate with the nuclear import-export machinery suggests the possibility, indirectly supported by studies on the mechanochemical flexibility and adaptability of NPCs [15], that dedicated NPC pools exist that mediate transport of specific signaling mediators, such as the SMADs.

The importance of the above mechanism that facilitates SMAD nuclear accumulation and consequently positively contributes to TGF-β signaling, reflects one aspect of the multifaceted regulation of TGF-β signaling during cancer progression [1, 3]. As discussed above, independent functions of *VIM-AS1* have been reported in cancer cells, ranging from ceRNA function to regulation of mRNA stability and DNA-based transcriptional control [19–22, 63, 64]. A recent report implicating *VIM-AS1* in TGF-β-induced tissue fibrosis [65], suggests that our findings may have broader significance in human diseases beyond cancer, where TGF-β signaling plays key roles, such as fibrotic and chronic inflammatory conditions. Thus, the detailed analysis of *VIM-AS1* biochemistry and cellular function opens a wide window for deeper understanding of fundamental cell biological processes that impact the flow of molecular signaling in both normal and cancer cells. The case exemplified here for SMAD shuttling via the NPC suggests that other transport events may depend on the cooperation between multiprotein complexes and noncoding RNAs.

## Supplementary Material

gkaf1526_Supplemental_Files

## Data Availability

All primary RNA-sequencing data have been deposited to GEO (accession number GSE281521; https://www.ncbi.nlm.nih.gov/geo/). The proteomics data are deposited in to PRIDE (https://www.ebi.ac.uk/pride/) under Accession Number PXD057186. The additional experimental resources used and/or analyzed during the current study are available from the corresponding author on reasonable request.
