# Supplementary material for: The long noncoding RNA VIM-AS1 and nucleoporin Nup358/RanBP2 regulate SMAD nuclear accumulation during TGF-β signaling
Source: Nucleic Acids Res. 2026 Jan 20;54(2):gkaf1526. doi: 10.1093/nar/gkaf1526 (PMC12817083; doi:10.1093/nar/gkaf1526)
Supplement: gkaf1526_Supplemental_Files [file gkaf1526_supplemental_files.zip › RodriguesJunior_VIM-AS1_Nov 2025_NAR_suppl_file_clean_subm.pdf]

**The long non-coding RNA *VIM-AS1* and nucleoporin Nup358/RanBP2 regulate SMAD nuclear accumulation during TGF-β signaling**

Dorival Mendes Rodrigues-Junior, Mohamad Moustafa Ali, Yuka Itoh, Mafalda Sousa Ferreira, Johan Heldin, Hao Fu, André Hoelz, Carl-Henrik Heldin, Aristidis Moustakas

## Supplementary Information

**Supplementary Table S1.** Primer sequences used for qPCR assays.

| Gene Symbol | Forward (F) and Reverse (R) Primers Sequences (5'- 3') |
|---|---|
| **RT-qPCR** | |
| ***BMPR2*** | F - ATGCAGCCATAAGCGAGGTT<br>R - CCCCTGGGAAGAGGTCTGTA |
| ***CASC2*** | F - TTCAGACACACACCACACCTC<br>R - CGATTTCTGGGCCTCACACTA |
| ***DSCAM-AS1*** | F - GATCCTTGTTTGGTCTCACTCC<br>R - ATGCCTATGTGGGTGATTGG |
| ***FN1*** | F - CCCAGACTTATGGTGGCAATTC<br>R - AATTTCCGCCTCGAGTCTGA |
| ***GATA6*** | F - ACGAACAGGGCGATTTCCTT<br>R - GTTGGGGGTAACGTCTGCAA |
| ***GAPDH*** | F - GGAGTCAACGGATTTGGTCGTA<br>R - GGCAACAATATCCACTTTACCA |
| ***HPRT1*** | F - CCCTGGCGTCGTGATTAGT<br>R - CACCCTTTCCAAATCCTCAGC |
| ***KCNIP4IT1*** | F - TAACCCCTCTCCTCTCCTCC<br>R - GCTGTGGTAAAGGGAGGAGT |
| ***MALAT1*** | F - GTGAGCAAACTGTGTTGGCGTG<br>R - CATCGAGGTGAGGGGTGAAGGG |
| ***MIR100HG*** | F - AACTTGGCTTCCTCGCTTCT<br>R - CTTTGTCCTTGCACTGGGGA |
| ***MMP2*** | F - AGATGCCTGGAATGCCAT<br>R - GGTTCTCCAGCTTCAGGTAAT |
| ***MMP10*** | F - CCCCTGGTGCCCACAAA<br>R - TCACACTTGGCTGGCATCTC |
| ***RANBP2 (CTD)*** | F - CAATGGAAATGGGGAGGACTTT<br>R - CATCACTTCAGTCCCACCTGTA |
| ***RANBP2 (ZFD)*** | F - CTCCCAAAACACCACCTCGA<br>R - CTGAAGGGCCAGTAGTTGCA |
| ***SERPINE1*** | F - GAGACAGGCAGCTCGGATTC<br>R - CTCAGATTTGACCTGTCTGCAAA |
| ***SNAI2*** | F - AGACCCTGGTTGCTTCAAGGA<br>R - CAGGGATACCCTTTAGCAGTTTT |
| ***SOX2-OT*** | F - AACACCCTGATCTGGCATGG<br>R - ATATGGCTGTTGCCTGGCTT |
| ***VIM*** | F - AGTTTCGTTGATAACCTGTCC |

| | |
|---|---|
| | R - CTCTTCCAAACTTTTCCTCCC |
| ***VIM-AS1* v.1 v.2 (P1)** | F - CCATGTGTGCGATTCACAAGCCTT<br>R - TGATGCTGATGCTACAGGTCTGAGTA |
| ***VIM-AS1* v.1 (P2)** | F - GAGAAGAGGCGAACGAGGG<br>R - CCAAGGCTTGTGAATCGCA |
| ***VIM-AS1* v.2 (P3)** | F - AGATGTCTGCAAGGGGAACA<br>R - TTCTGATCACCACGAGTCCA |
| | |
| **ChIP-qPCR** | |
| ***VIM-AS1* _R1** | F - GCTGGAGGGAGAGGGGAA<br>R -GGACAGAGGAGGAAATGCGA |
| ***VIM-AS1* _R2** | F - TTTGTGTGCAGTTGAAGGCC<br>R - CTTCCCCTCCTTCCTTCTCC |
| ***VIM-AS1* _R3** | F - CCGAAGTCCCGCTGAAAC<br>R - CCACACCCAAACACCACG |
| ***SERPINE1*** | F - GCAGGACATCCGGGAGAGA<br>R - CCAATAGCCTTGGCCTGAGA |

**Supplementary Table S2.** List of antibodies used with dilution factors and application.

| Antibody | Application | Manufacturer and Cat. # |
|---|---|---|
| Anti-ALIX | Immunoblotting (1:250)<br>Immunoprecipitation 2.0 μg | SantaCruz Biotechnology #sc-53540 |
| Anti-α-Tubulin | Immunoblotting (1:1,000) | Merck/Millipore #T9026 |
| Anti-β-Actin | Immunoblotting (1:1,000) | SantaCruz Biotechnology #sc-69879 |
| Anti-E-Cadherin (CDH1) | Immunoblotting (1:1,000) | Cell Signaling #3195S |
| Anti-Caspase3 (CASP3) | Immunoblotting (1:500) | Cell Signaling #9662 |
| Anti-DBC-1 (EPR19747) | Immunofluorescence (1:150) | Abcam #ab215852 |
| Anti-Fibronectin (FN1) | Immunoblotting (1:1,000)<br>Immunofluorescence (1:150) | Merck/Millipore #F3648 |
| Anti-Flag (DYKDDDDK) Tag | Immunoblotting (1:1,000) | ThermoFisher Scientific #MA1-91878 |
| Anti-GAPDH | Immunoblotting (1:1,000) | ThermoFisher Scientific #AM4300 |
| Anti-GATA6 | Immunoblotting (1:1,000)<br>Immunofluorescence (1:150)<br>PLA (1:150) | Cell Signaling #5851 |
| Anti-HA-Tag (C29F4) | Immunoblotting (1:500) | Cell Signaling #3724 |
| Anti-p-H2A.X - Ser139 | Immunoblotting (1:1,000) | Cell Signaling #9718 |
| Anti-Ki67 | Immunofluorescence (1:500) | Abcam #ab15580 |
| Anti-PAI1 | Immunoblotting (1:1,000) | BD Biosciences #612025 |
| Anti-PARP1 | Immunoblotting (1:1000) | Cell Signaling #9542 |
| Anti-Cleaved PARP1 | Immunoblotting (1:1000) | Cell Signaling #5625 |
| Anti-RanBP2/Nup358 | Immunoblotting (1:500)<br>Immunofluorescence (1:150)<br>PLA (1:150) - RIP 2.5 μg | Abcam #ab64276 |
| Anti-SPI1 | Immunoblotting (1:250)<br>Immunofluorescence (1:100)<br>PLA (1:100) | SantaCruz Biotechnology #sc-352 |
| Anti-SMAD2 | Immunoblotting (1:1,000) | Cell Signaling #5339S |
| Anti-SMAD3 | Immunoblotting (1:1,000) | Cell Signaling #9523S |
| Anti-SMAD2/3 | Immunoblotting (1:1,000)<br>Immunofluorescence (1:200)<br>PLA (1:200) | BD Biosciences #610843 |
| Anti-SMAD4 | Immunoblotting (1:500) | Cell Signaling #38454S |

| | | |
|---|---|---|
| Anti-Phospho-SMAD3 (pSMAD3; Ser423/425) | Immunoblotting (1:1,000) | Cell Signaling #9520S |
| Anti-Phospho-SMAD2 (pSMAD2; Ser465/467) | Immunoblotting (1:1,000) | Merck/Millipore #AB3849 |
| Anti-Vimentin (VIM) | Immunoblotting (1:1,000)<br>Immunofluorescence (1:150) | Cell Signaling #5741 |
| Mouse control IgG | Immunoprecipitation 2.0 µg<br>RIP 2.5 µg | Abcam #ab184113 |

**Supplementary Table S3.** List of siRNAs and ASOs.

| **siRNA** | **Product ID** |
|---|---|
| SMARTpool: ON-TARGETplus *SMAD2* | Dharmacon; L-003561-00-0005 |
| SMARTpool: ON-TARGETplus *SMAD3* | Dharmacon; L-020067-00-0005 |
| ON-TARGETplus non-targeting pool | Dharmacon; D-001810-10-20 |
| FlexiTube GeneSolution Hs_LOC100507347_2 for *VIM-AS1* (A) | Qiagen; SI05721590 |
| FlexiTube GeneSolution Hs_LOC100507347_4 for *VIM-AS1* (D) | Qiagen; SI05721604 |
| FlexiTube GeneSolution Hs_SPI1_6 for *SPI1* (E) | Qiagen; SI05118344 |
| FlexiTube GeneSolution Hs_SPI1_7 for *SPI1* (F) | Qiagen; SI05118351 |
| FlexiTube GeneSolution Hs_GATA6_7 for *GATA6* (B) | Qiagen; SI04301220 |
| FlexiTube GeneSolution Hs_GATA6_6 for *GATA6* (C) | Qiagen; SI04204396 |
| FlexiTube GeneSolution Hs_RANBP2_2 for *RANBP2* (A) | Qiagen; SI00698250 |
| FlexiTube GeneSolution Hs_RANBP2_4 for *RANBP2* (B) | Qiagen; SI00698264 |
| FlexiTube GeneSolution Hs_RANBP2_5 for *RANBP2* (C) | Qiagen; SI03043901 |
| FlexiTube GeneSolution Hs_RANBP2_6 for *RANBP2* (D) | Qiagen; SI03117310 |
| Negative Control siRNA | Qiagen; 1027417 |
| Antisense LNATM GapmeR Standard for *VIM-AS1* (#01) | Qiagen; 339511 LG00792233-DDA |
| Antisense LNATM GapmeR Standard for *VIM-AS1* (#02) | Qiagen; 339511 LG00792234-DDA |
| Antisense LNATM GapmeR Control (5) | Qiagen; 339515 LG00000002-DDA |

**Supplementary Table S4.** Primer sequences used for *VIM-AS1* cloning.

| **Primers** | **Sequences (5'- 3')** |
|---|---|
| KpnI-***VIM-AS1***-v1-F | TTTGGTACCTTCTCCCGGAGGCGCATGATGTCC |
| XhoI-***VIM-AS1***-R | TTTCTCGAGTAGCATATCCAAAAATGGTATTGC |
| KpnI-***VIM-AS1***-v2-F | TTTGGTACCCTCCTGGAATGGGCACTGGTCC |
| KpnI-***VIM-AS1***-v1-dEx1-F | TTTGGTACCACAGCAAAGCTCCCTTTGGATGACATAG |
| XhoI-***VIM-AS1***-v2-dEx4-R | TTTCTCGAGCCAGAGACAAACAAGTTGGCCATGG |

**Supplementary Table S5.** Excel RNA-sequencing file (associated separate file).

**Supplementary Table S6.** Biotinylated oligonucleotides used in the ChOP pull-down.

| ***VIM-AS1* Probes** | **LacZ Probes** |
|---|---|
| TATGTCATCCAAAGGGAGCT-3' Biotin | TCACGACGTTGTAAAACGAC-3' Biotin |
| GGCTTCTTTTATTCTGATCA-3' Biotin | ATTAAGTTGGGTAACGCCAG-3' Biotin |
| CCTGATGGATCCTTCCAAAA-3' Biotin | AGGTTACGTTGGTGTAGATG-3' Biotin |
| GCCACAGACAGGAACAATTC-3' Biotin | |
| CTATCTATTCTTCATTTCCT-3' Biotin | |

**Supplementary Table S7.** Excel primary protein data file from the ChOP analysis (associated separate file).

## Supplementary figures with legends

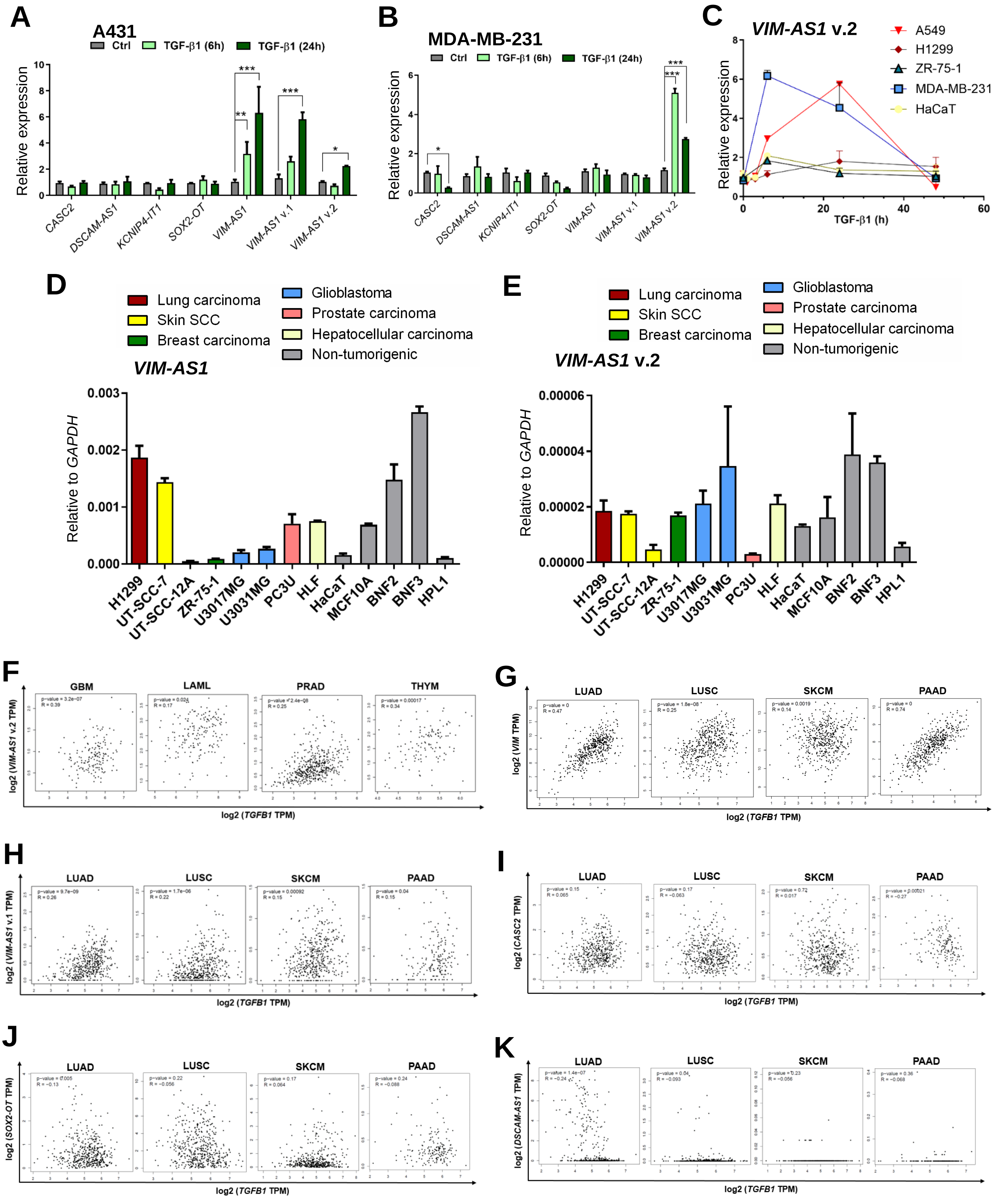

A
A431
Ctrl
TGF-β1 (6h)
TGF-β1 (24h)
Relative expression
CASC2
DSCAM-AS1
KCNIP4-IT1
SOX2-OT
VIM-AS1
VIM-AS1 v.1
VIM-AS1 v.2
B
MDA-MB-231
C
VIM-AS1 v.2
A549
H1299
ZR-75-1
MDA-MB-231
HaCaT
TGF-β1 (h)
D
Lung carcinoma
Skin SCC
Breast carcinoma
Glioblastoma
Prostate carcinoma
Hepatocellular carcinoma
Non-tumorigenic
VIM-AS1
Relative to GAPDH
H1299
UT-SCC-7
UT-SCC-12A
ZR-75-1
U3017MG
U3031MG
PC3U
HLF
HaCaT
MCF10A
BNF2
BNF3
HPL1
E
VIM-AS1 v.2
F
GBM
LAML
PRAD
THYM
log2 (VIM-AS1 v.2 TPM)
log2 (TGFB1 TPM)
G
LUAD
LUSC
SKCM
PAAD
log2 (VIM TPM)
H
log2 (VIM-AS1 v.1 TPM)
I
log2 (CASC2 TPM)
J
log2 (SOX2-OT TPM)
K
log2 (DSCAM-AS1 TPM)


Suppl. Fig S1

**Supplementary Figure S1. Related to Fig. 1.** TGF-β induces *VIM-AS1* v.2 expression. (**A, B**) RT-qPCR analysis of the indicated lncRNA levels in A431 (A) and MDA-MB-231 (B) cells upon stimulation with 5 ng/mL TGF-β1 for 6 and 24 h. Values represent fold-change of RNA expression normalized to *GAPDH* and expressed relative to the level at 0 h TGF-β1 (Ctrl). (**C**) RT-qPCR analysis of *VIM-AS1* v.2 levels in A549, H1299, ZR-75-1, MDA-MB-231 and HaCaT cells upon stimulation with 5 ng/mL TGF-β1 for 3, 6, 24 and 48 h. The data in A-C are normalized to each respective gene and expressed relative to the unstimulated control level (Ctrl) and presented as mean values of three biological replicates ± SEM, in technical triplicates. P-values are shown based on two-way ANOVA, followed by multiple paired comparisons conducted using Bonferroni's post-test method: *p ≤ 0.05; **p ≤ 0.01; ***p ≤ 0.001. (**D, E**) RT-qPCR analysis of the *VIM-AS1* v.1 and v.2 (D) and *VIM-AS1* v.2 (E) levels in the indicated cell models. Values represent fold-change of lncRNA expression normalized to *GAPDH* and presented as mean values of three biological replicates ± SEM, in technical triplicates. (**F**) Pearson correlation analysis of *VIM-AS1* v.2 expression in GBM, LAML, PRAD and THYM relative to the *TGFB1* expression, measured as transcripts per million (TPM) transformed by $\log_2$; data was obtained from TCGA. (**G-K**) Pearson correlation analysis of *VIM* (G), *VIM-AS1* v.1 (H), *CASC2* (I), *SOX2-OT* (J) and *DSCAM-AS1* (K) expression in LUAD, LUSC, SKCM and PAAD relative to *TGFB1* expression, measured as transcripts per million (TPM) transformed by $\log_2$; data was obtained from TCGA. P- and R-values are listed.

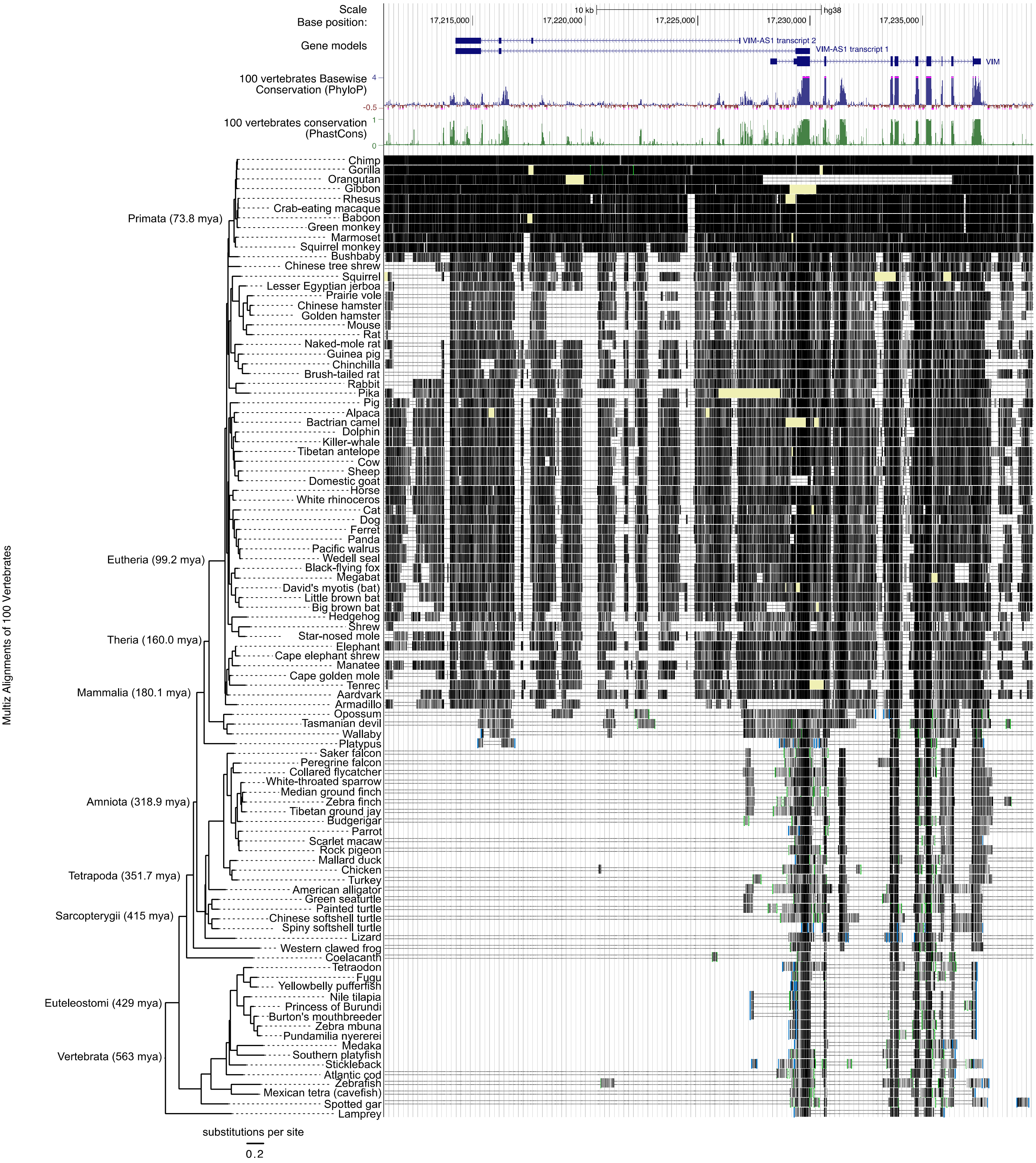

Scale
10 kb
hg38
Base position:
17,215,000
17,220,000
17,225,000
17,230,000
17,235,000
Gene models
VIM-AS1 transcript 2
VIM-AS1 transcript 1
VIM
100 vertebrates Basewise Conservation (PhyloP)
100 vertebrates conservation (PhastCons)
Multiz Alignments of 100 Vertebrates
Primata (73.8 mya)
Eutheria (99.2 mya)
Theria (160.0 mya)
Mammalia (180.1 mya)
Amniota (318.9 mya)
Tetrapoda (351.7 mya)
Sarcopterygii (415 mya)
Euteleostomi (429 mya)
Vertebrata (563 mya)
Chimp
Gorilla
Orangutan
Gibbon
Rhesus
Crab-eating macaque
Baboon
Green monkey
Marmoset
Squirrel monkey
Bushbaby
Chinese tree shrew
Squirrel
Lesser Egyptian jerboa
Prairie vole
Chinese hamster
Golden hamster
Mouse
Rat
Naked-mole rat
Guinea pig
Chinchilla
Brush-tailed rat
Rabbit
Pika
Pig
Alpaca
Bactrian camel
Dolphin
Killer-whale
Tibetan antelope
Cow
Sheep
Domestic goat
Horse
White rhinoceros
Cat
Dog
Ferret
Panda
Pacific walrus
Wedell seal
Black-flying fox
Megabat
David's myotis (bat)
Little brown bat
Big brown bat
Hedgehog
Shrew
Star-nosed mole
Elephant
Cape elephant shrew
Manatee
Cape golden mole
Tenrec
Aardvark
Armadillo
Opossum
Tasmanian devil
Wallaby
Platypus
Saker falcon
Peregrine falcon
Collared flycatcher
White-throated sparrow
Median ground finch
Zebra finch
Tibetan ground jay
Budgerigar
Parrot
Scarlet macaw
Rock pigeon
Mallard duck
Chicken
Turkey
American alligator
Green seaturtle
Painted turtle
Chinese softshell turtle
Spiny softshell turtle
Lizard
Western clawed frog
Coelacanth
Tetraodon
Fugu
Yellowbelly pufferfish
Nile tilapia
Princess of Burundi
Burton's mouthbreeder
Zebra mbuna
Pundamilia nyererei
Medaka
Southern platyfish
Stickleback
Atlantic cod
Zebrafish
Mexican tetra (cavefish)
Spotted gar
Lamprey
substitutions per site
0.2


**Suppl. Fig S2**

**Supplementary Figure S2. Related to Fig 1.** Conservation of *VIM* and *VIM-AS1* v.1 and v.2 transcripts during vertebrate evolution. Alignment of 100 vertebrate genomes to the human genome (hg38) produced with Multiz and respective conservation scores (PhyloP and PhastCons) were extracted from the Genome Browser (access March 18, 2024). Positive PhyloP scores in blue represent sites predicted to be conserved in the alignment, while negative red scores represent sites under fast evolution (scores are represented as -log p-values under a null hypothesis of neutral evolution). PhastCons scores represent the probability of a site being under negative selection and they range between 0 (less conserved) and 1 (more conserved). In the bottom alignment panel, pairwise alignments of each species to the human genome are displayed as a grayscale density plot that indicates alignment quality; single horizontal lines represent alignment regions with no bases in the aligned species; double single horizontal lines represent regions where the aligned species has one or more unalignable bases; pale yellow coloring represents alignments where the aligning species has Ns (missing data) in the gap region; vertical blue bars represent discontinuities in the alignment that persist indefinitely on either side; green vertical bars represent a short alignment to a different part of the aligned species genome. A phylogenetic tree relating the 100 vertebrates used to generate the alignment with Multiz is represented to the left of the alignment and was generated by PhyloFit. Divergence times for nodes of interest were obtained from TimeTree (accessed March 18, 2024).

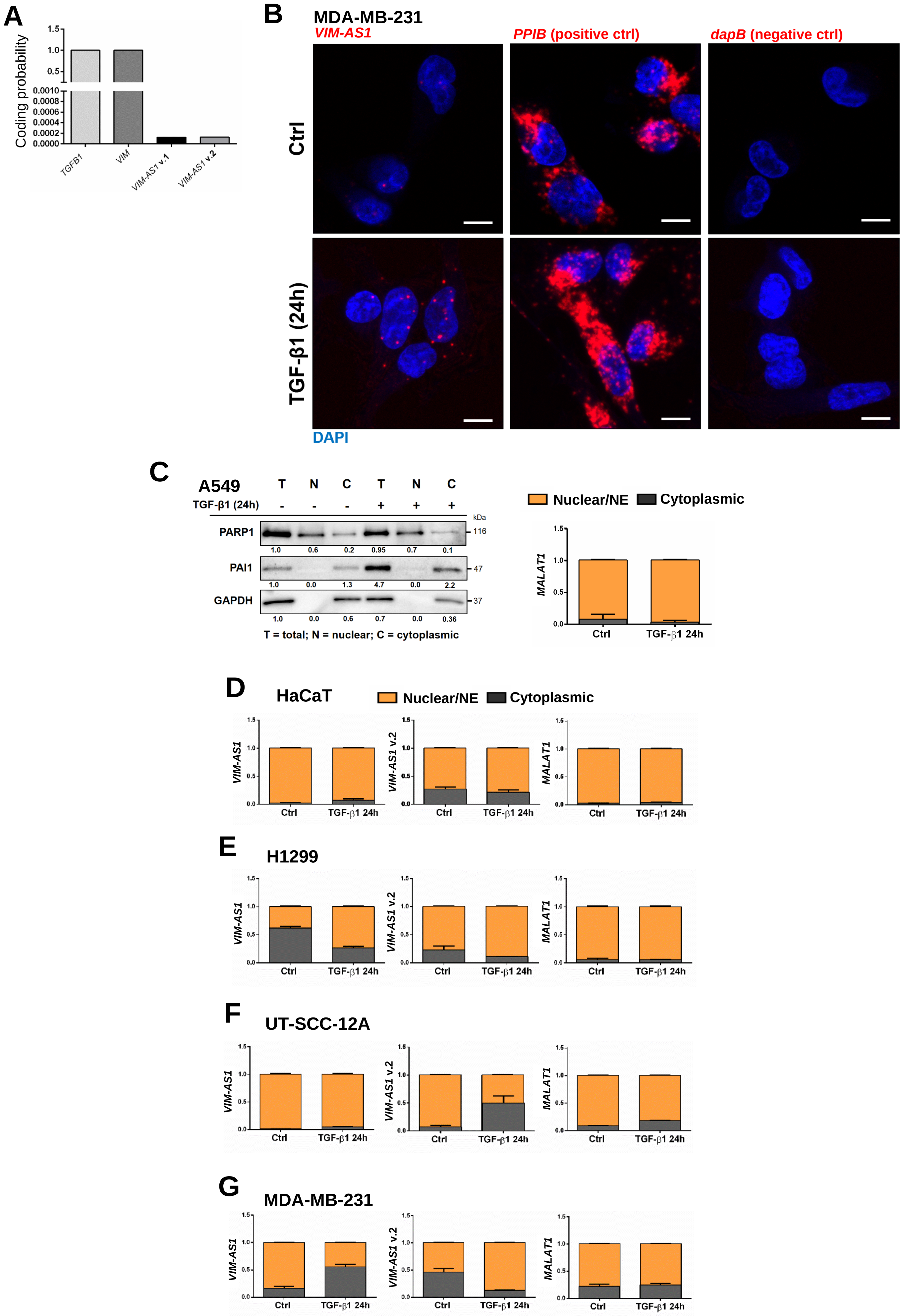
A
Coding probability
TGFB1
VIM
VIM-AS1 v.1
VIM-AS1 v.2
B
MDA-MB-231
VIM-AS1
PPIB (positive ctrl)
dapB (negative ctrl)
Ctrl
TGF-β1 (24h)
DAPI
C
A549
T N C T N C
TGF-β1 (24h) - - - + + +
kDa
PARP1
1.0 0.6 0.2 0.95 0.7 0.1
116
PAI1
1.0 0.0 1.3 4.7 0.0 2.2
47
GAPDH
1.0 0.0 0.6 0.7 0.0 0.36
37
T = total; N = nuclear; C = cytoplasmic
Nuclear/NE
Cytoplasmic
MALAT1
Ctrl
TGF-β1 24h
D
HaCaT
Nuclear/NE
Cytoplasmic
VIM-AS1
VIM-AS1 v.2
MALAT1
E
H1299
F
UT-SCC-12A
G
MDA-MB-231

Suppl. Fig S3

**Supplementary Figure S3. Related to Fig 1.** Subcellular distribution of *VIM-AS1* v.1 and v.2. (**A**) The CPAT software was used to predict the coding probability of protein-coding mRNAs (*TGFB1* and *VIM*) and the lncRNAs *VIM-AS1* v.1 and v.2. (**B**) RNAscope for *VIM-AS1*, *PPIB* (positive control) and *dapB* (negative control) in MDA-MB-231 cells stimulated or not with 5 ng/mL TGF-β1 for 24 h. Scale bar, 25 µm. (**C**) Representative corresponding immunoblots controls to verify the relative purity of A549 cellular fractions based on the nuclear (PARP1) and two cytoplasmic (PAI1 and GAPDH) protein markers. Molecular mass (kDa) markers are indicated. Expression levels of *MALAT1* lncRNA, in nuclear envelope (NE - orange) and cytoplasmic (grey) fractions of A549 lysates upon stimulation with 5 ng/mL TGF-β1 for 24 h. (**D-G**) Expression levels of *VIM-AS1* (v.1 and v.2), *VIM-AS1* v.2 and *MALAT1* lncRNAs, in nuclear/NE and cytoplasmic fractions of HaCaT (D), H1299 (E), UT-SCC-12A (F) and MDA-MB-231 (G) lysates upon stimulation with 5 ng/mL TGF-β1 for 24 h. Gene expression was normalized relative to the housekeeping gene *GAPDH* from the input. Data are presented as mean values of three biological replicates ± SEM, in technical triplicates.

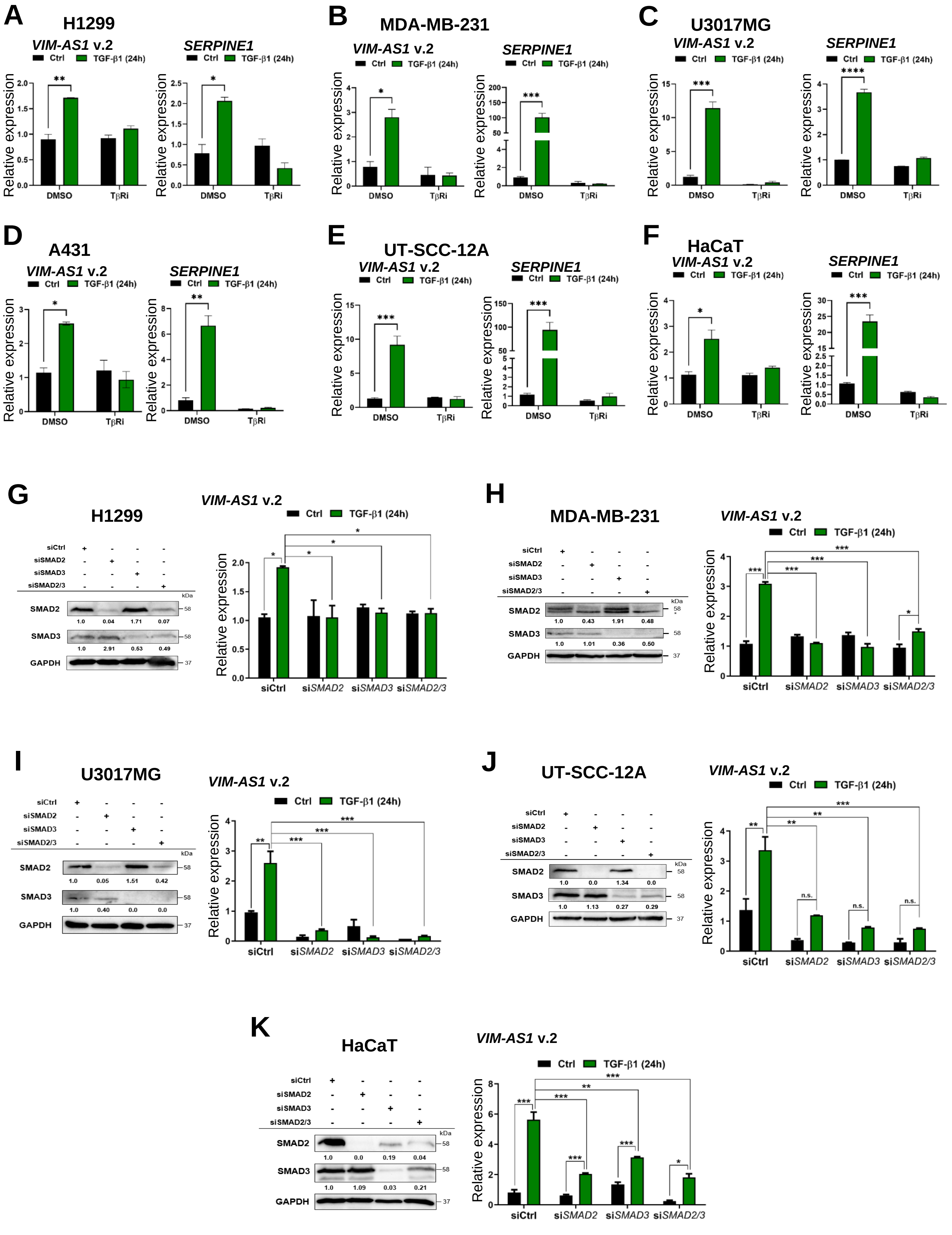

A
H1299
VIM-AS1 v.2
SERPINE1
Ctrl
TGF-β1 (24h)
Relative expression
DMSO
TβRi
B
MDA-MB-231
C
U3017MG
D
A431
E
UT-SCC-12A
F
HaCaT
G
H
I
J
K
siCtrl
siSMAD2
siSMAD3
siSMAD2/3
SMAD2
SMAD3
GAPDH
kDa
siCtrl
siSMAD2
siSMAD3
siSMAD2/3


Suppl. Fig S4

**Supplementary Figure S4. Related to Fig. 2.** TGF-β receptor signaling induces *VIM-AS1* v.2 expression in cancer cells and non-tumorigenic cells. (**A-F**) RT-qPCR of *VIM-AS1* v.2 and *SERPINE1* in H1299 (A), MDA-MB-231 (B), U3017MG (C), A431 (D), UT-SCC-12A (E) and HaCaT (F) cells stimulated with vehicle (Ctrl), 5 ng/mL TGF-β1, 5 μM LY2157299 TGFβRI inhibitor (TβRi) or combination of TGF-β with TβRi for 24 h. (**G-K**) Protein expression levels of SMAD2, SMAD3 and GAPDH (as loading control) in H1299 (G), MDA-MB-231 (H), U3017MG (I), UT-SCC-12A (J) and HaCaT (K) protein extracts of cells transiently transfected with the indicated siRNAs; densitometric values were normalized to the control siRNA (siCtrl). Representative immunoblots of at least two independent biological replicates along with molecular mass markers in kDa are shown. RT-qPCR analysis of *VIM-AS1* v.2 levels in each respective cell transiently transfected with the indicated siRNAs and stimulated with 5 ng/mL TGF-β1 for 24 h or not (Ctrl) is shown. The RT-qPCR values represent fold-change of RNA expression normalized to *GAPDH*, expressed relative to the unstimulated control level (Ctrl), and presented as mean values of at least two biological replicates ± SEM, in technical triplicates. P-values are shown based on two-way ANOVA, followed by multiple paired comparisons conducted by means of Bonferroni's post-test method. P-values: *$p \leq 0.05$; **$p \leq 0.01$; ***$p \leq 0.001$.

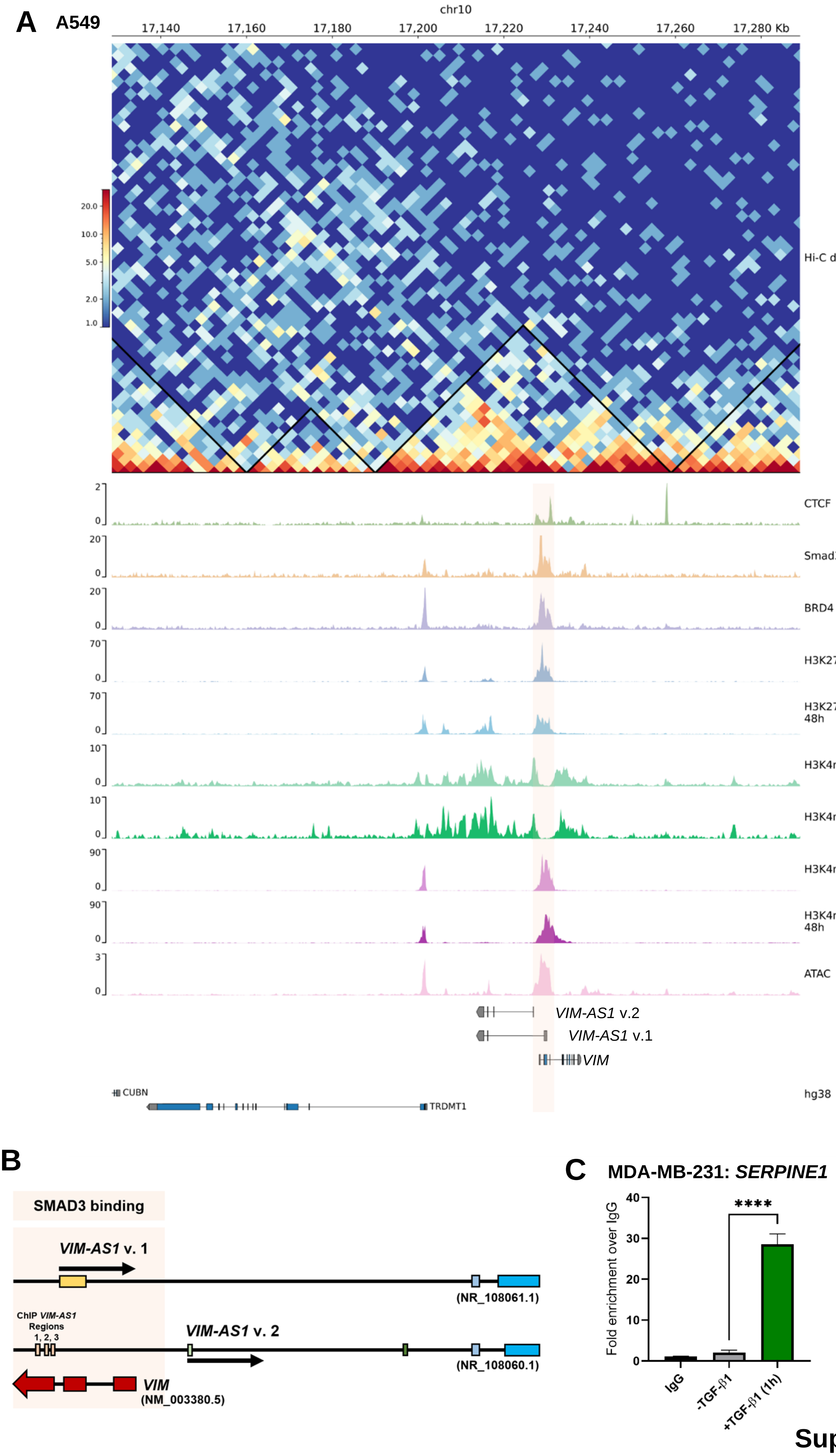
A
A549
chr10
17,140
17,160
17,180
17,200
17,220
17,240
17,260
17,280 Kb
20.0
10.0
5.0
2.0
1.0
Hi-C data
CTCF
Smad3
BRD4
H3K27ac Control
H3K27ac TGFB 48h
H3K4me Control
H3K4me TGFB 48h
H3K4me3 Control
H3K4me3 TGFB 48h
ATAC
VIM-AS1 v.2
VIM-AS1 v.1
VIM
CUBN
TRDMT1
hg38
B
SMAD3 binding
VIM-AS1 v. 1
(NR_108061.1)
ChIP VIM-AS1 Regions 1, 2, 3
VIM-AS1 v. 2
(NR_108060.1)
VIM
(NM_003380.5)
C
MDA-MB-231: SERPINE1
Fold enrichment over IgG
****
IgG
-TGF-β1
+TGF-β1 (1h)

Suppl. Fig S5

**Supplementary Figure S5. Related to Fig. 2.** (**A**) Genomic landscape of the *VIM-AS1* and *VIM* locus in A549 cells. Hi-C 1D interaction frequency heatmap in A549 cells (top, 3 kbp resolution). Black triangles in the heatmap indicate topologically associating domains (TADs), which were predicted with a 3 kbp step. CTCF, SMAD3 (TGF-β-stimulated for 1.5 h), BRD4 ChIP-seq profiles in the genomic region surrounding the *VIM-AS1* and *VIM* locus in A549 cells (GSE92782, GSE51510, GSE226487). ChIP-seq profiles of H3K27ac, H3K4me and H3K3me3 with or without TGF-β stimulation and ATAC-seq profile (CRA001325) are presented. (**B**) Schematic representation of the organization of the *VIM-AS1* gene. Exons are shown as boxes and introns as lines. Black arrows indicate the direction of the antisense transcription and the red arrow corresponds to the *VIM* mRNA transcript. The pair of primers for ChIP qPCR: Region 1 (R1), 2 (R2) and 3 (R3) used in the study are indicated. (**C**) ChIP-qPCR analysis for SMAD2/3 occupancy to the *SERPINE1* promoter in MDA-MB-231 cells stimulated with TGFβ1 for 1 h or not (-). Control IgG immunoprecipitation data is also shown. The data shown in C are presented as mean values of at least three biological replicates ± SEM, in technical triplicates. P-value is shown based on one-way ANOVA, followed by multiple paired comparisons conducted using Bonferroni's post-test method (****$p \leq 0.0001$).

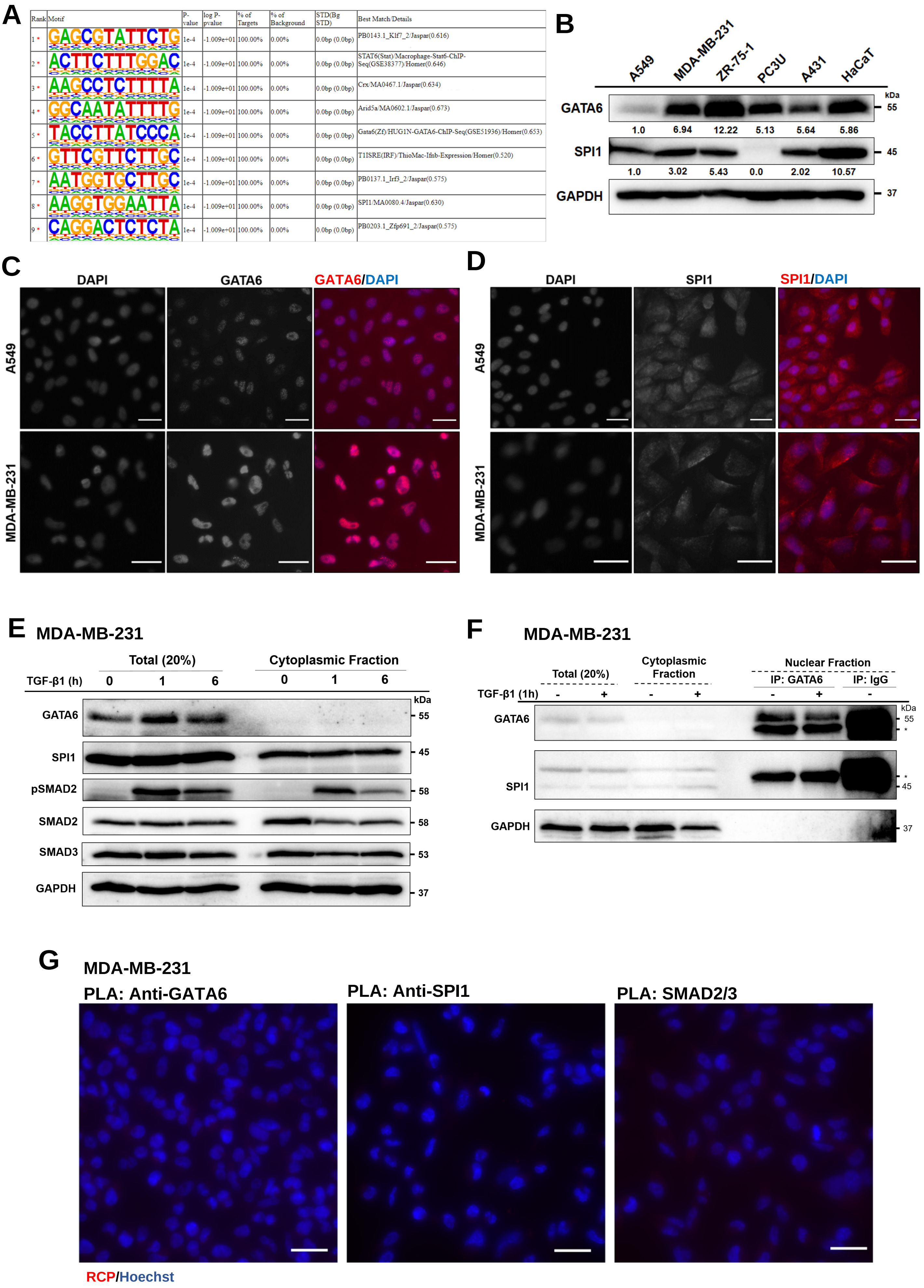
A
Rank Motif P-value log P-pvalue % of Targets % of Background STD(Bg STD) Best Match/Details
1 GAGCGTATTCTG 1e-4 -1.009e+01 100.00% 0.00% 0.0bp (0.0bp) PB0143.1_Klf7_2/Jaspar(0.616)
2 ACTTCTTTGGAC 1e-4 -1.009e+01 100.00% 0.00% 0.0bp (0.0bp) STAT6(Stat)/Macrophage-Stat6-ChIP-Seq(GSE38377)/Homer(0.646)
3 AAGCCTCTTTTA 1e-4 -1.009e+01 100.00% 0.00% 0.0bp (0.0bp) Crx/MA0467.1/Jaspar(0.634)
4 GGCAATATTTTG 1e-4 -1.009e+01 100.00% 0.00% 0.0bp (0.0bp) Arid5a/MA0602.1/Jaspar(0.673)
5 TACCTTATCCCA 1e-4 -1.009e+01 100.00% 0.00% 0.0bp (0.0bp) Gata6(Zf)/HUG1N-GATA6-ChIP-Seq(GSE51936)/Homer(0.653)
6 GTTCGTTCTTGC 1e-4 -1.009e+01 100.00% 0.00% 0.0bp (0.0bp) T1ISRE(IRF)/ThioMac-Ifnb-Expression/Homer(0.520)
7 AATGGTGCTTGC 1e-4 -1.009e+01 100.00% 0.00% 0.0bp (0.0bp) PB0137.1_Irf3_2/Jaspar(0.575)
8 AAGGTGGAATTA 1e-4 -1.009e+01 100.00% 0.00% 0.0bp (0.0bp) SPI1/MA0080.4/Jaspar(0.630)
9 CAGGACTCTCTA 1e-4 -1.009e+01 100.00% 0.00% 0.0bp (0.0bp) PB0203.1_Zfp691_2/Jaspar(0.575)
B
A549 MDA-MB-231 ZR-75-1 PC3U A431 HaCaT
kDa
GATA6 55
1.0 6.94 12.22 5.13 5.64 5.86
SPI1 45
1.0 3.02 5.43 0.0 2.02 10.57
GAPDH 37
C
DAPI GATA6 GATA6/DAPI
A549
MDA-MB-231
D
DAPI SPI1 SPI1/DAPI
A549
MDA-MB-231
E
MDA-MB-231
Total (20%) Cytoplasmic Fraction
TGF-β1 (h) 0 1 6 0 1 6
kDa
GATA6 55
SPI1 45
pSMAD2 58
SMAD2 58
SMAD3 53
GAPDH 37
F
MDA-MB-231
Total (20%) Cytoplasmic Fraction Nuclear Fraction
IP: GATA6 IP: IgG
TGF-β1 (1h) - + - + - + -
kDa
GATA6 55 *
SPI1 * 45
GAPDH 37
G
MDA-MB-231
PLA: Anti-GATA6 PLA: Anti-SPI1 PLA: SMAD2/3
RCP/Hoechst

Suppl. Fig S6

**Supplementary Figure S6. Related to Fig 2.** GATA6 and SPI1 interact with SMADs. (**A**) Homer analysis of significant DNA binding motifs on the *VIM-AS1* v.2 promoter sequence. (**B**) Protein expression levels of GATA6, SPI1 and GAPDH (as loading control) in the indicated cell lines, and densitometric values normalized to A549. Representative immunoblots of at least two independent biological replicates along with molecular mass markers in kDa are shown. (**C, D**) Representative immunofluorescence microscopy pictures of A549 and MDA-MB-231 cells for GATA6 (C) and SPI1 (D). The proteins (red) and nuclei (DAPI; blue) are labeled. Scale bars, 50 μm. (**E**) Input (20%) of total protein expression levels and cytoplasmic fractions for the transcription factors (TFs) and GAPDH in MDA-MB-231 cells incubated with vehicle (-) or 5 ng/mL TGF-β1 for 1 and 6 h, related to Figure 2E. Representative immunoblots of three independent biological replicates along with molecular mass markers in kDa are shown. (**F**) Protein complex formation between GATA6 and SPI1. MDA-MB-231 cells were incubated with vehicle (-) or 5 ng/mL TGF-β1 for 1 h and the nuclear protein fraction lysates were immunoprecipitated (IP) with Dynabeads™ Protein A associated to GATA6 antibody or non-specific IgG, followed by SDS-PAGE and immunoblotting for GATA6, SPI1 and GAPDH. Input (20%) of total protein expression levels and cytoplasmic fractions are also shown. A star indicates the IgG band recognized by the secondary antibody confirming immunoprecipitation in every sample. Representative immunoblots of two independent biological replicates along with molecular mass markers in kDa are shown. (**G**) Negative control with single antibody incubation for the PLA used to validate the co-localization of SMAD2/3 with GATA6 or SPI1 in MDA-MB-231 cells. Nuclei are shown in blue (Hoechst), and PLA rolling circle amplification product in red.

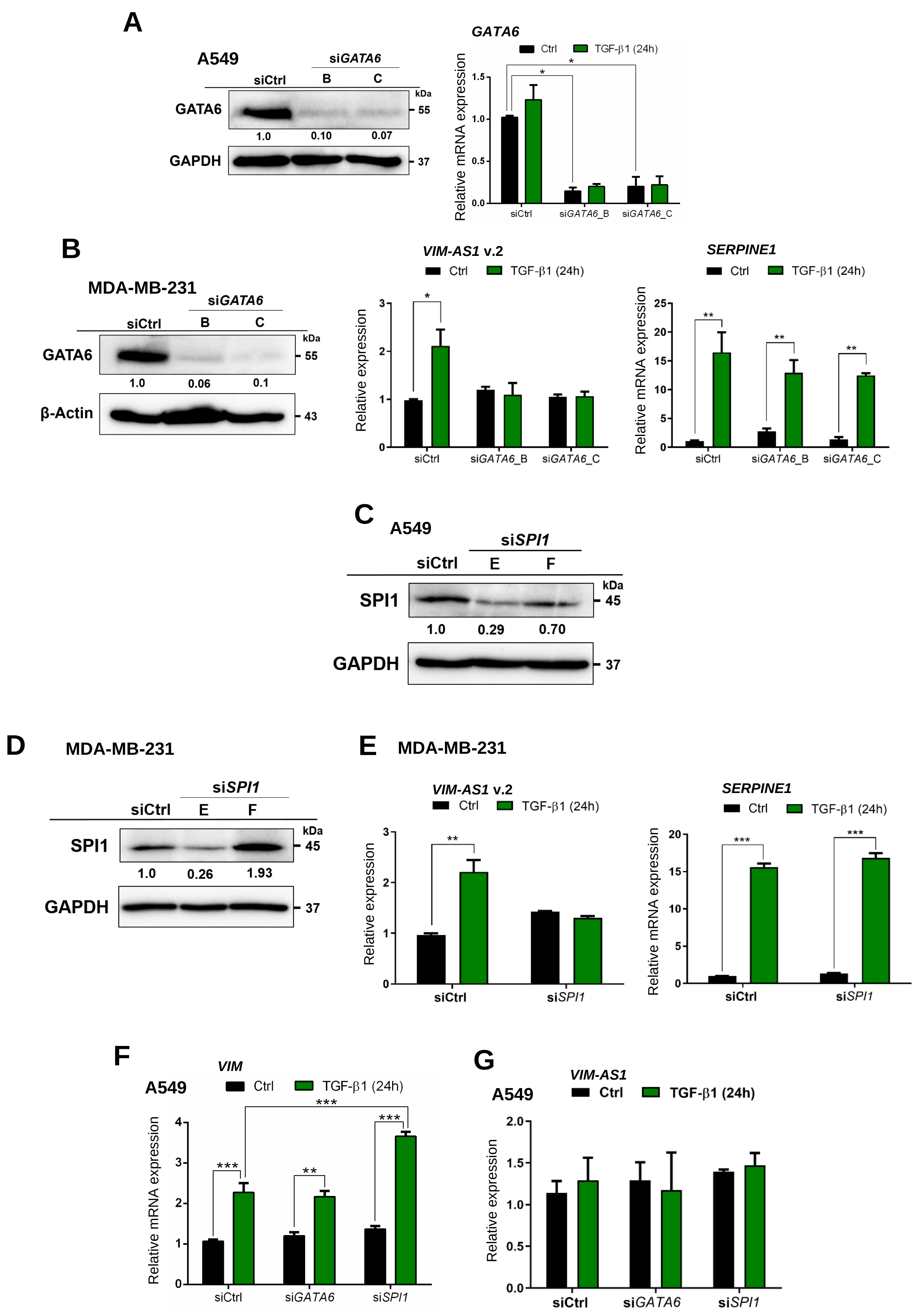
A
A549
siGATA6
siCtrl
B
C
GATA6
kDa
55
1.0
0.10
0.07
GAPDH
37
GATA6
Ctrl
TGF-β1 (24h)
Relative mRNA expression
siCtrl
siGATA6_B
siGATA6_C
B
MDA-MB-231
siGATA6
siCtrl
B
C
GATA6
kDa
55
1.0
0.06
0.1
β-Actin
43
VIM-AS1 v.2
Ctrl
TGF-β1 (24h)
Relative expression
siCtrl
siGATA6_B
siGATA6_C
SERPINE1
Ctrl
TGF-β1 (24h)
Relative mRNA expression
siCtrl
siGATA6_B
siGATA6_C
C
A549
siSPI1
siCtrl
E
F
SPI1
kDa
45
1.0
0.29
0.70
GAPDH
37
D
MDA-MB-231
siSPI1
siCtrl
E
F
SPI1
kDa
45
1.0
0.26
1.93
GAPDH
37
E
MDA-MB-231
VIM-AS1 v.2
Ctrl
TGF-β1 (24h)
Relative expression
siCtrl
siSPI1
SERPINE1
Ctrl
TGF-β1 (24h)
Relative mRNA expression
siCtrl
siSPI1
F
A549
VIM
Ctrl
TGF-β1 (24h)
Relative mRNA expression
siCtrl
siGATA6
siSPI1
G
A549
VIM-AS1
Ctrl
TGF-β1 (24h)
Relative expression
siCtrl
siGATA6
siSPI1

**Suppl. Fig S7**

**Supplementary Figure S7. Related to Fig. 2.** TGF-β induces *VIM-AS1* v.2 expression via a SMAD-GATA6-SPI1 complex. (**A**) Protein expression levels of GATA6 and GAPDH (as loading control) in A549 protein extracts transiently transfected with the indicated siRNAs against GATA6, and densitometric values normalized to the control siRNA (siCtrl). RT-qPCR analysis of *GATA6* mRNA levels in A549 cells transiently transfected with the indicated siRNAs and stimulated with 5 ng/mL TGF-β1 for 24 h or not (Ctrl). (**B**) Protein expression levels of GATA6 and β-Actin (as loading control) in MDA-MB-231 protein extracts transiently transfected with the indicated siRNAs against GATA6, and densitometric values normalized to the control siRNA (siCtrl). RT-qPCR analysis of *VIM-AS1* v.2 and *SERPINE1* RNA levels in MDA-MB-231 cells transiently transfected with the indicated siRNAs and stimulated with 5 ng/mL TGF-β1 for 24 h or not (Ctrl). (**C, D**) Expression levels of SPI1 protein in A549 (C) and MDA-MB-231 (D) cells transiently transfected with control (siCtrl) or specific siRNA targeting *SPI1* (note si*SPI1*_E as the most efficient siRNA), and densitometric values were normalized to the control siRNA. Representative immunoblots in panels A-D of at least two independent biological replicates along with molecular mass markers in kDa. (**E**) RT-qPCR analysis of *VIM-AS1* v.2 and *SERPINE1* RNA levels in MDA-MB-231 cells transiently transfected with siCtrl or si*SPI1*_E and stimulated with 5 ng/mL TGF-β1 for 24 h or not (Ctrl). (**F, G**) RT-qPCR analysis of *VIM* (F) and *VIM-AS1* (v.1 and v.2) (G) RNA levels in A549 cells transiently transfected with siCtrl, siGATA6_B or si*SPI1*_E and stimulated with 5 ng/mL TGF-β1 for 24 h or not (Ctrl). The RT-qPCR values in A, B, E-G represent the fold-change of RNA expression normalized to *GAPDH*, expressed relative to the unstimulated control level (Ctrl), and presented as mean values of at least two biological replicates ± SEM, in technical triplicates. P-values are shown based on two-way ANOVA, followed by multiple paired comparisons conducted by means of Bonferroni's post-test method. P-values: *$p \leq 0.05$; **$p \leq 0.01$; ***$p \leq 0.001$.

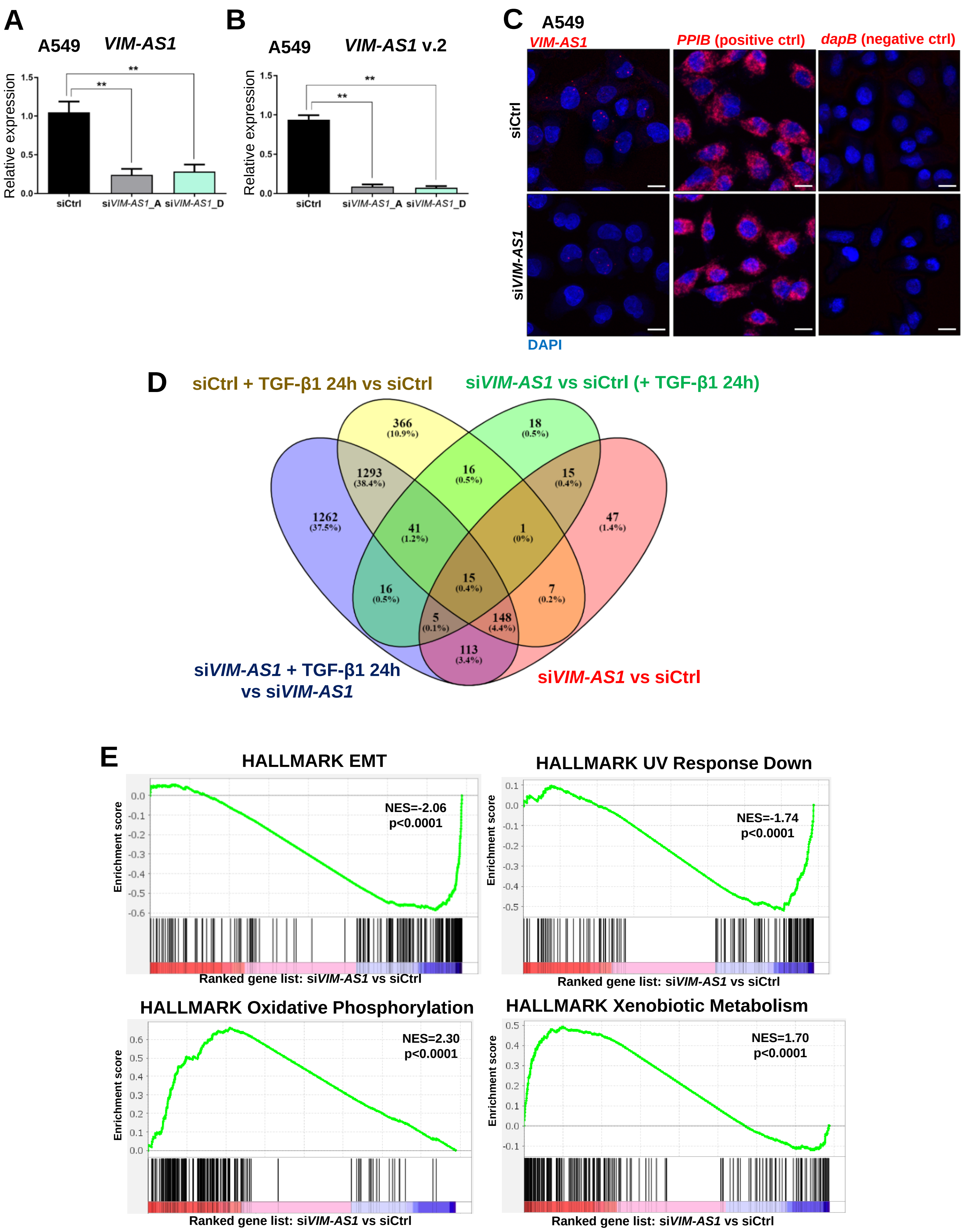
A
A549 VIM-AS1
Relative expression
**
siCtrl
siVIM-AS1_A
siVIM-AS1_D
B
A549 VIM-AS1 v.2
C
A549
VIM-AS1
PPIB (positive ctrl)
dapB (negative ctrl)
siCtrl
siVIM-AS1
DAPI
D
siCtrl + TGF-β1 24h vs siCtrl
siVIM-AS1 vs siCtrl (+ TGF-β1 24h)
siVIM-AS1 + TGF-β1 24h vs siVIM-AS1
siVIM-AS1 vs siCtrl
366 (10.9%)
18 (0.5%)
1293 (38.4%)
16 (0.5%)
15 (0.4%)
1262 (37.5%)
41 (1.2%)
1 (0%)
47 (1.4%)
16 (0.5%)
15 (0.4%)
7 (0.2%)
5 (0.1%)
148 (4.4%)
113 (3.4%)
E
HALLMARK EMT
NES=-2.06
p<0.0001
HALLMARK UV Response Down
NES=-1.74
p<0.0001
HALLMARK Oxidative Phosphorylation
NES=2.30
p<0.0001
HALLMARK Xenobiotic Metabolism
NES=1.70
p<0.0001
Enrichment score
Ranked gene list: siVIM-AS1 vs siCtrl

**Suppl. Fig S8**

**Supplementary Figure S8. Related to Fig. 3.** RNA-seq of A549 cells upon *VIM-AS1* silencing. (**A, B**) RT-qPCR analysis of *VIM-AS1* v.1 and v.2 (A) and *VIM-AS1* v.2 (B) levels in A549 cells transiently transfected with the control siRNA (siCtrl) or si*VIM-AS1*_A or si*VIM-AS1*_D. The RT-qPCR values represent the fold-change of RNA expression normalized to *GAPDH*, expressed relative to the siCtrl level, and presented as mean values of three biological replicates ± SEM, in technical triplicates. P-values are shown based on one-way ANOVA, followed by multiple paired comparisons conducted by means of Bonferroni's post-test method. P-value: **p ≤ 0.01. (**C**) RNAscope for *VIM-AS1* and respective controls in A549 cells transiently transfected with the control siRNA (siCtrl) or si*VIM-AS1*_D. Images were acquired with a Leica Stellaris 5 confocal microscope. Scale bar 25 µm. (**D**) Venn diagram illustrating the overlap between differentially expressed genes in A549 cells transiently transfected with control siRNA (siCtrl) or siVIM-AS1 and stimulated or not with 5 ng/mL TGF-β1 for 24 h. (**E**) GSEA of significant correlations between silencing of *VIM-AS1* expression with the gene signature of EMT (down), UV response downregulated (down), oxidative phosphorylation (up) and xenobiotic metabolism (up).

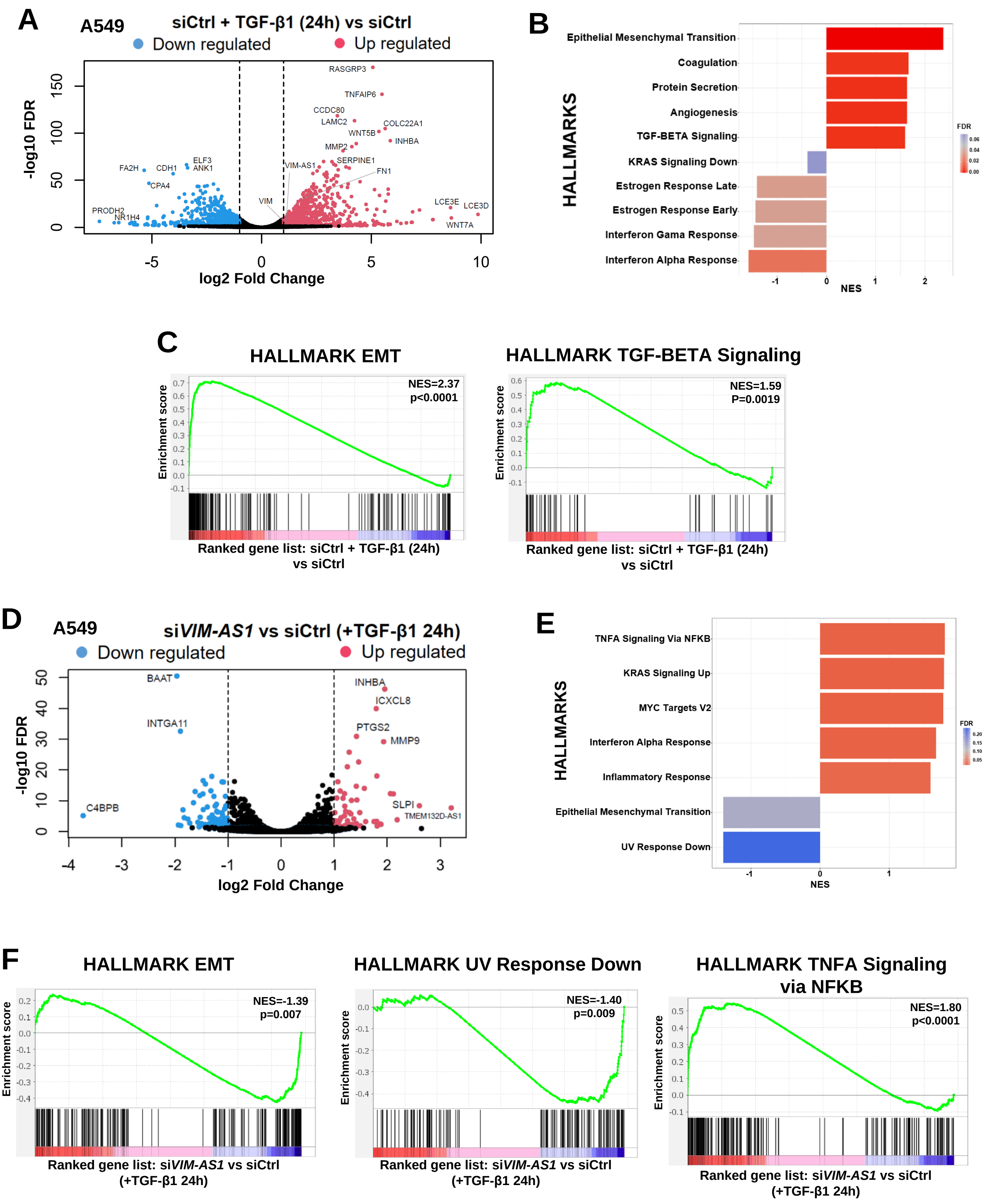
A
A549
siCtrl + TGF-β1 (24h) vs siCtrl
Down regulated
Up regulated
-log10 FDR
log2 Fold Change
RASGRP3
TNFAIP6
CCDC80
LAMC2
COLC22A1
WNT5B
INHBA
MMP2
SERPINE1
FN1
VIM-AS1
VIM
ELF3
ANK1
FA2H
CDH1
CPA4
PRODH2
NR1H4
LCE3E
LCE3D
WNT7A
B
HALLMARKS
Epithelial Mesenchymal Transition
Coagulation
Protein Secretion
Angiogenesis
TGF-BETA Signaling
KRAS Signaling Down
Estrogen Response Late
Estrogen Response Early
Interferon Gama Response
Interferon Alpha Response
NES
FDR
C
HALLMARK EMT
NES=2.37
p<0.0001
Enrichment score
Ranked gene list: siCtrl + TGF-β1 (24h) vs siCtrl
HALLMARK TGF-BETA Signaling
NES=1.59
P=0.0019
Enrichment score
Ranked gene list: siCtrl + TGF-β1 (24h) vs siCtrl
D
A549
siVIM-AS1 vs siCtrl (+TGF-β1 24h)
Down regulated
Up regulated
-log10 FDR
log2 Fold Change
BAAT
INTGA11
C4BPB
INHBA
CXCL8
PTGS2
MMP9
SLPI
TMEM132D-AS1
E
HALLMARKS
TNFA Signaling Via NFKB
KRAS Signaling Up
MYC Targets V2
Interferon Alpha Response
Inflammatory Response
Epithelial Mesenchymal Transition
UV Response Down
NES
FDR
F
HALLMARK EMT
NES=-1.39
p=0.007
Enrichment score
Ranked gene list: siVIM-AS1 vs siCtrl (+TGF-β1 24h)
HALLMARK UV Response Down
NES=-1.40
p=0.009
Enrichment score
Ranked gene list: siVIM-AS1 vs siCtrl (+TGF-β1 24h)
HALLMARK TNFA Signaling via NFKB
NES=1.80
p<0.0001
Enrichment score
Ranked gene list: siVIM-AS1 vs siCtrl (+TGF-β1 24h)

**Suppl. Fig S9**

**Supplementary Figure S9. Related to Fig. 3.** RNA-seq of A549 cells upon TGF-β stimulation and *VIM-AS1* silencing. (**A, D**) Volcano plots depicting the up- (red) and down- (blue) regulated genes in A549 cells transiently transfected with control siRNA (siCtrl) (A) or transfected with si*VIM-AS1* (D), with or without TGF-β stimulation for 24 h. The vertical dotted lines indicate the expression fold-change threshold ($-1 \leq \log 2 \geq 1$). (**B, C, E-F**) The GSEA-Hallmark database indicates strong upregulation of EMT and TGF-β signaling after TGF-β signaling in siCtrl cells (B), with detailed diagrams (C) and downregulation of EMT, UV response and upregulation of TNF-α signaling via NF-κB after *VIM-AS1* silencing (E), with details shown in F.

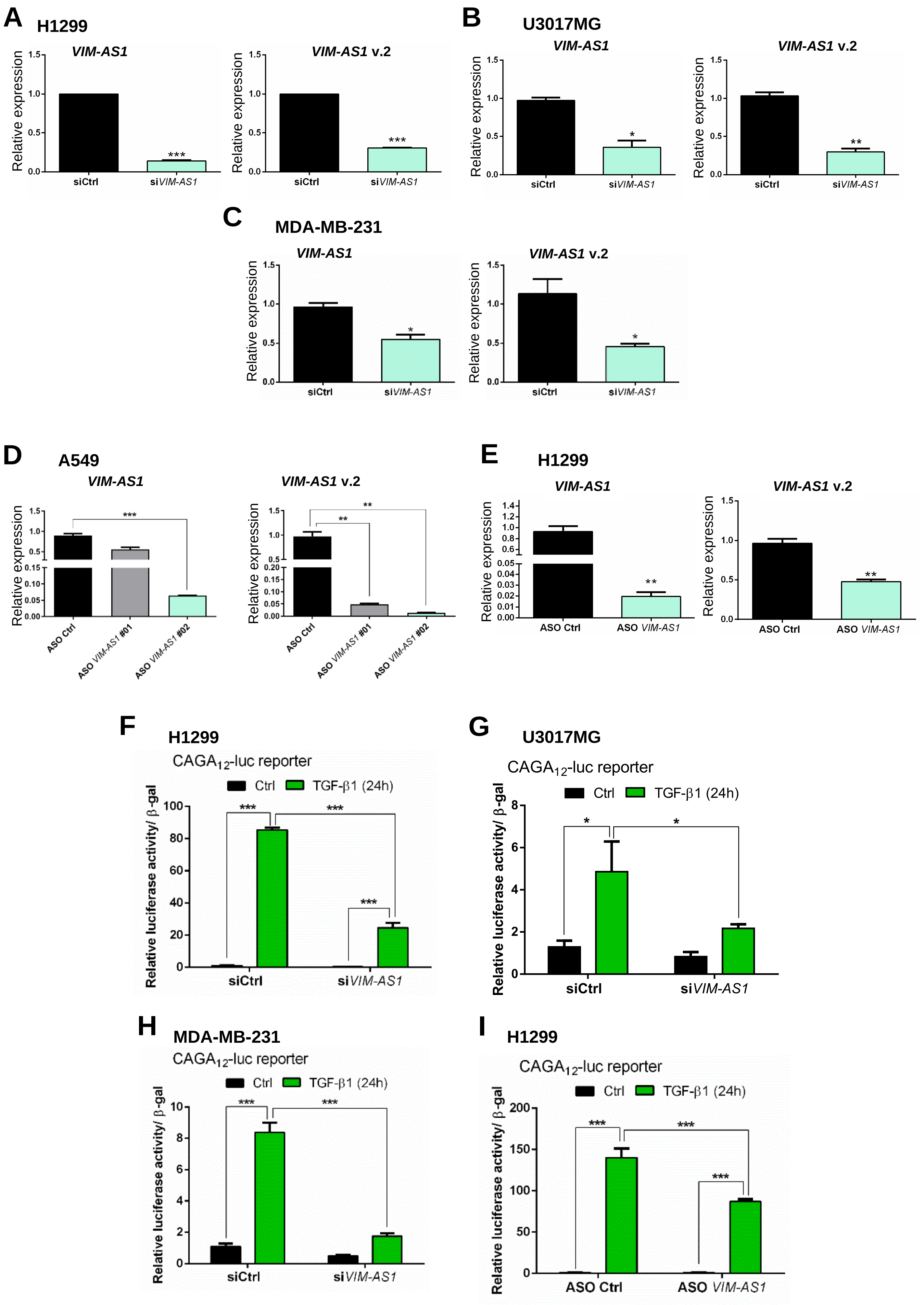


**Suppl. Fig S10**

**Supplementary Figure S10. Related to Fig. 3.** *VIM-AS1* silencing decreases TGF-β signaling. (**A-C**) RT-qPCR analysis of *VIM-AS1* (v.1 and v.2) and *VIM-AS1* (v.2) levels in H1299 (A), U3017MG (B) and MDA-MB-231 (C) cells transiently transfected with control siRNA (siCtrl) or si*VIM-AS1*. (**D, E**) RT-qPCR analysis of *VIM-AS1* levels in A549 cells (D) transiently transfected with the indicated ASO control (Ctrl) or ASOs *VIM-AS1* (#01 and #02, note the latest as the most efficient) or in H1299 cells (E) transfected with ASO Ctrl or ASO *VIM-AS1* (#02). Values represent fold-change of *VIM-AS1* expression normalized to *GAPDH* and expressed relative to the Ctrl level. The RT-qPCR values represent the fold-change of RNA expression normalized to *GAPDH*, expressed relative to the siCtrl level, and presented as mean values of at three biological replicates ± SEM, in technical triplicates. (**F-I**) Relative luciferase activity generated in H1299 (F), U3017MG (G) and MDA-MB-231 (H) cells transiently transfected with siCtrl or si*VIM-AS1*, or H1299 cells (I) transfected with ASO Ctrl or ASO *VIM-AS1* by additional transfection of the TGF-β-inducible $CAGA_{12}$-luc reporter, normalized to β-galactosidase activity generated by a co-transfected reporter, after stimulation of the cells with vehicle (Ctrl) or 5 ng/mL TGF-β1 for 24 h. Data are presented as mean values of three biological replicates ± SEM, each in technical triplicates. P-values in panels A-C are shown based on unpaired student's *t*-test with Welch's correction. P-values in panels D and E are shown based on one-way ANOVA, followed by multiple paired comparisons conducted by means of Bonferroni's post-test method. P-values in panels F-I are shown based on two-way ANOVA, followed by multiple paired comparisons conducted by means of Bonferroni's post-test method. P-values: *p ≤ 0.05; **p ≤ 0.01; ***p ≤ 0.001.

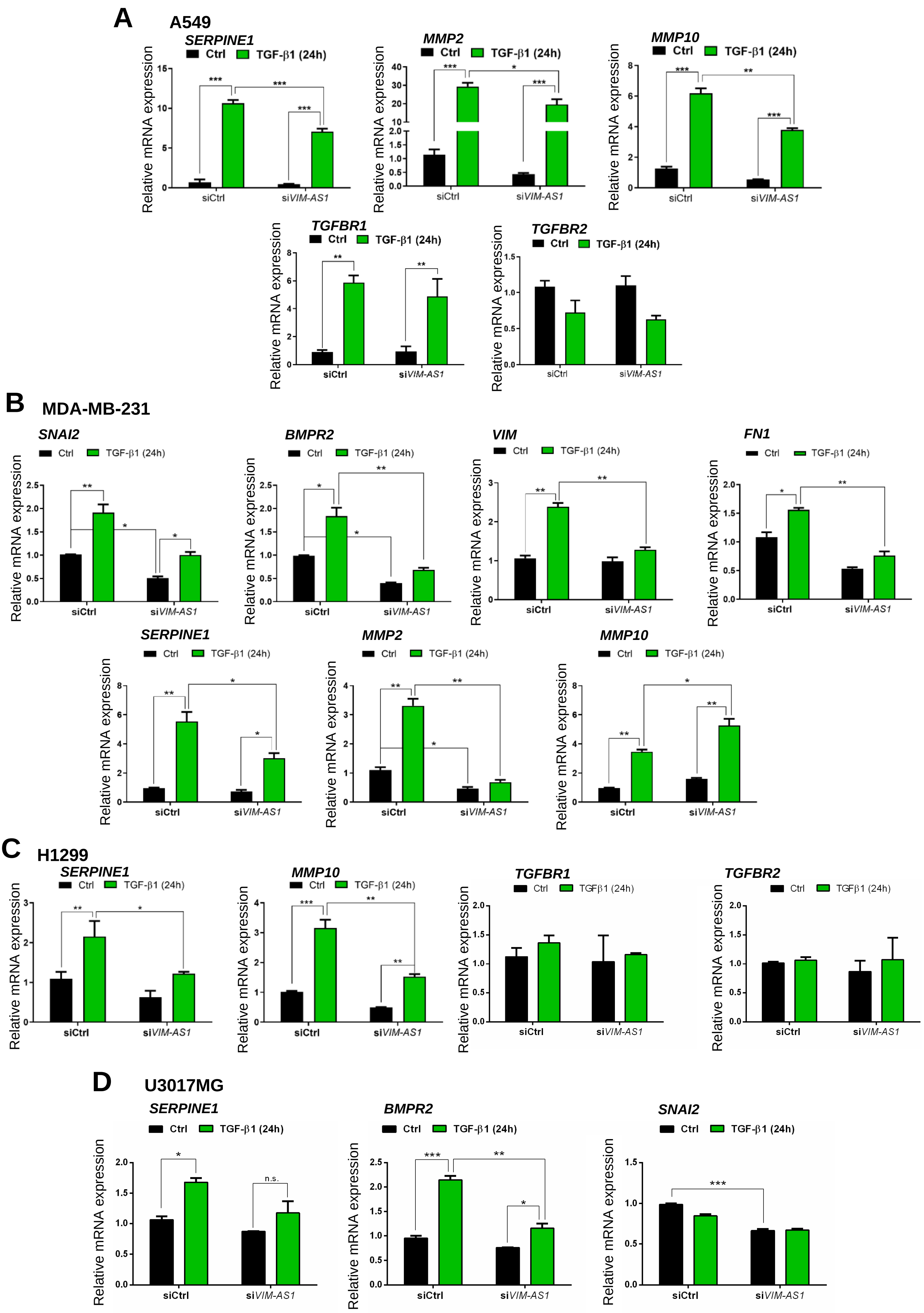


Suppl. Fig S11

**Supplementary Figure S11. Related to Fig. 3.** *VIM-AS1* silencing decreases the mRNA expression of TGF-β-responsive genes. (**A-D**) RT-qPCR analysis of the indicated mRNA levels in A549 (A), MDA-MB-231 (B), H1299 (C) and U3017MG (D) cells transiently transfected with siCtrl or si*VIM-AS1* and incubated or not with 5 ng/mL TGF-β1 for 24 h. Data are presented as mean values of three biological replicates ± SEM, each in technical triplicates. P-values in panels A-D are shown based on two-way ANOVA, followed by multiple paired comparisons conducted by means of Bonferroni's post-test method. P-values: *p ≤ 0.05; **p ≤ 0.01; ***p ≤ 0.001.

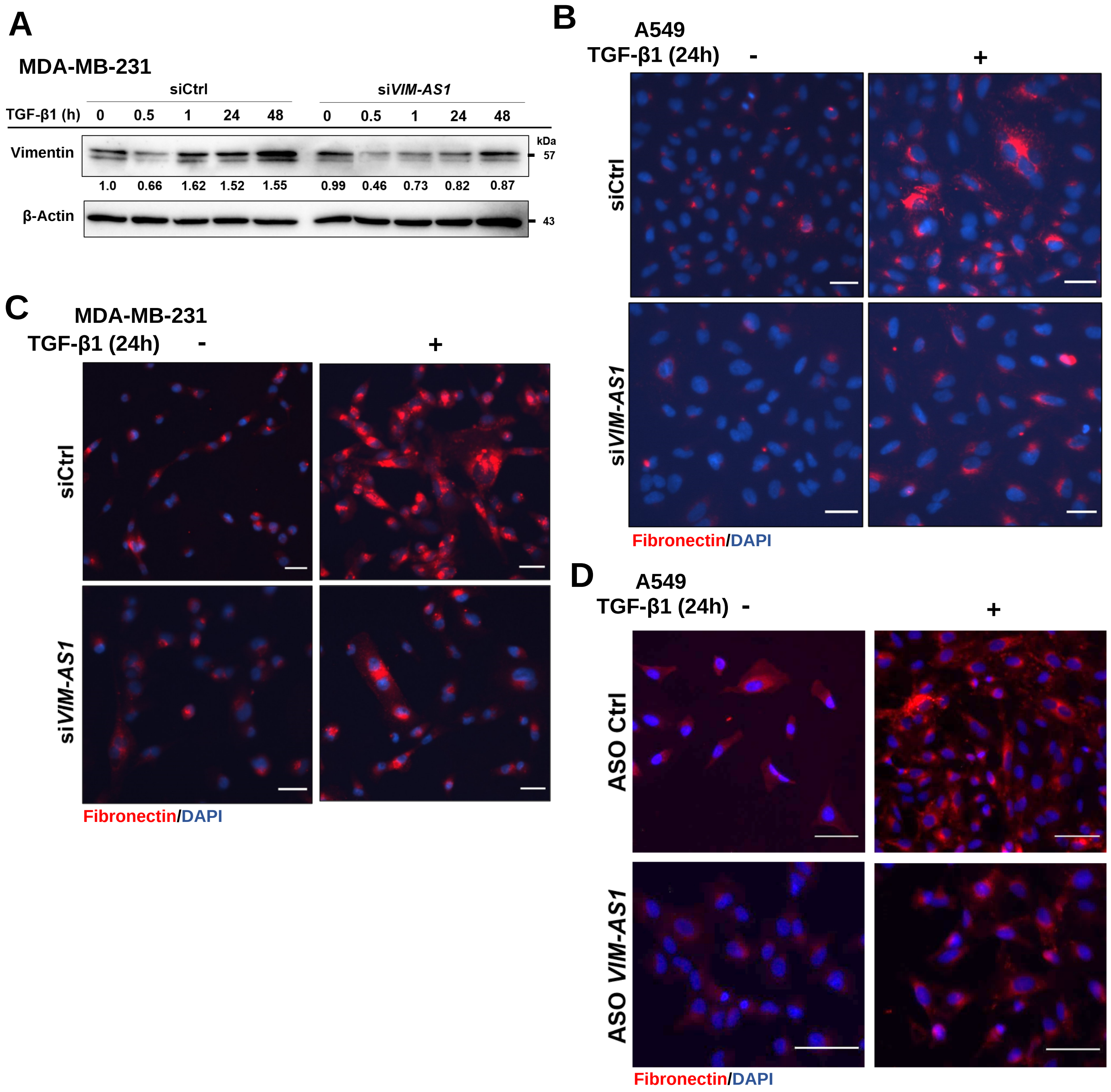


Suppl. Fig S12

**Supplementary Figure S12. Related to Fig. 3.** *VIM-AS1* silencing decreases the protein expression of TGF-β-responsive genes. (**A**) Protein expression levels of Vimentin and β-Actin (as loading control) in cellular extracts of MDA-MB-231 cells that were transiently transfected with siCtrl or si*VIM-AS1* and stimulated or not with 5 ng/mL TGF-β1 for the indicated periods. Densitometric values normalized to the unstimulated control. Representative immunoblots of three independent biological replicates along with molecular mass markers in kDa are shown. (**B-D**) Representative immunofluorescence microscopy pictures of A549 (B) and MDA-MB-231 (C) cells transiently transfected with siCtrl or si*VIM-AS1,* or A549 cells transfected with ASO Ctrl or ASO *VIM-AS1* (D), and stimulated with vehicle (Ctrl) or 5 ng/mL TGF-β1 for 24 h. The Fibronectin (red) protein and nuclei (DAPI; blue) are labeled. Scale bars, 50 μm.

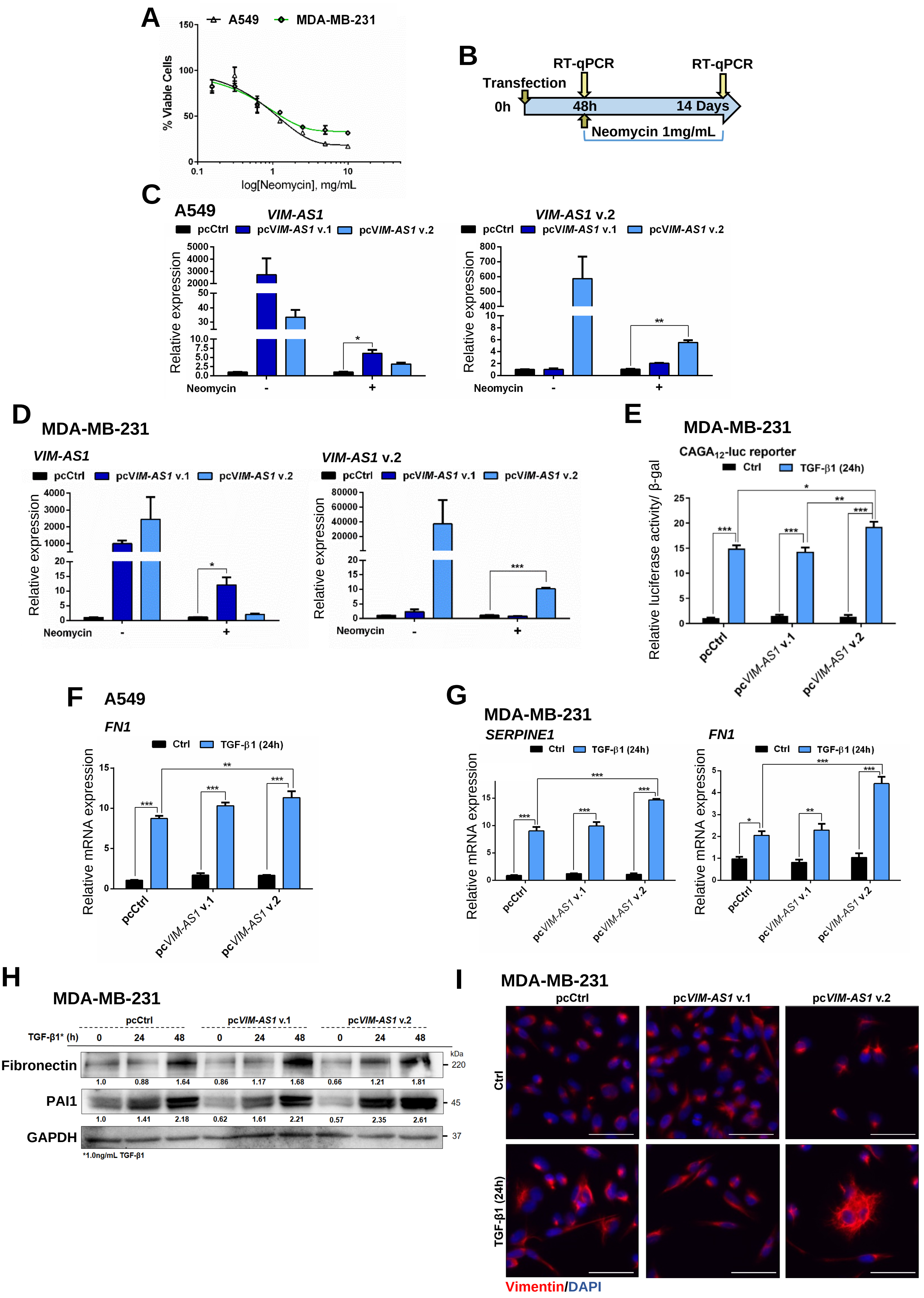


Suppl. Fig S13

**Supplementary Figure S13. Related to Fig. 3.** *VIM-AS1* v.2 overexpression enhances TGF-β signaling. (**A**) Viability curves of A549 and MDA-MB-231 cells in the presence of increasing concentrations of neomycin (logarithmic scale). Note the lack of significant differences. (**B**) Schematic outline of the neomycin selection and assessment in A549 and MDA-MB-231 cells. (**C, D**) RT-qPCR analysis of *VIM-AS1* levels in A549 (C) and MDA-MB-231 (D) cells transfected with empty vector (Ctrl) or pcDNA(pc)-*VIM-AS1* v.1 or pc*VIM-AS1* v.2, before and after selection with neomycin. Values represent fold-change of *VIM-AS1* expression normalized to *GAPDH* and expressed relative to the respective pcCtrl level. (**E**) Relative $CAGA_{12}$-luciferase activity in MDA-MB-231 cells overexpressing *VIM-AS1* and selected by neomycin, followed by stimulation with 5 ng/mL TGF-β1 for 24 h. (**F, G**) RT-qPCR analysis of the indicated mRNA levels in A549 (F; *FN1*) and MDA-MB-231 (G; *SERPINE1* and *FN1*) cells selected by neomycin as in B-D, followed by stimulation or not with 5 ng/mL TGF-β1 for 24 h. The RT-qPCR values represent the fold-change of RNA expression normalized to *GAPDH*, expressed relative to the pcCtrl level, and presented as mean values of at three biological replicates ± SEM, in technical triplicates. P-values in panels C-G are shown based on two-way ANOVA, followed by multiple paired comparisons conducted by means of Bonferroni's post-test method. P-values: *p ≤ 0.05; **p ≤ 0.01; ***p ≤ 0.001. (**H**) Protein expression levels of Fibronectin and PAI1 in cellular extracts of MDA-MB-231 cells selected by neomycin as in B and D and stimulated or not with 1 ng/mL TGF-β1 for the indicated periods. Densitometric values normalized to the unstimulated pcCtrl. Representative immunoblots of three independent biological replicates along with molecular mass markers in kDa are shown. (**I**) Representative immunofluorescence microscopy pictures of MDA-MB-231 cells transfected with empty vector (pcCtrl), pcDNA(pc)-*VIM-AS1* v.1 or pc*VIM-AS1* v.2 after selection with neomycin (B and D), and stimulated with vehicle (Ctrl) or 5 ng/mL TGF-β1 for 24 h. The Vimentin protein (red) and nuclei (DAPI; blue) are labeled. Scale bars, 50 μm.

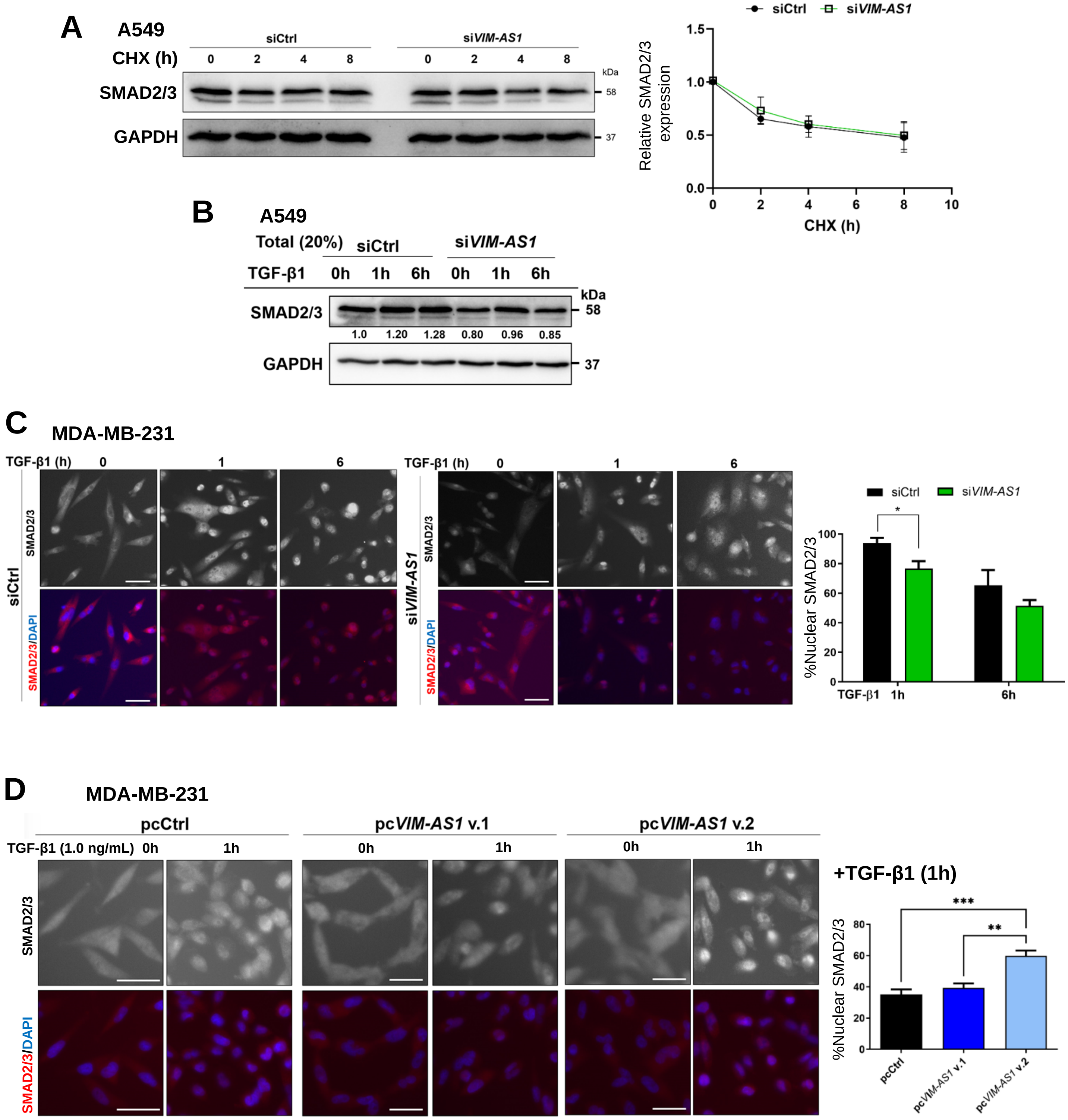


Suppl. Fig S14

**Supplementary Figure S14. Related to Fig. 4.** *VIM-AS1* v.2 enhances receptor-SMAD2/3 nuclear accumulation. (**A**) Immunoblot of SMAD2/3 and GAPDH (as loading control) in A549 extracts after treatment with vehicle (−) or 40 μg/mL cycloheximide (CHX) for the indicated time periods. Quantification of band intensities are presented as the mean ± SEM of three independent sets of experiments and normalized to the 0 h control. (**B**) Representative immunoblot of the input (20%) of total protein expression levels of SMAD2/3 and GAPDH (as loading control) in A549 cells transiently transfected with siRNA control (siCtrl) or siVIM-AS1 and stimulated or not with 5 ng/mL TGF-β1 for the indicated periods (as in Fig. 4C). (**C**) Representative immunofluorescence microscopy pictures of MDA-MB-231 cells transiently transfected with siCtrl or si*VIM-AS1* and stimulated with vehicle (0 h) or 5 ng/mL TGF-β1 for 1 or 6 h. (**D**) Representative immunofluorescence microscopy pictures of MDA-MB-231 cells transfected with empty vector (pcCtrl) or pc-*VIM-AS1* v.1 or pc*VIM-AS1* v.2 after neomycin selection and stimulated or not with 1 ng/mL TGF-β1 for 1 h. In panels C and D, the SMAD2/3 proteins (black and white or red) and nuclei (blue; DAPI) are labeled. Values in C and D represent the percentage of SMAD2/3-stained nuclear intensity, normalized to the 0 h time point. P-values in C are based on two-way ANOVA or in D on one-way ANOVA, followed by multiple paired comparisons conducted using Bonferroni's post-test method. P-values: *p ≤ 0.05; **p ≤ 0.01; ***p ≤ 0.001. Scale bars, 50 μm.

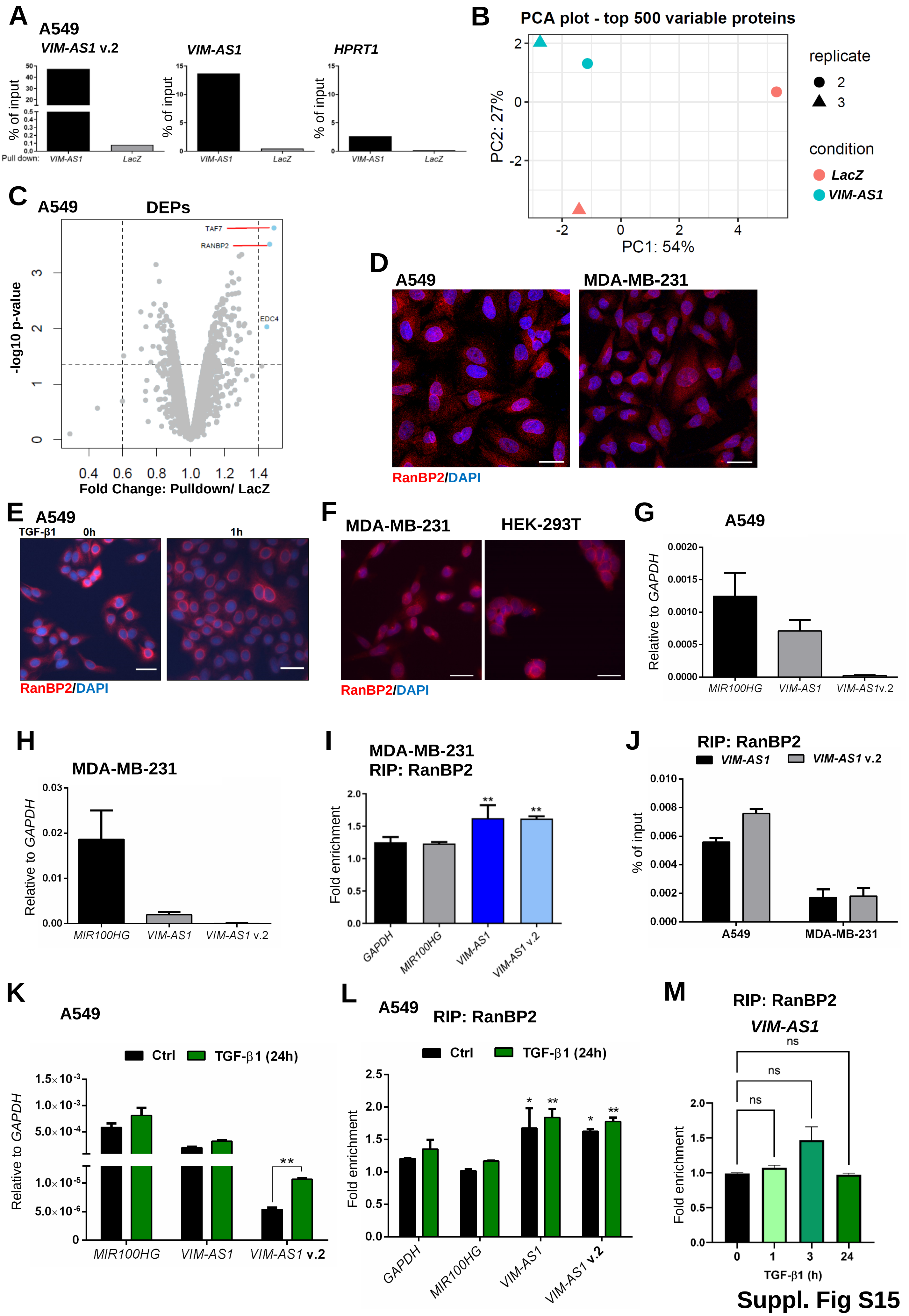


**Suppl. Fig S15**

**Supplementary Figure S15. Related to Fig. 5.** *VIM-AS1* binds to RanBP2 (Nucleoporin 358; Nup358). (**A**) Modified chromatin oligo-affinity precipitation (ChOP), using biotinylated oligonucleotides complementary for *VIM-AS1* or *LacZ* (as negative control) incubated with A549 RNA cell extract and analysed by RT-qPCR for the indicated three mRNAs. (**B**) PCA of statistically significant protein expression differences among *VIM-AS1* and *LacZ* interacting proteins in A549 cells identified using ChOP-MS in two independent biological replicates. (**C**) Volcano plot showing *VIM-AS1* interacting proteins in A549 cells identified using ChOP-MS in two independent biological replicates. DEPs were employed to conduct the differential enrichment analysis. Three top hit proteins are marked. Dotted lines indicate the respective thresholds. (**D**) Representative immunofluorescence confocal microscopy pictures of A549 and MDA-MB-231 cells. RanBP2 (red) and nuclei (DAPI; blue) are labeled. Scale bar 40 µm. Images in panels C-E were acquired with a Leica Stellaris 5 confocal microscope. (**E, F**) Representative immunofluorescence microscopy pictures of A549 cells stimulated with TGF-β1 or not for 1 h (E), and of MDA-MB-231 and HEK293T (F) cells. RanBP2 (red) and nuclei (DAPI; blue) are labeled. Scale bar 15 µm. (**G, H**) RT-qPCR of A549 (G) and MDA-MB-231 (H) cells used as input (10%) of RanBP2-specific RIP analysis (as in Fig. 5D and panel I). RNA immunoprecipitated (RIP) levels were normalized to *GAPDH*. (**I**) Fold-enrichment of the RanBP2-specific RIP relative to the IgG control in MDA-MB-231 cells is reported for *GAPDH*, *MIR100HG*, *VIM-AS1* and *VIM-AS1* v.2. (**J**) RanBP2-specific RIP analysis in A549 and MDA-MB-231 cells. Percent of the RanBP2-specific RIP relative to the input RNA is reported for *VIM-AS1* (v.1 and v.2) and *VIM-AS1* (v.2) lncRNAs. (**K**) RT-qPCR of A549 cells stimulated or not with TGF-β1 for 24 h used as input (10%) of RanBP2-specific RIP analysis (as in panel L). LncRNA expression levels were normalized to *GAPDH*. (**L**) Fold-enrichment of the RanBP2-specific RIP relative to the IgG control in A549 cells is reported for *GAPDH*, *MIR100HG*, *VIM-AS1* and *VIM-AS1* v.2 upon TGF-β1 stimulation for 24 h. (**M**) RIP analysis using an anti-RanBP2 in A549 cells stimulated or not with TGF-β1 for 1, 3 and 24 h. Relative fold-enrichment of *VIM-AS1* associated with the RanBP2-specific RIP was normalized to the 0 h time point. Error bars represent ± SEM from three different experiments. RT-qPCR values in panels G, H and K represent fold-change of lncRNA expression normalized to *GAPDH*. P-value in I and M are shown based on one-way ANOVA, and in K and L are based on two-way ANOVA, followed by multiple paired comparisons conducted by means of Bonferroni's post-test method. P-values: $^{*}p \leq 0.05$; $^{**}p \leq 0.01$.

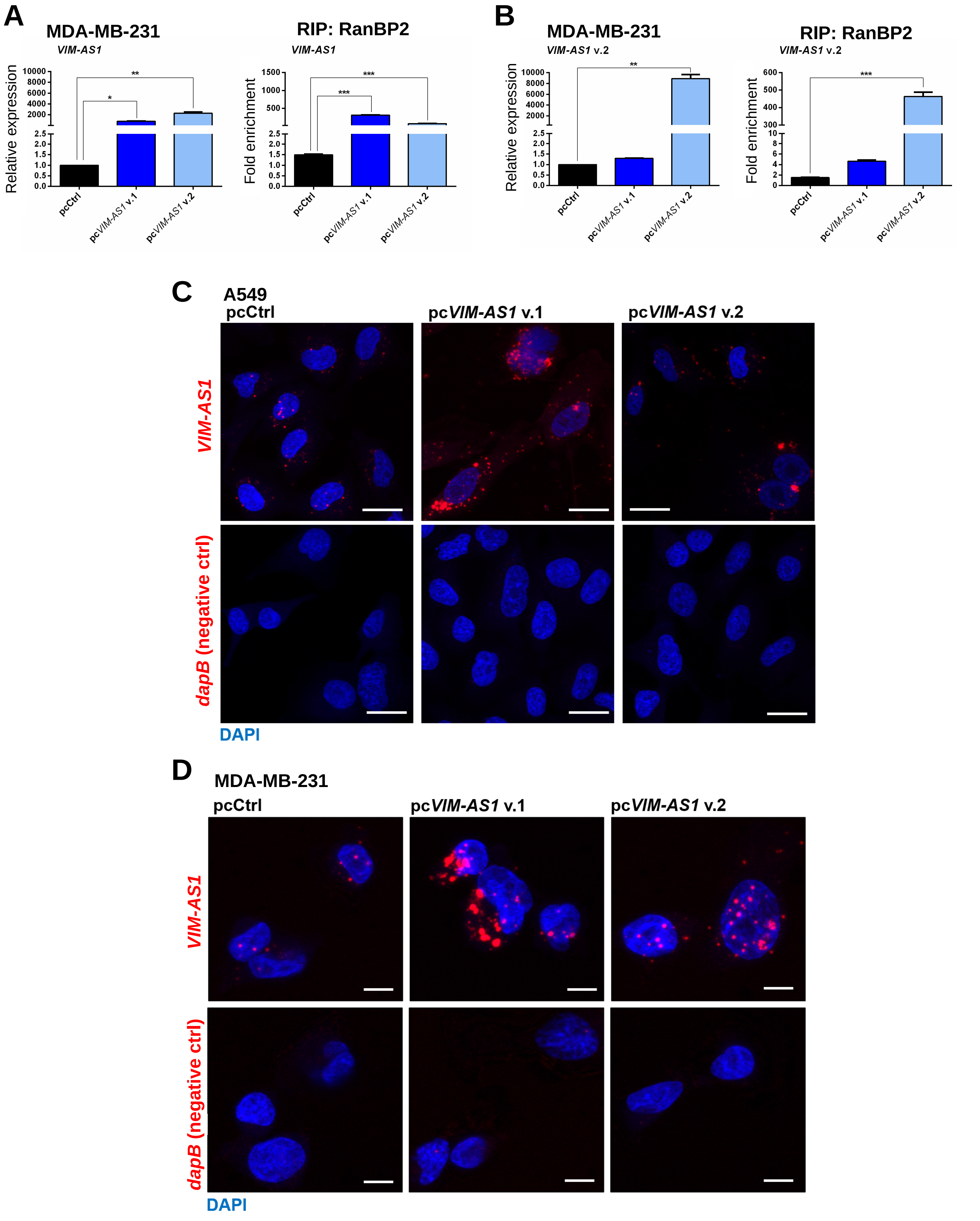


Suppl. Fig S16

**Supplementary Figure S16. Related to Fig. 5**. *VIM-AS1* binds to RanBP2 (Nucleoporin 358; Nup358). (**A, B**) RT-qPCR, followed by RanBP2-specific RIP analysis of the *VIM-AS1* (A: v.1 and v.2; B: v.2) levels in MDA-MB-231 cells transiently transfected with empty vector (pcCtrl) or full-length *VIM-AS1* v.1 or v.2. RT-qPCR values represent fold-change of *VIM-AS1* expression normalized to *GAPDH*, while RanBP2-specific RIP relative to the IgG control represents fold-enrichment. The data shown in panels A and B are presented as mean values of at least two biological replicates ± SEM, in technical triplicates and p-values are shown based on two-way ANOVA, followed by multiple paired comparisons conducted by means of Bonferroni's post-test method (*p ≤ 0.05; **p ≤ 0.01; ***p ≤ 0.001). (**C, D**) RNAscope for *VIM-AS1* and *dapB* (as negative control) in A549 (C) and MDA-MB-231 (D) cells transiently transfected with empty vector (pcCtrl) or full-length *VIM-AS1* v.1 or v.2. Scale bar 25 μm.

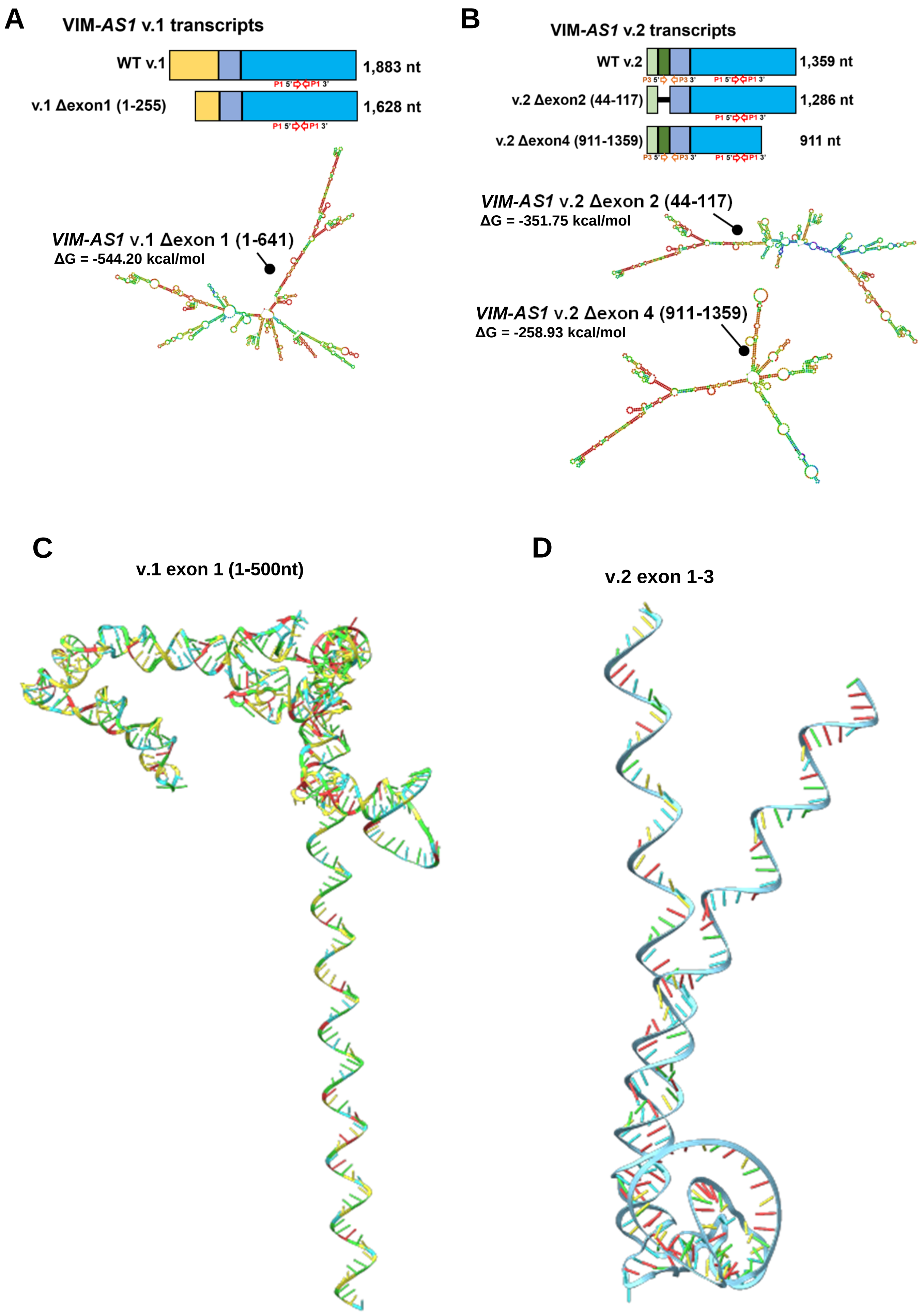


**Suppl. Fig S17**

**Supplementary Figure S17. Related to Fig. 5.** Characterization of *VIM-AS1* variant predicted structure. (**A, B**) Schematic representation of the organization of the *VIM-AS1* transcript and its variants v.1 (A) and v.2 (B) (i.e., v.1 Δexon-1, v.2 Δexon-2 and v.2 Δexon-4). Exons are shown as boxes, followed by the respective predicted secondary structure generated by RNAfold with the lowest required ΔG for each *VIM-AS1* v.1 (A) and v.2 (B). (**C, D**) Predicted RNA 3D structure of *VIM-AS1* v.1 exon-1 (C; 1-500 nt) and v.2 exons-1-3 (D) generated by RNAComposer.

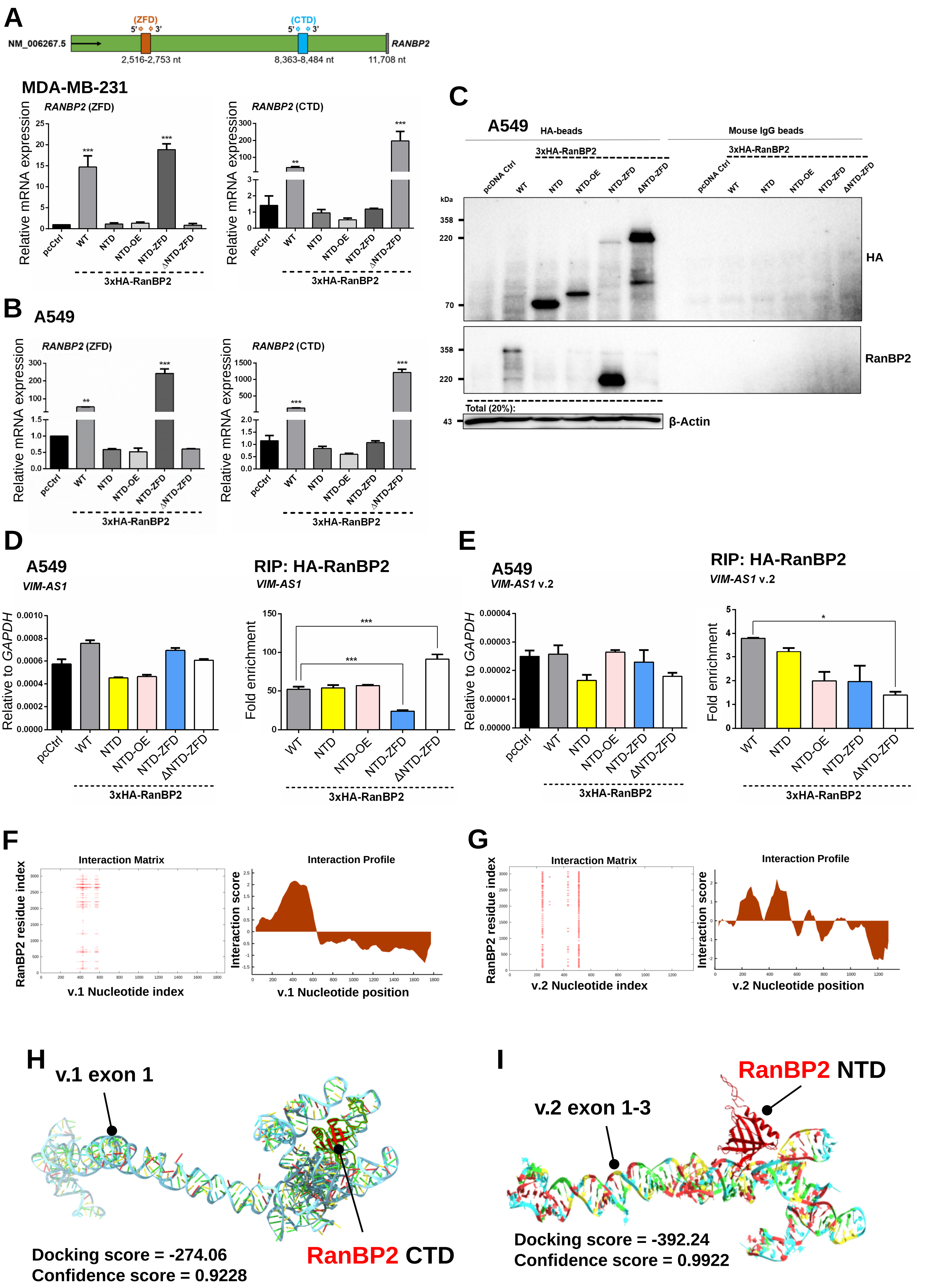


Suppl. Fig S18

**Supplementary Figure S18. Related to Fig. 5.** *VIM-AS1* variants bind to distinct RanBP2 domains. (**A, B**) Schematic outline of the primer sequences used to assess *RANBP2* mRNA expression (A). RT-qPCR analysis of *RANBP2* (ZFD or CTD pairs of primers) mRNA levels in MDA-MB-231 (A) and A549 (B) cells transiently transfected with empty vector (Ctrl) or the indicated pcDNA 3×HA-tagged RanBP2 variants. The RT-qPCR values are presented as the fold-change of RNA expression normalized to *GAPDH*, relative to the pcCtrl level, and shown as mean values of at least two biological replicates ± SEM, in technical triplicates. P-values are shown based on one-way ANOVA, followed by multiple paired comparisons conducted by means of Bonferroni's post-test method (**p ≤ 0.01; ***p ≤ 0.001). (**C**) 3×HA-RanBP2 protein expression in cellular extracts of A549 transfected cells. β-Actin was used as a loading control. Representative immunoblots of two independent biological replicates along with molecular mass (kDa) markers indicated. (**D, E**) RT-qPCR, followed by HA-specific RIP analysis of the *VIM-AS1* (D: v.1 and v.2; E: v.2) levels in A549 cells transiently transfected with full-length (WT) 3×HA-RanBP2 in addition to the indicated variants. RT-qPCR values represent fold-change of *VIM-AS1* expression normalized to *GAPDH*, while the HA-specific RIP relative to the IgG control represents fold-enrichment. The data are presented as mean values of at least two biological replicates ± SEM, in technical triplicates and p-values are shown based on one-way ANOVA, followed by multiple paired comparisons conducted using Bonferroni's post-test method. P-values: *p ≤ 0.05; ***p ≤ 0.001. (**F, G**) Prediction map of interaction between *VIM-AS1* v.1 (F) or v.2 (G) and RanBP2 based on their respective primary sequences using the catRAPID omics v2.0 algorithm. (**H, I**) Docking simulations of RanBP2 (red polypeptide) binding with *VIM-AS1* v.1 exon-1 (H) or v.2 exon-1-3 (I). The result representations indicate the lowest docking score and the highest confidence score of possible interactions among RanBP2 with SMAD2 or SMAD3. Predictions were generated using HDock.

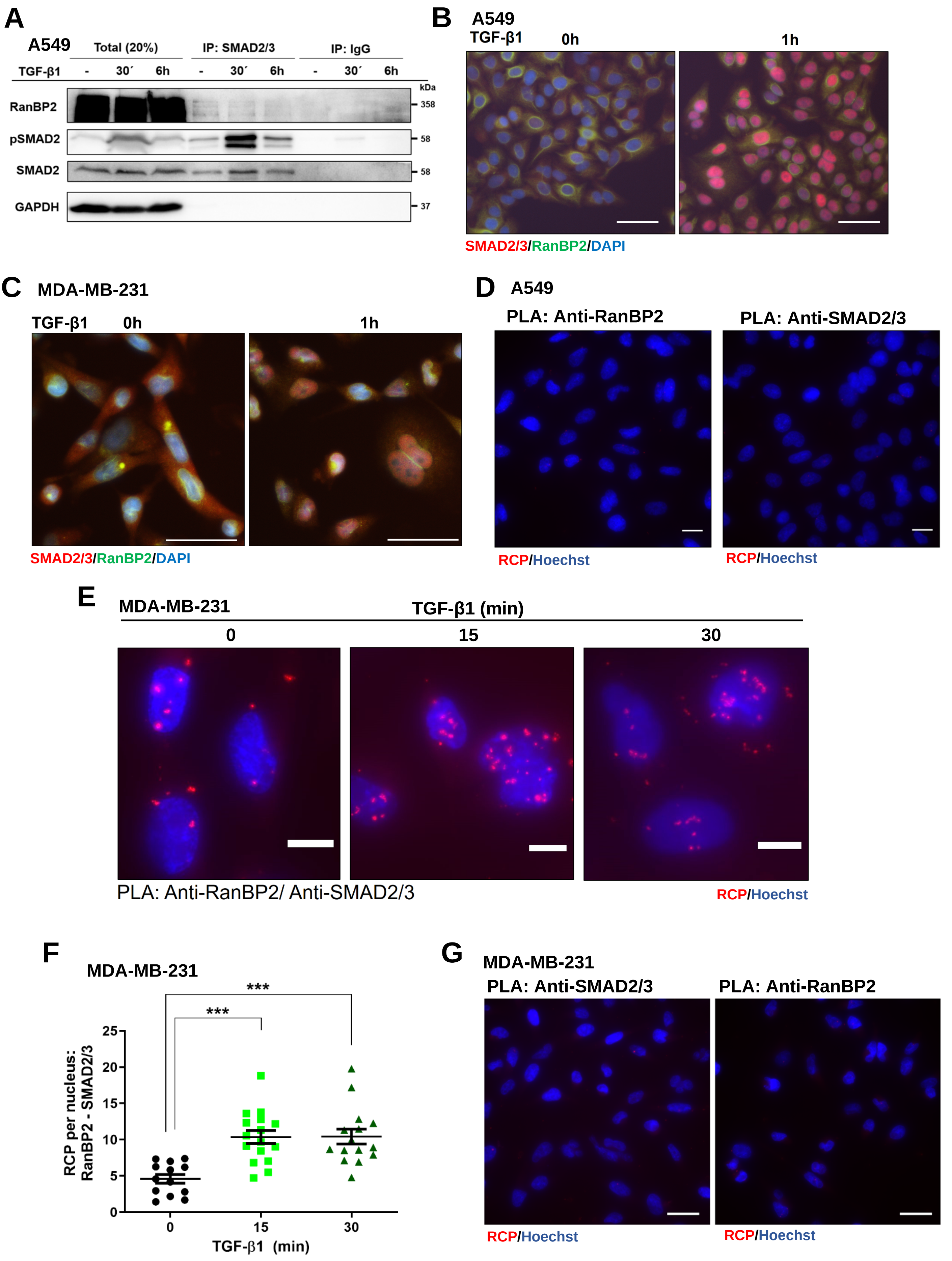


Suppl. Fig S19

**Supplementary Figure S19. Related to Fig. 6.** Enhanced SMAD interaction with RanBP2 upon TGF-β signaling activation. (**A**) Protein complex formation between SMAD2/3 and RanBP2. A549 cells were incubated with vehicle (-) or 5 ng/mL TGF-β1 for 30 min and 6 h, and the total protein lysates were immunoprecipitated (IP) with SMAD2/3-specific antibody or non-specific IgG, followed by SDS-PAGE and immunoblot with antibodies against RanBP2 (lower and higher exposure), pSMAD2 and SMAD2 and GAPDH. The Input corresponds to 20% of total protein used for IP. Representative immunoblots of three independent biological replicates along with molecular mass markers in kDa are shown. (**B, C**) Representative immunofluorescence microscopy pictures of A549 (B) and MDA-MB-231 (C) cells stimulated or not with 5 ng/mL TGF-β1 for 1 h. The SMAD2/3 (red), RanBP2 (green) and nuclei (DAPI; blue) are labeled. (**D**) Negative control with single antibody incubation for the PLA used to validate the co-localization of SMAD2/3 with RanBP2 in A549 cells. Nuclei are shown in blue (Hoechst), and PLA rolling circle amplification product in red. (**E**) PLA used to show the co-localization of SMAD2/3 with RanBP2 in MDA-MB-231 cells incubated or not with 5 ng/mL TGF-β1 for 15 or 30 min. Nuclei are shown in blue (Hoechst), and PLA rolling circle amplification product in red. (**F**) Quantification for the co-localization between SMAD2/3 with RanBP2 in MDA-MB-231 cells is shown and presented as mean values of individual micrographs from at least two biological replicates ± SEM. (**G**) Negative control with single antibody incubation for the PLA used to validate the co-localization of SMAD2/3 with GATA6 or SPI1 in MDA-MB-231 cells. Nuclei are shown in blue (Hoechst), and PLA rolling circle amplification product in red.

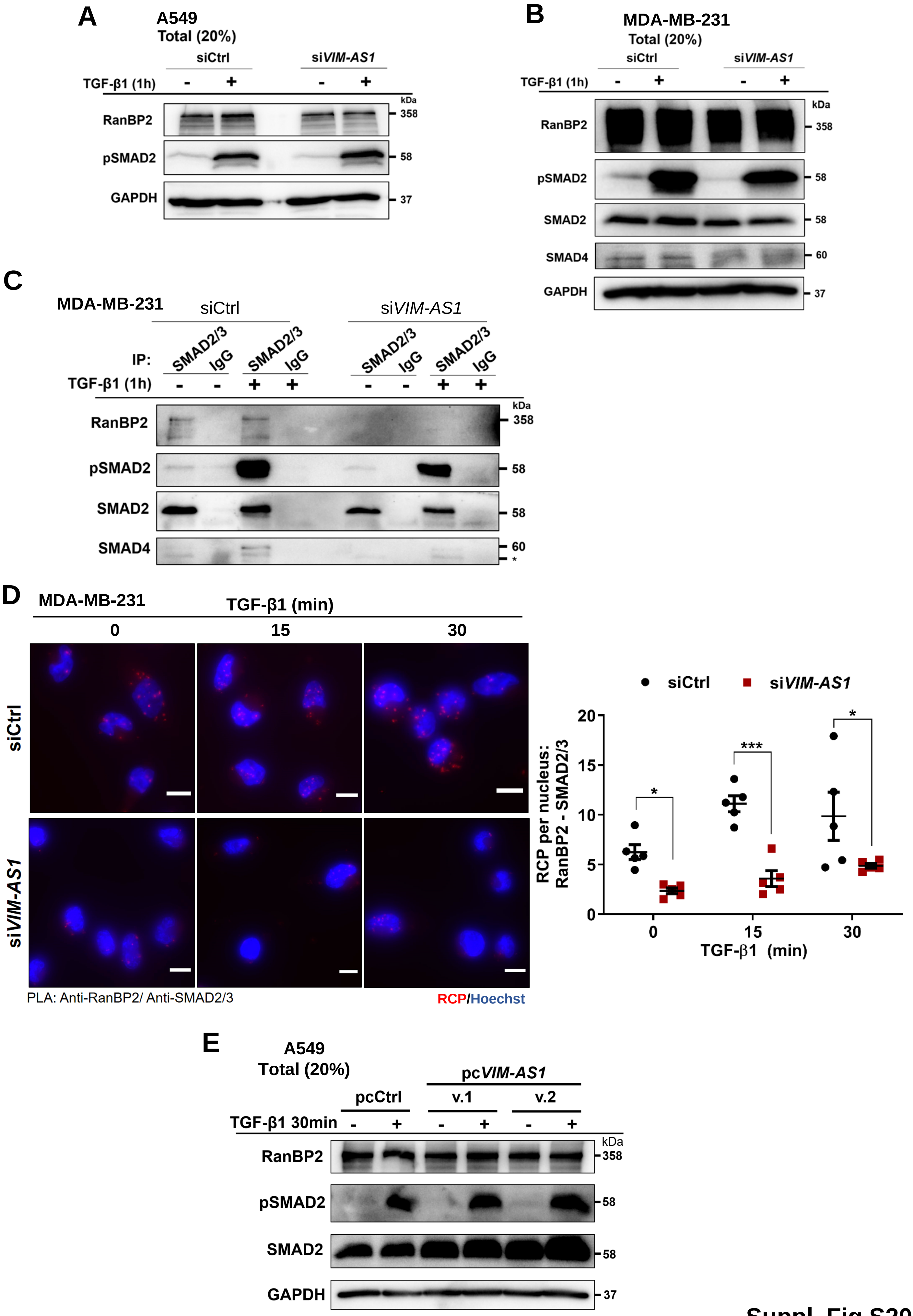


Suppl. Fig S20

**Supplementary Figure S20. Related to Fig. 6.** SMAD nuclear accumulation relies on RanBP2 and *VIM-AS1*. (**A**) Input corresponding to 20% of total protein used for immunoprecipitation (IP) in Figure 6B. Protein complex formation between SMAD2/3 with RanBP2. A549 cells transiently transfected with control siRNA (siCtrl) or si*VIM-AS1* were incubated with vehicle (-) or 5 ng/mL TGF-β1 for 1 h, followed by SDS-PAGE and immunoblot with RanBP2, pSMAD2 and GAPDH antibodies. Representative immunoblots of three independent biological replicates along with molecular mass markers in kDa are shown. (**B, C**) Protein complex formation between SMAD2/3 with RanBP2. MDA-MB-231 cells transiently transfected with control siRNA (siCtrl) or si*VIM-AS1* were incubated with vehicle (-) or 5 ng/mL TGF-β1 for 1 h and the total protein lysates (B; Input 20%) were immunoprecipitated with SMAD2/3-specific antibody or non-specific IgG, followed by SDS-PAGE and immunoblot with RanBP2, pSMAD2, SMAD2 and SMAD4 antibodies (C). Representative immunoblots of three independent biological replicates along with molecular mass markers in kDa are shown. (**D**) PLA was used to show the co-localization of SMAD2/3 with RanBP2 in MDA-MB-231 cells transiently transfected with siCtrl or si*VIM-AS1* and incubated with vehicle (0 h) or 5 ng/mL TGF-β1 for 15 or 30 min. Nuclei are shown in blue (Hoechst), and PLA rolling circle amplification product is in red. Quantification for the co-localization between SMAD2/3 with RanBP2 in MDA-MB-231 transfected cells is shown and presented as mean values of individual micrographs from at least two biological replicates ± SEM. P-values are shown based on two-way ANOVA, followed by multiple paired comparisons conducted using Bonferroni's post-test method: $*p \leq 0.05$; $***p \leq 0.001$. (**E**) Input corresponding to 20% of total protein used for immunoprecipitation (IP) in Figure 6D. Protein complex formation between SMAD2/3 with RanBP2. A549 cells transfected with empty vector (Ctrl) or pcDNA(pc)-*VIM-AS1* v.1 or pc*VIM-AS1* v.2 upon selection with neomycin, incubated with vehicle (-) or 5 ng/mL TGF-β1 for 30-min, followed by SDS-PAGE and immunoblot with RanBP2, pSMAD2, SMAD2 and GAPDH antibodies. Representative immunoblots of three independent biological replicates along with molecular mass markers in kDa are shown.

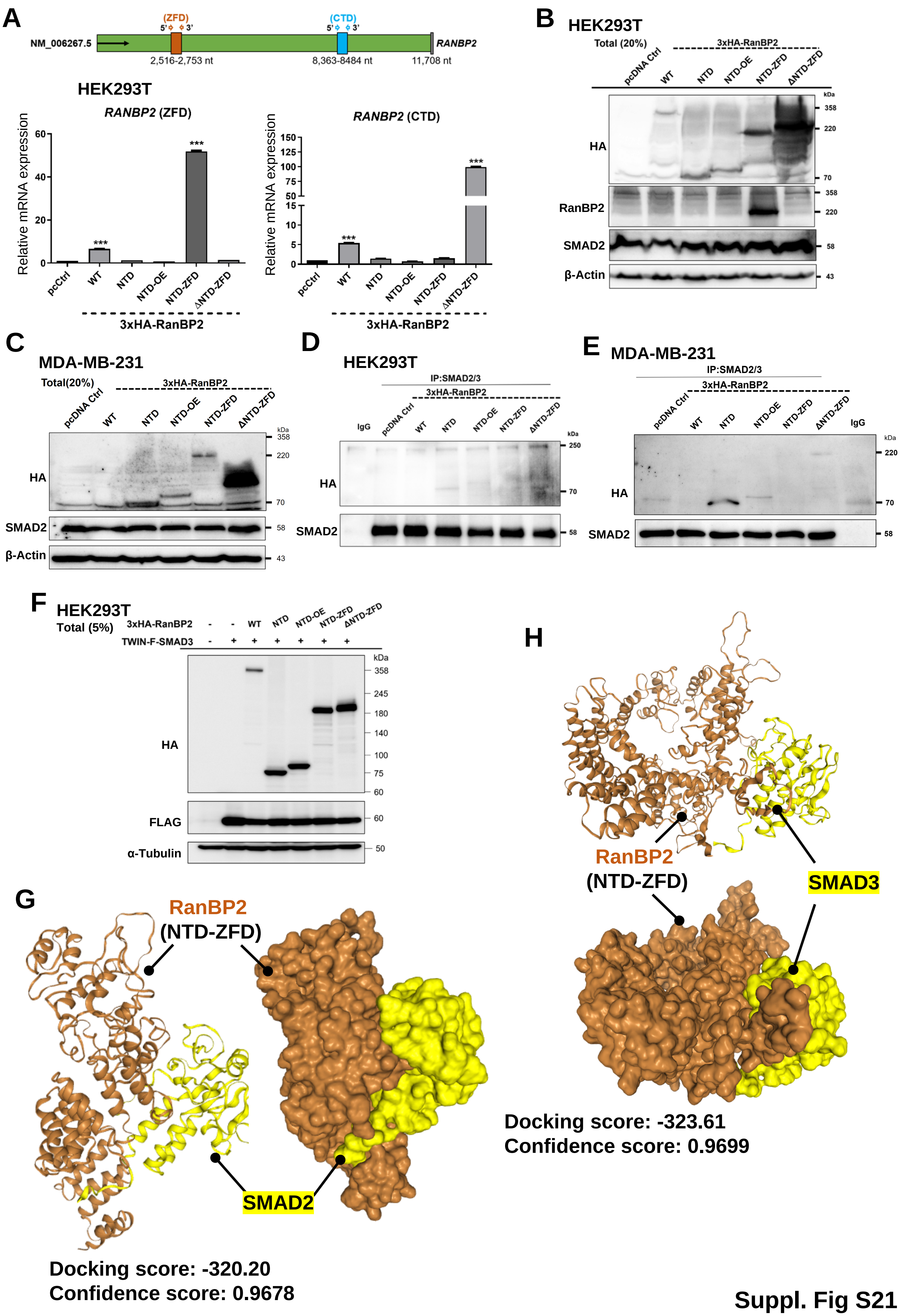


Suppl. Fig S21

**Supplementary Figure S21. Related to Fig. 6.** Characterization of SMAD interaction with RanBP2 domains. (**A**) Schematic outline of the primer sequences used to assess *RANBP2* mRNA expression. RT-qPCR analysis of *RANBP2* (ZFD or CTD pairs of primers) mRNA levels in HEK293T cells transiently transfected with empty vector (Ctrl) or the indicated pcDNA 3×HA-tagged RanBP2 variants. The RT-qPCR values are presented as the fold-change of RNA expression normalized to *GAPDH*, relative to the pcCtrl level, and shown as mean values of at least two biological replicates ± SEM, in technical triplicates. P-values are shown based on one-way ANOVA, followed by multiple paired comparisons conducted by means of Bonferroni's post-test method (***p ≤ 0.001). (**B, C**) Input corresponding to 20% of total protein used for immunoprecipitation (IP) in panel D and E. Protein complex formation between SMAD2/3 with 3×HA-tagged RanBP2 variants. The total protein lysates of transfected HEK293T (B) and MDA-MB-231 (C) cells with the indicated 3×HA-tagged RanBP2 variants were followed by SDS-PAGE and immunoblotting with HA, RanBP2, SMAD2 and β-Actin antibodies. (**D, E**) Protein complex formation between SMAD2/3 with 3×HA-tagged full length RanBP2 and deletion mutants. The total protein lysates of transfected HEK293T (D) MDA-MB-231 (E) cells with the indicated 3×HA-tagged full length RanBP2 and deletion mutants (B, C; Input 20%) were immunoprecipitated (D, E) with SMAD2/3-specific antibody or non-specific IgG, followed by SDS-PAGE and immunoblotting with HA and SMAD2 antibodies. Representative immunoblots of three independent biological replicates along with molecular mass markers in kDa are shown. (**F**) Input corresponding to 5% of total protein used for immunoprecipitation (IP) used in Fig. 6E. Protein complex formation between TWIN-FLAG(F)-SMAD3 with 3×HA-tagged RanBP2 full length or fragmented in HEK293T cells stimulated with TGF-β1 for 1 h. The total protein lysates of transfected HEK293T cells were followed by SDS-PAGE and immunoblotting with HA and FLAG antibodies. (**G, H**) Docking simulations of RanBP2 (tan) binding with SMAD2 (G; yellow) or SMAD3 (H; yellow). Results representations indicate the lowest docking score and highest confidence score of possible interactions among RanBP2 with SMAD2 or SMAD3. Predictions were generated using HDock.

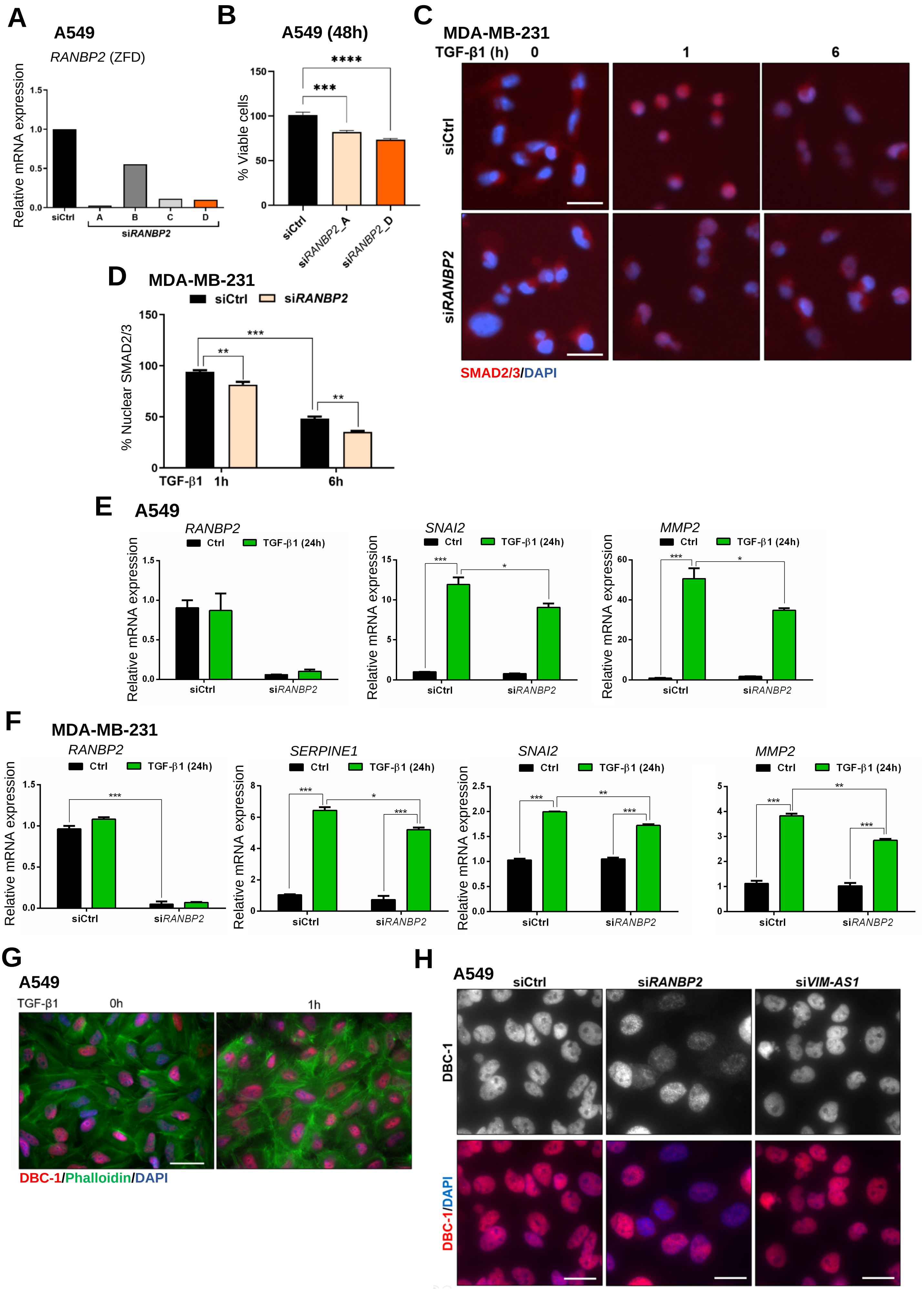


Suppl. Fig S22

**Supplementary Figure S22. Related to Fig. 6.** RanBP2 enhances SMAD2/3 nuclear accumulation. (**A**) RT-qPCR analysis of *RANBP2 (ZFD)* mRNA levels in A549 cells transiently transfected with control siRNA (siCtrl) or the indicated siRNAs against *RANBP2* (note that siRANBP2_A and _D were the most efficient). (**B**) Cell viability assay of A549 cells transiently transfected with control siRNA (siCtrl) or si*RANBP2* for 48 h, and normalized against the siCtrl cells. (**C, D**) Representative immunofluorescence microscopy pictures of MDA-MB-231 cells transfected with the indicated control siRNAs (siCtrl) or siRNA against *RANBP2*_A, and stimulated with vehicle (0 h) or 5 ng/mL TGF-β1 for 1 or 6 h. The quantification in D represents the percentage of SMAD2/3-stained nuclear intensity, normalized to the 0 h time point. Scale bars, 100 μm. (**E, F**) RT-qPCR analysis of the indicated mRNA levels in A549 (E) and MDA-MB-231 (F) cells, transiently transfected with siCtrl or si*RANBP2_A* and incubated with vehicle (0 h) or 5 ng/mL TGF-β1 for 24 h. Data in B, D, E and F are presented as mean values of three biological replicates ± SEM, each in technical triplicates. P-values in panel B is based on one-way ANOVA, while panels D-F are shown based on two-way ANOVA, followed by multiple paired comparisons conducted by means of Bonferroni's post-test method. P-values: *p ≤ 0.05; **p ≤ 0.01; ***p ≤ 0.001; ****p ≤ 0.0001. (**G, H**) Representative immunofluorescence microscopy pictures of DBC-1 in A549 cells stimulated with TGF-β1 or not for 1 h (G) or transiently transfected with siCtrl, si*RANBP2* or si*VIM-AS1* (H). DBC-1 (red or black and white), phalloidin (green) and nuclei (DAPI; blue) are labeled. Scale bar 50 (G) and 25 (H) μm.

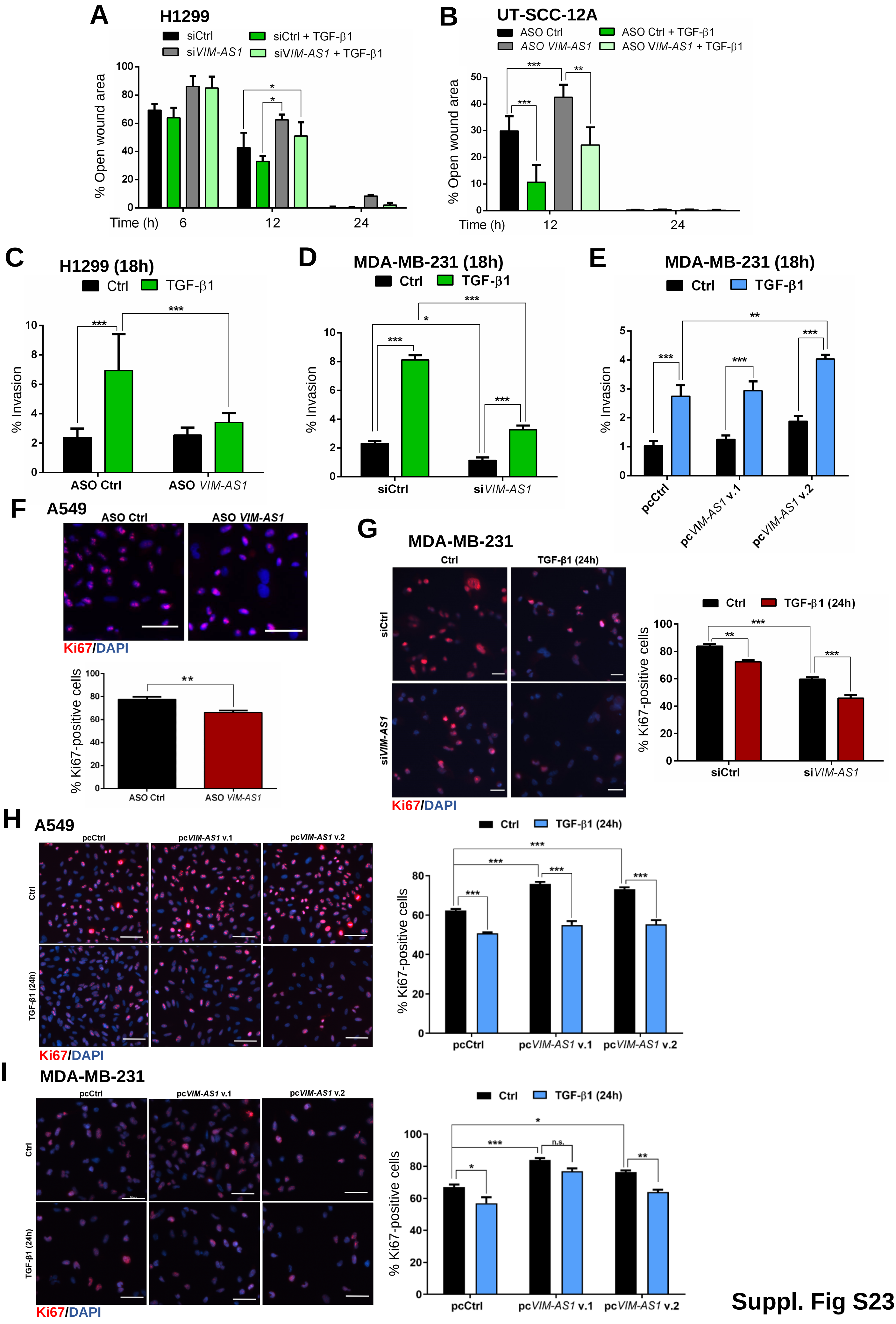


Suppl. Fig S23

**Supplementary Figure S23. Related to Fig. 7.** Impact of *VIM-AS1* on motility and proliferation of tumor cells. (**A, B**) Cell culture wound healing assay with H1299 cells transiently transfected with control siRNA (siCtrl) or si*VIM-AS1* (A) and UT-SCC-12A transiently transfected with ASO Ctrl or ASO *VIM-AS1* (B) stimulated or not with vehicle (Ctrl) or 5 ng/mL TGF-β1 for the indicated periods. The data are presented as percent open wound area and plotted as mean values of three biological replicates ± SEM, in technical triplicates and p-values are shown based on two-way ANOVA, followed by multiple paired comparisons conducted by means of Bonferroni's post-test method. (**C-E**) Matrigel invasion assay in trans-wells with H1299 cells transiently transfected with control ASO (Ctrl) or ASO *VIM-AS1* (C) and MDA-MB-231 cells transiently transfected with siCtrl or si*VIM-AS1* (D) or transfected with empty vector (Ctrl) or pcDNA(pc)-*VIM-AS1* v.1 or pc*VIM-AS1* v.2 upon selection with neomycin (E), followed by incubation with 5 ng/mL TGF-β1 for 18 h. The data represent the invaded cells as a percent of the total cell number and are plotted as mean values of three biological replicates ± SEM, in technical triplicates. (**F-I**) Representative immunofluorescence microscopy pictures of A549 cells transiently transfected with control ASO Ctrl or ASO *VIM-AS1* (F), MDA-MB-231 cells transiently transfected with control siCtrl or si*VIM-AS1* and stimulated with vehicle (Ctrl) or 5 ng/mL TGF-β1 for 24 h (G), in addition to A549 (H) and MDA-MB-231 (I) cells transfected with empty vector (Ctrl) or pcDNA(pc)-*VIM-AS1* v.1 or pc*VIM-AS1* v.2 upon selection with neomycin. The proliferation marker Ki67 (red) and nuclei (DAPI; blue) are labeled. Scale bars, 50 µm. Quantification of Ki67-positive cells expressed as percent of positive cells relative to the total number of cells under each condition, and plotted as mean values of three biological replicates ± SEM, each in technical duplicates and p-values are shown based on two-way ANOVA, followed by multiple paired comparisons conducted by means of Bonferroni's post-test method. P-values: *p ≤ 0.05; **p ≤ 0.01; ***p ≤ 0.001.

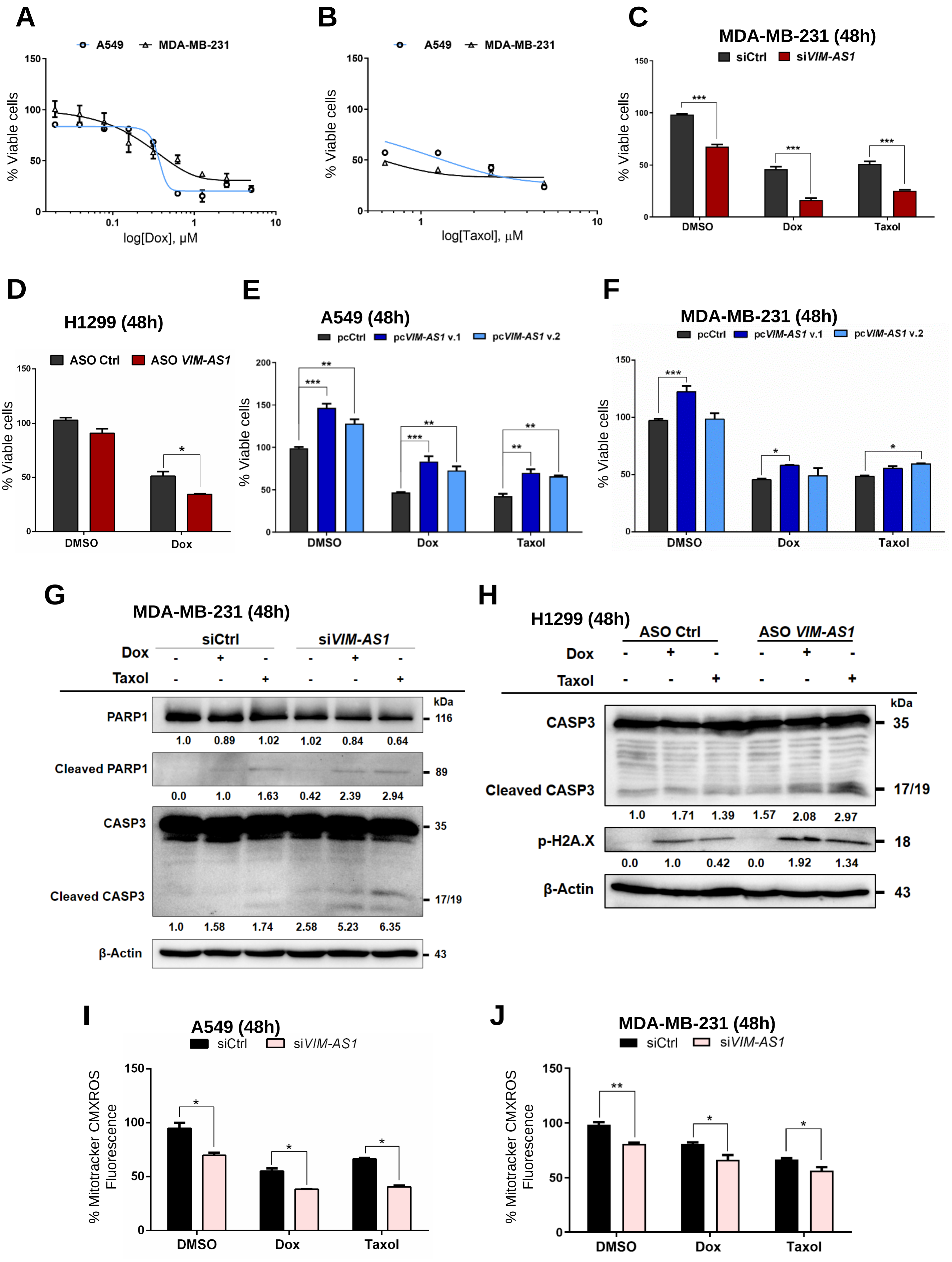


**Suppl. Fig S24**

**Supplementary Figure S24. Related to Fig. 7.** Silencing *VIM-AS1* sensitizes tumor cells to cytotoxic drugs. (**A, B**) Viability curves of A549 and MDA-MB-231 cells in the presence of increasing concentrations of doxorubicin (dox; A) and taxol (B) (logarithmic scale). Note the lack of significant differences. (**C-F**) Cell viability assay of MDA-MB-231 cells transiently transfected with control siRNA (siCtrl) or si*VIM-AS1* (C), H1299 transiently transfected with ASO Ctrl or ASO *VIM-AS1* (D), A549 (E) and MDA-MB-231 (F) cells transfected with empty vector (Ctrl) or pcDNA(pc)-*VIM-AS1* v.1 or pc*VIM-AS1* v.2 upon selection with neomycin, followed by the presence or absence of DMSO (Ctrl), 0.5 μM Dox or 0.5 μM Taxol for 48 h and normalized against the vehicle-treated Ctrl cells (**G, H**) Representative immunoblot of cleaved and total Caspase-3 (CASP3) or PARP1 in MDA-MB-231 cells transiently transfected with control siRNA (siCtrl) or si*VIM-AS1* and cleaved and total CASP3 and p-H2A.X in H1299 cells transiently transfected with ASO Ctrl or ASO *VIM-AS1* (H) in the presence of DMSO or 0.5 μM Dox or Taxol for 48 h. β-Actin was used as a loading control, and molecular mass (kDa) markers are indicated along with densitometric values of normalized band intensity only in the lanes where the relevant protein markers scored positively. (**I, J**) Mitochondrial transmembrane potential of A549 (I) and MDA-MB-231 (J) cells treated with 0.5 μM Dox or Taxol for 48 h, assessed by MitoTracker CMXROS and normalized against the vehicle-treated control cells (DMSO). The data in panels C-F, I and J are presented as mean values of three biological replicates ± SEM, in technical triplicates and p-values are shown based on two-way ANOVA, followed by multiple paired comparisons conducted by means of Bonferroni's post-test method. P-values: *p ≤ 0.05; **p ≤ 0.01; ***p ≤ 0.001.
